# Control of zeolite microenvironment for propene synthesis from methanol

**DOI:** 10.1038/s41467-021-21062-1

**Published:** 2021-02-05

**Authors:** Longfei Lin, Mengtian Fan, Alena M. Sheveleva, Xue Han, Zhimou Tang, Joseph H. Carter, Ivan da Silva, Christopher M. A. Parlett, Floriana Tuna, Eric J. L. McInnes, German Sastre, Svemir Rudić, Hamish Cavaye, Stewart F. Parker, Yongqiang Cheng, Luke L. Daemen, Anibal J. Ramirez-Cuesta, Martin P. Attfield, Yueming Liu, Chiu C. Tang, Buxing Han, Sihai Yang

**Affiliations:** 1 Department of Chemistry, University of Manchester, Manchester, UK; 2 Photon Science Institute, University of Manchester, Manchester, UK; 3 Shanghai Key Laboratory of Green Chemistry and Chemical Processes, School of Chemistry and Molecular Engineering, East China Normal University, Shanghai, China; 4 Diamond Light Source, Harwell Science and Innovation Campus, Didcot, Oxfordshire UK; 5 ISIS Facility, STFC Rutherford Appleton Laboratory, Chilton, Oxfordshire UK; 6 Department of Chemical Engineering and Analytical Science, University of Manchester, Manchester, UK; 7 University of Manchester at Harwell, Diamond Light Source, Didcot, Oxfordshire UK; 8 UK Catalysis Hub, Research Complex at Harwell, Didcot, Oxfordshire UK; 9 Instituto de Tecnologia Quimica, UPV-CSIC Universidad Politecnica de Valencia, Valencia, Spain; 10 Neutron Scattering Division, Oak Ridge National Laboratory, Oak Ridge, TN USA; 11 Beijing National Laboratory for Molecular Sciences, CAS Key Laboratory of Colloid, Interface and Chemical Thermodynamics, Institute of Chemistry, Chinese Academy of Science, Beijing, China

**Keywords:** Catalysis, Inorganic chemistry

## Abstract

Optimising the balance between propene selectivity, propene/ethene ratio and catalytic stability and unravelling the explicit mechanism on formation of the first carbon–carbon bond are challenging goals of great importance in state-of-the-art methanol-to-olefin (MTO) research. We report a strategy to finely control the nature of active sites within the pores of commercial MFI-zeolites by incorporating tantalum(V) and aluminium(III) centres into the framework. The resultant TaAlS-1 zeolite exhibits simultaneously remarkable propene selectivity (51%), propene/ethene ratio (8.3) and catalytic stability (>50 h) at full methanol conversion. In situ synchrotron X-ray powder diffraction, X-ray absorption spectroscopy and inelastic neutron scattering coupled with DFT calculations reveal that the first carbon–carbon bond is formed between an activated methanol molecule and a trimethyloxonium intermediate. The unprecedented cooperativity between tantalum(V) and Brønsted acid sites creates an optimal microenvironment for efficient conversion of methanol and thus greatly promotes the application of zeolites in the sustainable manufacturing of light olefins.

## Abstract (web summary)

Lower olefins are mainly produced from fossil resources and the methanol-to-olefins process offers a new sustainable pathway. Here, the authors show a new zeolite containing tantalum and aluminium centres which shows simultaneously high propene selectivity, catalytic activity, and stability for the synthesis of propene.

## Introduction

Propene is mass produced as a key light olefin for the synthesis of a wide spectrum of vital polymers and fine chemicals^1^. However, a global propene shortage is predicted^2–4^ (supply vs. demand is 85 vs. 118 million tonnes in 2021) due to the rapidly growing demand for propene (projected propene market will reach $137 billion by 2027) alongside the low propene (P)/ethene (E) ratio (typically P/E < 1) that is achieved in the state-of-the-art naphtha cracking processes. Methanol-to-olefins (MTO) processes^5–12^ can mitigate this short fall so have received tremendous interest from both academia and industry because they enable the production of light olefins from methanol, a feedstock widely obtainable from biomass, waste, coal, natural gas and even carbon dioxide^13^, that will enable future sustainable manufacturing processes. Commercial MTO plants use ZSM-5 and SAPO-34 zeolites as catalysts and yield multiple products via complex, and still debated reaction pathways^5,10^. More than 20 different, sometimes controversial, formation mechanisms of the first carbon–carbon bond have been postulated to date, including the carbene mechanism^14^, the methane–formaldehyde mechanism^15^, the methyleneoxy mechanism^16^, the methoxymethyl cation mechanism^17^, the Koch carbonylation mechanism^18^, and the methane–Al/oxonium mechanism^19^. Thus, understanding the precise reaction mechanism and developing efficient catalysts that achieve the optimal balance between propene selectivity, P/E ratio and catalytic stability in MTO processes remain unsolved problems^20^.

**Fig. 1:**
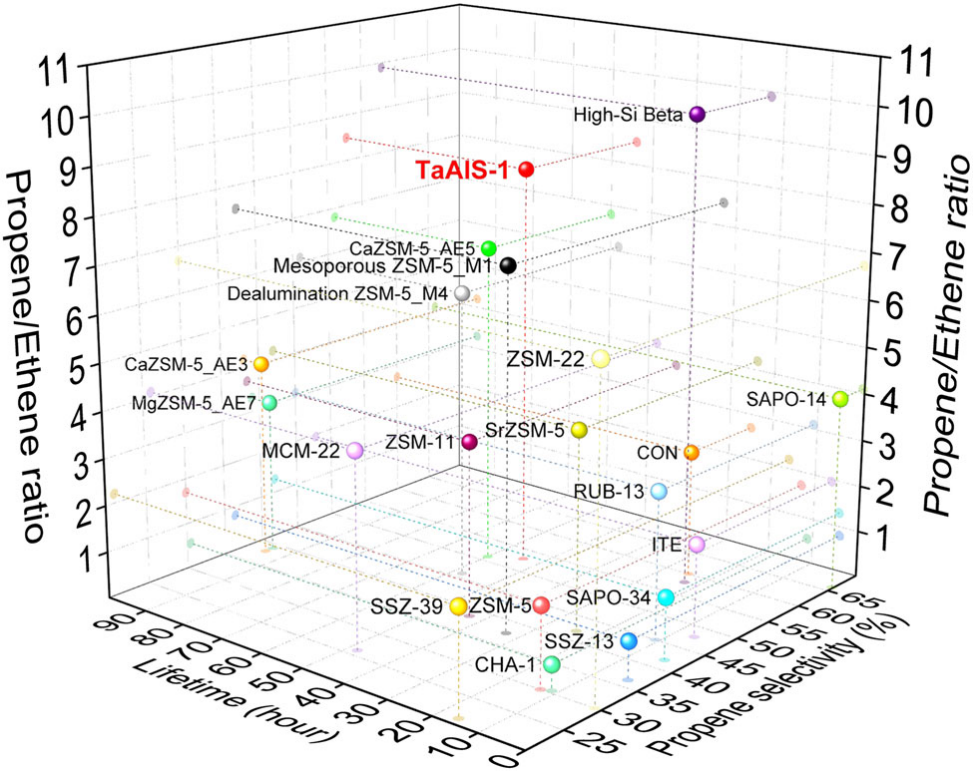
Comparison of propene selectivity-propene/ethene ratio-catalytic stability of selected MTO catalysts. 3D plot of P/E ratio versus lifetime and propene selectivity, showing the balance of catalytic performance of selected zeolites for conversion of pure methanol to olefins at methanol conversion ≥95%. For clarity, only representative zeolites are shown in the plot. Full data are listed in Supplementary Table 1.

Current top-performing MTO catalysts include: M–ZSM-5 (M = Mg, Ca, Sr) affording propene selectivities of 38–51%, P/E ratios of 3.3–6.8 and catalyst lifetime of 24–95 h^6^; high-silica *Beta* zeolite exhibiting a high propene selectivity of 58%, a P/E ratio of 9.8 and catalyst lifetime of 23 h^11^; RUB-13 zeolite with precise cage cavity showing propene selectivity of 45%, P/E ratio of 3.0 and lifetime of 11 h at 95% conversion of methanol^8^; CON-type zeolite giving high propene selectivity of 60% and lifetime of 25 h, but a low P/E ratio of 2.7^21^; SAPO-14 zeolites with small pores exhibiting high propene selectivity of 66% and P/E ratio of 4.1 at full conversion of methanol, but having a short lifetime of ~0.3 h^9^. Small pore catalysts generally suffer from severe deactivation due to the rapid blockage of pores by coke. Considering all three indexes, i.e., propene selectivity–P/E ratio–catalytic stability, Ca–ZSM-5 and high-silica *Beta* zeolite are promising candidates for catalysing MTO reactions (Fig. 1), while new zeolites, other than ZSM-5 and SAPO-34, have yet to be commercialised owing to the uncertainty around their large-scale synthesis and applicability in the existing industrial infrastructure.

We have recently developed a Nb-containing zeolite^22^ which shows excellent performance in the cleavage of C–O bonds in γ-valerolactone via its efficient activation over Nb(V) sites. However, the weak acidity of this material restricts the formation of carbon–carbon bonds in MTO reactions with a nil (<1%) selectivity to olefins (Supplementary Table 1). Tantalum is extremely electrophilic and can drive the formation and cleavage of carbon–carbon bonds during the metathesis of alkanes^23^. However, its catalytic performance in MTO reactions has not been explored and the precise catalytic role of tantalum remains unclear. Here we report control of the microenvironment of ZSM-5 via incorporating Ta(V) and Al(III) centres into the framework to afford a new hetero-atomic zeolite (denoted as TaAlS-1) to achieve simultaneously high propene selectivity (>50%), P/E ratios (up to 11) and high stability (>50 h) in MTO process. A series of *operando* studies employing synchrotron X-ray diffraction and absorption spectroscopy and neutron spectroscopy confirm that an unprecedented cooperativity between Ta(V) and Brønsted acid sites results in efficient activation of adsorbed methanol and the formation of trimethyloxonium (TMO) intermediate species, thereby promoting the formation of the first carbon–carbon bond in propene. The multifunctional microenvironment of TaAlS-1 has enabled effective control of product selectivity while sustaining the high catalytic stability of ZSM-5, thus reinforcing its potential for the production of propene from methanol.

## Results

### Synthesis and characterisation

**Fig. 2:**
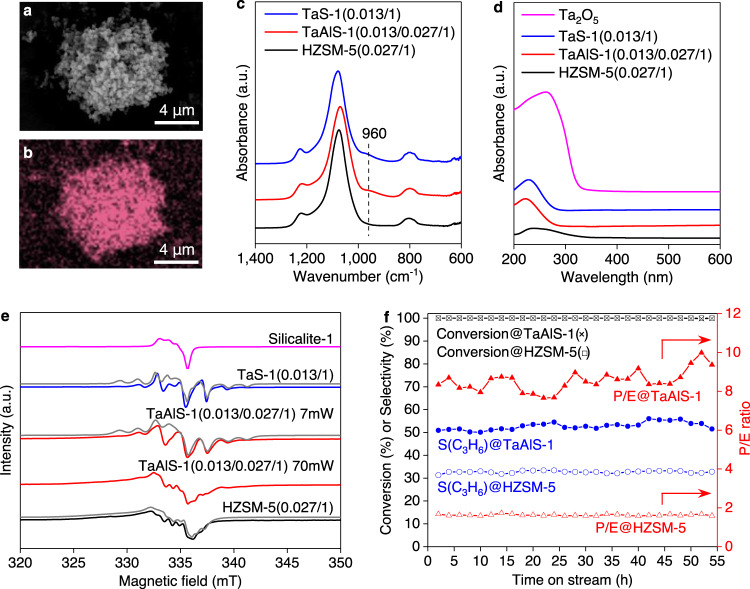
Physical characterisation and stability of catalysts. **a** SEM image of TaAlS-1(0.013/0.027/1), **b** EDX map of Ta L_ɑ_ in TaAlS-1(0.013/0.027/1). **c** FTIR and **d** UV–vis spectra of HZSM-5(0.027/1), TaAlS-1(0.013/0.027/1) and TaS-1(0.013/1) samples. **e** X-band (9.4 GHz) CW EPR spectra recorded at 40 K of vacuum-sealed zeolites activated by γ-irradiation at 77 K. The spectra of Silicalite-1, TaS-1 and HZSM-5 were recorded with a microwave power of 7 mW. Simulated line shapes are shown in grey; parameters for simulation are presented in Supplementary Table 3. **f** Lifetime study of TaAlS-1(0.013/0.027/1) and HZSM-5(0.027/1) at 400 °C, WHSV 0.16 h^−1^. S(C_3_H_6_) = propene selectivity. The yield of propene on TaAlS-1 (0.013/0.027/1) remained at >50% after 54 h of continuous reaction.

A range of ZSM-5 samples containing Ta(V) (0–3.7 wt%) and Al(III) (0–1.1 wt%) were synthesised by hydrothermal reactions. All obtained materials exhibit the MFI-type framework (Supplementary Fig. 1), Brunauer–Emmett–Teller (BET) surface areas of 402–429 m^2^ g^−1^ (Supplementary Table 2), and particle size distributions of 300–500 nm (Supplementary Fig. 2, Supplementary Table 2). The atomic ratio of Ta/Al/Si was determined by energy-dispersive X-ray (EDX) analysis, which confirmed the existence of metal sites in the zeolites (Fig. 2a, b, Supplementary Fig. 3). The vibrational modes of Ta–O–Si moieties in TaAlS-1(0.013/0.027/1) are observed by FTIR at 960 cm^−1^ (Fig. 2c) and a transition at 220 nm observed in the UV–vis spectrum of TaAlS-1(0.013/0.027/1) (Fig. 2d), indicating the incorporation of Ta(V) centres at framework T-sites and the absence of aggregation of Ta(V) sites. The ^27^Al NMR spectrum of TaAlS-1(0.013/0.027/1) shows that Al(III) centres are incorporated into the framework with an absence of Al_2_O_3_ (Supplementary Fig. 4).

Electron paramagnetic resonance (EPR) spectroscopy has also confirmed the successful incorporation of Ta(V) and Al(III) into framework sites. Although TaS-1 (the isostructural analogue with no Al) and TaAlS-1 are EPR silent due to the diamagnetic Ta(V) and Al(III) ions, characteristic paramagnetic centres can be induced by γ-irradiation of the zeolites^24^. Initial signals of γ-irradiated TaS-1 and TaAlS-1 samples were assigned to electron–hole defects on a framework oxygen atom in Ta–O^•^–Si sites. The assignment was derived from the analysis of quadrupole and hyperfine coupling with the ^181^Ta nucleus (*I* = 7/2; 99.98% natural abundance; Fig. 2e and Supplementary Table 3). Saturation of the EPR signal^25^ of TaAlS-1 sample by increasing the microwave power from 7 to 70 mW revealed the appearance of a characteristic signal for Al–O^•^–Si sites^26^ (Fig. 2e), which is also confirmed by the Pulse HYSCORE spectroscopy (HYSCORE = hyperfine sub-level correlation; Supplementary Note 1 EPR spectroscopy, Supplementary Fig. 5 and Supplementary Table 4). The signals of Al–O^•^–Si and Ta–O^•^–Si defect sites are absent in TaS-1 and HZSM-5 samples, respectively (Supplementary Fig. 6). Thus, EPR measurements provide convincing evidence for the incorporation of both Ta(V) and Al(III) centres into the framework of TaAlS-1, enabling the effective control of the active sites within the microenvironment.

The nature of acidity in these zeolites was studied by pyridine-adsorption infra-red spectroscopy (Py-IR) and further quantified by ammonia temperature-programmed desorption (NH_3_-TPD) (Supplementary Figs. 7 and 8). TaS-1 exhibits solely Lewis acidity due to the presence of Ta(V), whereas TaAlS-1 shows both Lewis and Brønsted acidity that arises from the dual-functionality of Ta(V) and Al(III) sites. Notably, the nature of the acidity in TaAlS-1 is distinct from that of HZSM-5: the ratio of Lewis/Brønsted acid sites in TaAlS-1 is 7.8 (Supplementary Table 5), 20 times that of HZSM-5. HZSM-5 displays both strong and weak acid sites with a total amount of 0.39 mmol g^−1^ and TaS-1 exhibits a minor amount (~0.02 mmol g^−1^) of weak acid sites. Interestingly, TaAlS-1 shows only weak acid sites, but their concentration is considerably higher (0.18 mmol g^−1^) than that of TaS-1. Thus, the incorporation of pentavalent tantalum species in the MFI-zeolite can directly tune the framework acidity to optimise its catalytic properties.

### Catalytic tests

Methanol conversions were performed over a fixed-bed reactor under flow conditions at 400 °C (Table 1). HZSM-5 with varying Si/Al ratios (15–37:1) show full conversions of methanol and moderate propene selectivities of 7.7–31% (Entries 1–3). Meanwhile, considerable amounts of ethene are produced (up to 19%). This indicates that both alkene and aromatic cycles (in favour of the formation of propene and ethene, respectively) occur over HZSM-5^6,27^, resulting in low P/E ratios of 1.1–1.7. Moreover, excess alkanes are produced (entries 1–3, 23–70%) as hydrogen transfer can readily occur over the strong acid sites in HZSM-5^28^. The lack of Brønsted acid sites in TaS-1 leads to very low methanol conversion (5.1–7.5%) and propene selectivity (<1%) with dimethyl ether (DME) as the major product (entries 4 and 5). Similarly, the weak acid sites in NbAlS-1^22^ restrict the formation of carbon–carbon bonds and result in a high selectivity of DME (99%) at 92% conversion of methanol (Supplementary Table 1). Interestingly, TaAlS-1 samples show significantly enhanced catalytic performance (entries 6 and 7). TaAlS-1(0.013/0.027/1) shows a high propene selectivity of 51%, a P/E ratio of 8.3 and an excellent time-on-stream stability of over 50 h at 100% conversion of methanol (Fig. 2f). A slightly higher propene selectivity of 52% and P/E ratio of 11 were achieved over TaAlS-1(0.008/0.027/1) at a methanol conversion of 97%. These results compare favourably with state-of-the-art catalysts in MTO reactions (Fig. 1, Supplementary Table 1).

**Table 1:** Summary of the methanol conversion and product selectivities over different catalysts in MTO reactions^a^.

Entry	Catalyst	Conversion (%)	Selectivity (%)						C_3_H_6_/C_2_H_4_
			DME	C_2_H_4_	C_3_H_6_	C_4_H_8_	C_2_^0^–C_4_^0 b^	Others^c^	
1	HZSM-5(0.027/1)	100	0	19	31	18	23	8.6	1.7
2	HZSM-5(0.04/1)	100	0	12	14	5.2	57	11	1.2
3	HZSM-5(0.067/1)	100	0	7.0	7.7	2.8	70	12	1.1
4	TaS-1(0.008/1)	5.1	>99	<0.1	0.4	0	0	0	–
5	TaS-1(0.013/1)	7.5	>99	<0.1	0.7	0	0	0	–
6	TaAlS-1(0.008/0.027/1)	97	1.3	4.8	52	25	7.2	9.7	11
7	TaAlS-1(0.013/0.027/1)	100	0	6.1	51	28	6.9	8.1	8.3
8	TaAlS-1(0.013/0.02/1)	86	64	2.9	18	10	1.2	3.6	6.1
9	TaAlS-1(0.013/0.013/1)	20	93	<0.1	1.9	2.7	0.1	2.4	–
10	Ta_2_O_5_/HZSM-5(0.027/1)^d^	100	0	20	33	19	23	6.1	1.7

^a^Reaction conditions: catalyst, 2.0 g; reaction temperature, 400 °C; atmospheric pressure; weight hourly space velocity (WHSV), 0.16 h^−1^; time-on-stream = 2 h.

^b^C_2_^0^–C_4_^0^ refer to C_2_–C_4_-saturated hydrocarbons.

^c^Other products include C_5_ and higher hydrocarbons and aromatics.

^d^The ratio of Ta/Al/Si in powdered mixture of Ta_2_O_5_/HZSM-5(0.027/1) (4.3 wt%/95.7 wt%) is 0.013/0.027/1.

It has been widely reported that strong acid sites in zeolite catalysts favour the aromatic cycle to produce ethene, while the alkene cycle occurs on relatively weak acid sites to yield propene^27,29^. The incorporation of Ta(V) effectively moderates the strong Brønsted acid sites created by Al(III) sites in TaAlS-1 and thus hinders the aromatic cycle to promote the propene selectivity and P/E ratios and slows down the formation of coke. Reduction of Al(III) sites in TaAlS-1 decreases the acidity (Supplementary Table 5) and leads to the production of DME as the primary product (Table 1, entry 8). However, the distribution of propene among hydrocarbons produced over TaAlS-1(0.013/0.02/1) is 50.4% (Table 1, entry 8), which is similar to that obtained over TaAlS-1(0.013/0.027/1). This result suggests that over TaAlS-1(0.013/0.02/1), the conversion of DME to olefins is significantly hindered, while the transformation of ethene to higher olefins is affected to a less extent, further indicating that the formation of the first carbon–carbon is the limiting step. Further reduction of Al(III) sites restricts the formation of first carbon–carbon bonds and hence the following reactions, leading to the low selectivity of propene (Table 1, entry 9). Thus, a suitable amount of Al(III) sites is essential for the production of propene. The powdered mixture of Ta_2_O_5_ and HZSM-5 shows similar activity to that of HZSM-5, indicating that the cooperativity between Ta(V) and Brønsted acid sites within the microenvironment is vital (Table 1, entry 10). The time-on-stream test on TaAlS-1(0.013/0.027/1) at conversion of 94% showed that the conversion decreased rapidly (Supplementary Figs. 9–12). This is likely due to the promoted pathway of hydrogen transfer over Lewis and Brønsted acid sites in the presence of methanol, leading to rapid coke formation^28^. However, at full conversion, the activity remains intact for 54 h and the used catalyst shows only minor difference in the textual properties (Fig. 2f and Supplementary Table 2, Supplementary Figs. 13 and 14), demonstrating the excellent stability of this bifunctional zeolite. Overall, these results suggest that the strength, nature and distribution of the active sites are key parameters for optimising the MTO catalyst.

### Determination of adsorption domains for methanol

**Fig. 3:**
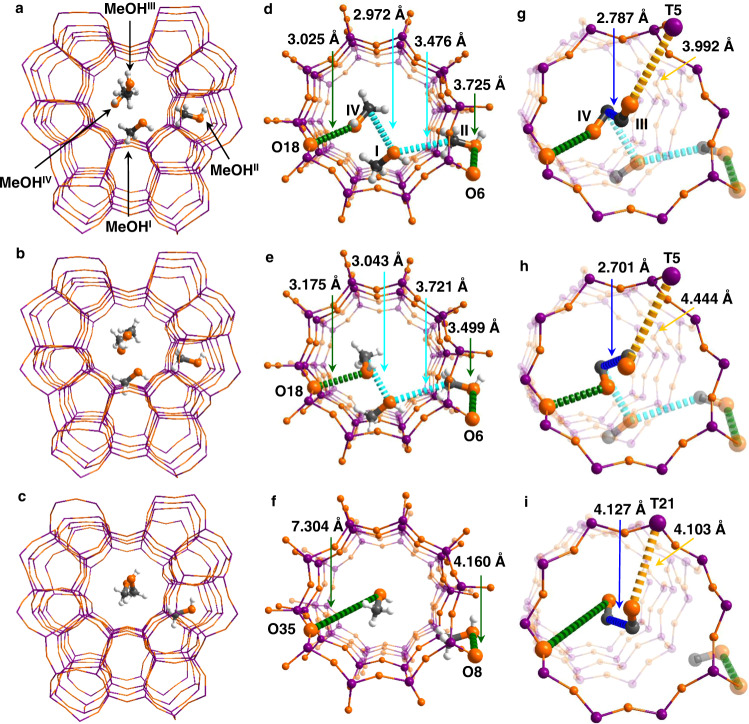
Views of crystal structures of MeOH-loaded TaAlS-1(0.013/0.027/1), HZSM-5(0.027/1) and TaS-1(0.013/1). All models were obtained from Rietveld refinements of SXPD data. Four distinct binding sites (I–IV) for MeOH have been observed in the straight and sinusoidal channels. **a** TaAlS-1(0.013/0.027/1)·8.6MeOH (Al_2.5_Ta_1.2_Si_92.3_O_192_·8.6CH_4_O), **b** HZSM-5(0.027/1)·12.3MeOH (Al_2.5_Si_93.5_O_192_·12.3CH_4_O). **c** TaS-1(0.013/1)·4.0MeOH (Ta_1.2_Si_94.8_O_192_·4.0CH_4_O). Only three binding sites were observed in TaS-1. The details of the host–guest binding are enlarged in **d**–**i**. Detailed views of MeOH^I,II,IV^ **d–f** and MeOH^III^ **g–i** in TaAlS-1(0.013/0.027/1), HZSM-5(0.027/1) and TaS-1(0.013/1), respectively. MeOH molecules and the functional sites involved in the cooperative binding are highlighted using an amplified ball-and-stick model (Ta/Al/Si, violet; C, grey; O, orange; H, white). The O···O(H) interactions and O···Ta interactions are highlighted in green and yellow, respectively. The C···O distances and C···C distances are highlighted in cyan and blue, respectively. Owing to the uncertainty on locations of protons, all hydrogen bonds in this report are described as the distance between the O_MeOH_ and the O_zeolite_ centres.

In situ synchrotron X-ray powder diffraction (SXPD) study (Supplementary Figs. 15–17, Supplementary Tables 6–13) determined four binding sites of methanol molecules (denoted as I–IV; Fig. 3) within the pores. Adsorbed methanol molecules show three distinct spatial orientations within the pores of TaAlS-1, HZSM-5 and TaS-1 (Fig. 3a–c, Supplementary Note 2 Interaction between methanol and Ta/Al/H sites). MeOH^II^ and MeOH^IV^ are located within the straight and sinusoidal channels of TaAlS-1, interacting with bridging O(H)-centres through their C–OH groups via hydrogen bonds [C–O ∙ ∙ ∙ O = 3.025, 3.725 Å, respectively] (Fig. 3d, Supplementary Fig. 17a). MeOH^I^ resides between MeOH^II^ and MeOH^IV^ [C^IV^ ∙ ∙ ∙ O^I^ = 2.972 Å, C^II^ ∙ ∙ ∙ O^I^ = 3.476 Å] and this assembles a {MeOH}_3_ trimer that favours the formation of TMO as the reaction intermediate^16^. The TMO-type configuration is also observed in HZSM-5 (Fig. 3e), but not in TaS-1 due to lack of Brønsted acid sites (Fig. 3f). Interestingly, a previous SXPD study on the adsorption of MeOH vapour in HZSM-5 has revealed that one MeOH molecule adsorbed on the Brønsted acid site [C-O ∙ ∙ ∙ O = 2.910 Å] and a second MeOH molecule resided nearby^30^. MeOH^III^ is located close to MeOH^IV^ in both TaAlS-1 and HZSM-5 [C^IV^ ∙ ∙ ∙ C^III^ = 2.787, 2.701 Å respectively] (Fig. 3g, h, Supplementary Fig. 17b), and it interacts with Ta(V) sites in TaAlS-1 [O^III^ ∙ ∙ ∙ T5 = 3.992 Å], which is potentially crucial for the activation of the C–O bond and thus to the formation of the first C–C bond. ^29^Si NMR, inelastic neutron scattering (INS) and DFT calculations^22,31,32^ demonstrate that TaAlS-1 (0.013/0.027/1) has an optimal site distribution (Supplementary Figs. 18–20, Supplementary Tables 14–17, and Supplementary Note 3 Distribution of Ta/Al/H sites) that promotes the adsorption of methanol in TaAlS-1 via a “TMO-type” mechanism, highly consistent with the SXPD analysis and the excellent catalytic performance. In TaS-1, MeOH^III^ hardly interacts with MeOH^IV^ [C^IV^ ∙ ∙ ∙ C^III^ = 4.127 Å] (Fig. 3i), again consistent with the catalysis result. Thus, the excellent catalytic activity of TaAlS-1 originates from (i) the highly confined adsorption of methanol over Brønsted acid sites to form TMO-type moieties in the pores; (ii) the efficient activation of C–O bonds in additional methanol molecules adsorbed over Ta(V) sites.

### Studies of the coordination environment of Ta(V) sites

**Fig. 4:**
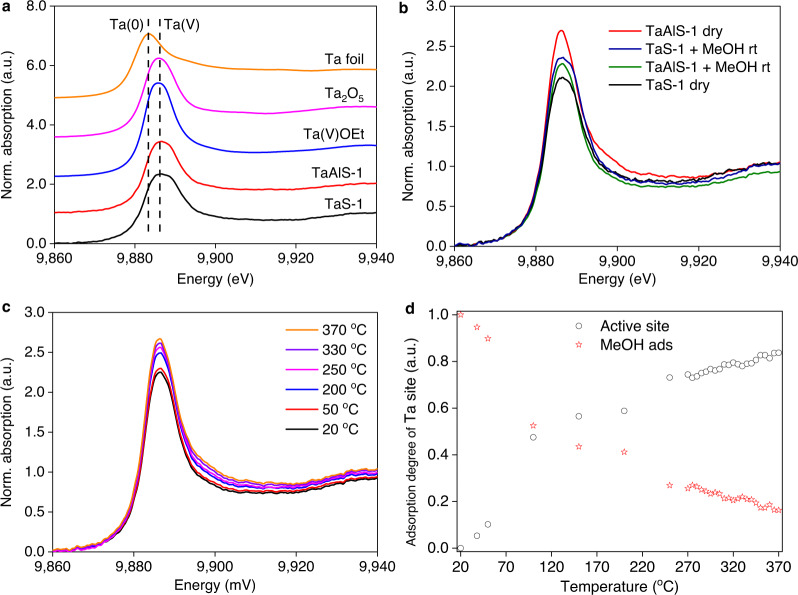
Ta L_3_-edge X-ray absorption spectroscopy (XAS) for TaAlS-1 zeolite and references. **a** XANES spectra at the Ta L_3_ edge of Ta foil, Ta(V) references and Ta-zeolites. **b** XANES spectra illustrating the influence of methanol adsorption at room temperature on Ta-zeolites. **c** *Operando* XANES spectra at the Ta L_3_ edge of TaAlS-1 zeolite during the conversion of methanol, only selected spectra shown. **d** Deconvolution of XANES via linear combination fitting to activated and adsorbed species.

The local environment of the active Ta(V) site was interrogated through Ta L3-edge X-ray absorption spectroscopy (XAS; Fig. 4a, b, Supplementary Figs. 21–26). The absorption edge peak positions of TaS-1 and TaAlS-1 (Fig. 4a) are consistent with Ta(V) oxidation state^33^, and the framework species are stable to the activation process employed prior to catalytic studies. Interestingly, dry TaAlS-1 shows an increase in the white line intensity (at 9886 eV originating from 2*p*_3/2_ → 5*d* transition^34^) relative to dry TaS-1 (Fig. 4b), representing a partial decrease in 5*d*-orbital filling. This arises from the charge imbalance resulting from framework Al(III)–O–Si species, consistent with the Py-IR analysis which shows increased Lewis acidity in TaAlS-1 relative to that of TaS-1 (Supplementary Fig. 7). This disparity in *d*-orbital occupancy of the Ta(V) sites influences the interaction with methanol. In TaAlS-1, adsorbed methanol interacts with the Ta(V) site by donating electrons to the Ta(V) *d*-orbital, as evidenced by the decrease in white line intensity (Fig. 4b), whereas in TaS-1 methanol accepts electrons resulting in the increase in white line intensity (Fig. 4b). This observation concurs with SXPD results, which clearly show different geometries of adsorbed methanol on TaAlS-1 and TaS-1 (Fig. 3). Thus, introduction of Ta(V) into the ZSM-5 framework provides new efficient adsorption sites for methanol.

Temperature-programmed *operando* XAS spectra (Fig. 4c and Supplementary Figs. 27 and 28) revealed again the high stability of the framework Ta(V) sites. No apparent change in oxidation state (edge position) or local environment (EXAFS) under reaction conditions is observed, although the white line intensity is shown to be proportional to temperature. Linear combination fitting of the XANES region reveals a decrease in the degree of methanol adsorption/interaction with increasing reaction temperature (Fig. 4d and Supplementary Fig. 29). Even at reaction temperature, adsorption of methanol at the Ta(V) site is still apparent, clearly demonstrating the unique interaction between methanol and the Ta(V) sites under reaction conditions (Fig. 4d and Supplementary Fig. 30).

### Studies of the reaction mechanism

**Fig. 5:**
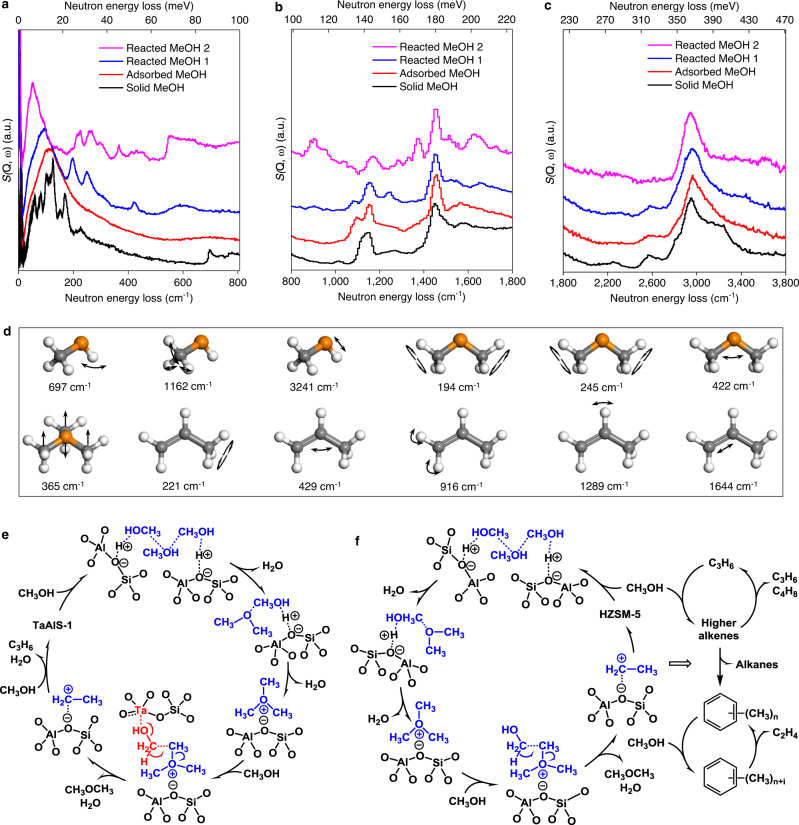
Inelastic neutron scattering (INS) spectra for TaAlS-1(0.013/0.027/1) on the adsorption and catalytic conversion of methanol and proposed reaction mechanism. All spectra shown here are difference spectra. Full spectra are shown in the SI. The INS spectra of reactant, intermediates and products were recorded and modelled via DFT calculations, allowing a full assignment of the spectral features. Where no error bars are visible these are smaller than the symbols used to represent the data points. Comparison of INS spectra obtained at TOSCA (**a**), at MAPS (**b**) at incident energy 250 meV over the momentum transfer range 0 Å^−1^ ≤ *Q* ≤ 9 Å^−1^ and, at MAPS (**c**) at incident energy 650 meV over the momentum transfer range 0 Å^−1^ ≤ *Q* ≤ 9 Å^−1^ for condensed solid methanol, adsorbed methanol, and reacted methanol on TaAlS-1(0.013/0.027/1). **d** Selected vibrational modes of methanol, DME, TMO and propene (C, grey; O, orange; H, white). Proposed reaction mechanism for the conversion of methanol in the induction period over **e** TaAlS-1 based on INS/SXPD experiments and **f** in the induction and dual-cycle period over HZSM-5 based on SXPD and literature reports^16,20^.

A combination of INS spectroscopy and DFT calculations^35,36^ were used to investigate the vibrational dynamics of methanol@TaAlS-1(0.013/0.027/1) *in operando* (Fig. 5a–d, Supplementary Figs. 31–44, Supplementary Tables 18–21; Supplementary Note 4 Inelastic neutron scattering). Comparison of INS spectra of adsorbed methanol and that of solid methanol show a number of differences. Peaks at low energy (below 150 cm^−1^), assigned to the lattice modes of solid MeOH, have become a continuum profile, suggesting that the MeOH molecules are strongly adsorbed in zeolite pores with heavily hindered motions. The C–O–H deformation (697, 778 cm^−1^) and the O–H stretch (3241 cm^−1^) modes of Me–OH groups are both greatly broadened (the weak feature at ~700 cm^−1^ the tail at 3400 cm^−1^, respectively) while the methyl deformation (1162, 1454 cm^−1^) and the C–H stretch modes (2955 cm^−1^) of the –CH_3_ group of MeOH are largely unchanged. Together, these suggest the binding of –OH group of MeOH in TaAlS-1 via the interaction with Ta(V) and Brønsted acid sites, consistent with SXPD and XAS results.

Catalytic conversion at 350 °C for 20 min of the adsorbed MeOH molecules on TaAlS-1 resulted in the spectra marked as “Reacted MeOH 1” in Fig. 5a–c. The new feature at 422 cm^−1^ (assigned as a C–O–C scissoring mode) together with two features at 194 and 245 cm^−1^ (assigned as methyl torsions), demonstrate the formation of DME. By comparing the INS spectra of solid and adsorbed DME, all these new INS peaks of reacted MeOH are consistent with that of the latter (Supplementary Figs. 45–47), indicating that the formed DME readily adsorbs on TaAlS-1. A further reaction was conducted at 370 °C for 90 min (spectrum marked as “Reacted MeOH 2” in Fig. 5a–c). The umbrella mode of TMO was clearly observed at 365 cm^−1^ (Fig. 5a, d, Supplementary Fig. 34, Supplementary Table 20), indicating that the adsorbed DME is attacked by another MeOH to form TMO and water; the librational modes of the latter are clearly seen at 500–700 cm^−1^ (Fig. 5a). Significantly, this represents the first vibrational spectroscopic evidence of the presence of TMO in MTO reactions and is in excellent agreement with the SXPD observation. These results, combined with previous observations by pulsed electron beam high pressure ion source mass spectrometry and NMR^16,37^, thus support the TMO-mechanism for the formation of the first carbon–carbon bond in MTO reactions.

Simultaneously, a new feature at 221 cm^−1^ (assigned to the methyl torsion of propene) appears and the C = C–C scissoring (429 cm^−1^), C = CH_2_ rocking (916 cm^−1^), =CH– rocking (1289 cm^−1^) and C = C stretching (1644 cm^−1^) modes of propene are all observed, demonstrating the formation of carbon–carbon bonds required for the product, propene. Additional new INS features at 1044, 1161, 1371, 1453 and 2972 cm^−1^ are fully consistent with the formation of propene (Supplementary Figs. 48–50).

A complete catalytic cycle based upon the structural, dynamic and modelling results for the MTO reaction over TaAlS-1 is established (Fig. 5e). In TaAlS-1, strong adsorption of methanol occurs on Ta(V) and Brønsted acid sites. The latter reacts with one weakly adsorbed methanol molecule to form DME, which reacts with a third methanol molecule on a Brønsted acid site to form TMO as a key intermediate. The TMO is then reacted with an activated methanol molecule on Ta(V) site to form the first carbon–carbon bond (Fig. 5e). This has been confirmed by temperature-programmed mass spectroscopy, where adsorbed TMO species within TaAlS-1 reacted with deuterated methanol, CD_3_OD, to yield partially deuterated propene, C_3_H_3_D_3_ (Supplementary Note 5 Temperature-programmed mass spectroscopy, Supplementary Figs. 51 and 52). By contrast, on HZSM-5, the formation of both intermediates and propene proceed solely on the Brønsted acid sites (Fig. 5f)^16^. After the induction period, the reaction over TaAlS-1 mainly proceeds via the alkene cycle to produce propene selectively, while on HZSM-5, the aromatic cycle dominates the reaction network to produce ethene as well as alkanes (Fig. 5f)^5,20,29^.

## Discussion

The sustainable production of propene will reduce our reliance on fossil fuels. Powerful drivers exist to optimise the product selectivity and catalytic stability in the complex MTO process to mitigate the projected global propene shortage. The new hetero-atomic zeolite TaAlS-1 integrates Ta(V) and Al(III) sites into the MFI-zeolite framework for the first time and shows a new distribution of active sites compared to conventional HZSM-5 materials. TaAlS-1 shows remarkable performance in converting methanol to propene. Ta(V) sites in the TaAlS-1 framework have two vital roles: (i) promoting the adsorption and activation of methanol, in cooperation with Brønsted acid sites, to drive the formation of TMO and sequentially carbon–carbon bonds; (ii) optimising the acidity of the zeolite so as to hinder the aromatic cycle and to restrict hydrogen transfer to minimise the formation of ethene, alkanes and coke. The product obtained from the flow-reactor contains primarily light olefins (85%) with only a small amount of alkanes (6.9%). Thus, huge energy savings can be potentially gained from the challenging alkene–alkane separation (e.g., the difference in boiling points of propene and propane is 5.6 °C) compared with the product that contains mostly alkanes over HZSM-5. Similarly, the high P/E ratio obtained from TaAlS-1 will also simplify the downstream separation of propene from ethene. TaAlS-1 is highly stable, and the synthesis of TaAlS-1 could readily be scaled up using the existing infrastructure for synthesising ZSM-5, further demonstrating its potential in these challenging industrial processes.

## Methods

### Catalyst preparation

HZSM-5 samples were obtained by calcining NH_4_-ZSM-5 at 550 °C under air flow for 14 h, and denoted as HZSM-5(Al/Si mole ratio). NH_4_-ZSM-5 samples were purchased from Alfa Aesar. TaAlS-1 and TaS-1 samples were prepared by hydrothermal reactions and denoted as TaAlS-1(Ta/Al/Si mole ratio) and TaS-1(Ta/Si mole ratio), respectively. In a typical synthesis, aluminium isopropoxide (99.99+%, Sigma Aldrich) was first dissolved in deionised water, into which tetrapropylammonium hydroxide solution (TPAOH, 1.0 M in H_2_O, Sigma Aldrich) as the structure-directing agent was added. After the mixture was stirred at room temperature for 2 h, tantalum ethoxide (99.999% metals basis, Alfa Aesar™) was added and the mixture was stirred for another 2 h. Then tetraethyl orthosilicate (98%, Sigma Aldrich) was added dropwise and the mixture was stirred for another 2 h, resulting in a gel with a chemical composition of 1Si:*x*Al:*y*Ta:0.25TPAOH:15H_2_O (*x* and *y* were determined by the target Al/Si and Ta/Si mole ratios, respectively). The gel was transferred into a 46-mL Teflon-lined stainless-steel autoclave, which was sealed and heated at 170 °C for 48 h. The solid products were centrifuged, washed with deionised water, dried overnight at 80 °C, and finally calcined at 550 °C under an air flow for 14 h. TaS-1 samples were synthesised by the same procedure but without the addition of aluminium isopropoxide. The typical yields of all zeolites in this study are 90–95%.

### Catalytic testing

Catalytic reactions were carried out in a stainless steel continuous-flow reactor (12.7 mm i.d.). Two grams of catalyst was pressed, crushed, and sorted into grains by 40–60 meshes, which were then activated at 550 °C for 3 h under a flow of nitrogen before the reaction. Methanol was injected into the nitrogen flow (N_2_/methanol mole ratio: 18.5) by syringe pump (Cole-Parmer) and passed through the reactor at the target temperature. The output liquid products were collected and analysed by gas chromatography (Agilent 7890B, equipped with an HP-5 column 30 m × 0.32 mm × 0.25 µm) and gas chromatography–mass spectrometry (GC–MS) (Agilent 6890N–Agilent 5973N, equipped with an HP-5MS column 30 m × 0.25 mm × 0.25 µm). The output gas products were collected and analysed by GC (Agilent Micro GC 490 equipped with a PoraPLOT U column, length 10 m) and GC–MS (Agilent 7890A-Agilent 5975C, equipped with an HP-PLOT/Q column 30 m × 0.53 mm × 40 µm). Methanol conversion was calculated by using Eq. (1):

1 $${\mathrm{MeOH}}\,{\mathrm{conversion}} = \left\{ {\left( {{\mathrm{MeOH}}_{{\mathrm{in}}}-{\mathrm{MeOH}}_{{\mathrm{out}}}} \right)/{\mathrm{MeOH}}_{{\mathrm{in}}}} \right\} \times 100\%$$

where MeOH_in_ and MeOH_out_ denote the moles of MeOH in the feed and exit, respectively. Product selectivity was calculated on a carbon basis using Eq. (2):

2 $${S}_{i} = \left\{ {\left( {{a}_{i} \times {n}_{i}} \right)/\left( {{\sum} {{a}_{i} \times {n}_{i}} } \right)} \right\} \times 100\%$$

where *a*_*i*_ and *n*_*i*_ denote the carbon number and number of moles of product *i*, respectively.

### X-ray absorption spectroscopy

Transmission Ta L_3_-edge turbo-X-ray absorption spectra were collected at the I20 beamline at the Diamond Light Source, employing non-monochromatic X-rays produced by a bent crystal polychromator and fast moving exit slits to enable a scan collection time of 4 s. Data collection spanned 200 eV before the edge to 1000 eV above it, with a step size of 0.3 eV; for the energy calibration a Ta foil reference sample was used. Data processing was carried out in the Demeter open source software package (version 0.9.26) with XAS spectra processing (normalisation and background subtractions) and linear combination fitting conducted within the Athena programmes. Reference spectrum of tantalum(V) ethoxide was collected in the liquid phase and Ta_2_O_5_ standards were collected after dilution in boron nitride. For temperature-programmed *operando* turbo-XAS experiments, 18 mg zeolite was loaded in a Harrick Praying Mantis cell. After zeolite activation at 550 °C and cooling the cell to room temperature, methanol was introduced via a Dreschel bottle with an N_2_ carrier gas (flow rate 1 mL min^−1^). Methanol was bubbled with a N_2_ flow into the cell at 1 mL min^−1^. The cell was heated at 10 °C min^−1^ from room temperature to 200 °C, then increased to 370 °C at 2 °C min^−1^. The XAS spectra were recorded and the exhaust was analysed by an on-line mass spectrometry.

### High-resolution SXPD and Rietveld refinement

SXPD data were collected on beamline I11 (Diamond Light Source, UK) using a wavelength of 0.82487(1) Å at room temperature. The zeolite powder was loaded into a 0.7 mm borosilicate glass capillary. High-resolution diffraction data were obtained from the samples using the multi-analyser crystal (MAC) detectors. The patterns were collected in the 2*θ* range 0–150° with 0.001° data step. Using the TOPAS software, the SXPD patterns were refined by the Rietveld method. The Thompson–Cox–Hastings pseudo-Voigt peak function^38^ was applied to describe the diffraction peaks. The scale factor and lattice parameters were allowed to refine for all diffraction patterns. The refined structural parameters include the fractional coordinates (*x*, *y*, *z*) and isotropic displacement factors (Beq) for all atoms, and the site occupancy factors (SOF) for all Ta, Al, Si species and methanol. The Beq values for all framework T sites and O sites are each refined with the same value. The quality of the Rietveld refinements has been assured by low goodness-of-fit (gof) factors, low weighted profile factors (Rwp) and well-fitted patterns with reasonable Beq that are within experimental errors. The crystallographic data (reference number CCDC-2022750–2022754) and refinement details are summarised in Supplementary Tables 6–13.

### Inelastic neutron scattering

INS spectra were recorded on the TOSCA^39^ and MAPS^40^ spectrometers at the ISIS Facility at the STFC Rutherford Appleton Laboratory (UK), as well as the VISION spectrometer at the Spallation Neutron Source, Oak Ridge National Laboratory (USA). Both TOSCA and VISION are indirect geometry crystal analyser instruments while MAPS is a direct-geometry inelastic spectrometer. The two types of instrument are complementary: TOSCA and VISION provide high-resolution spectra in the 0–2000 cm^−1^ range, MAPS provides access to the medium to high energy range (800–4000 cm^−1^) with good resolution. All the INS spectra were collected after the sample was cooled and stabilised at temperatures below 15 K.

The adsorption/reaction experiments were conducted at the TOSCA and MAPS beamlines. In a typical experiment, the catalyst (~11 g) was loaded into a flow-type Inconel cell^41^ that can also be used as a static cell with all valves closed. The sample was heated at 450 °C (5 °C min^−1^ ramp rate) under He for 3 h to remove any remaining trace water before the experiment. Then 30 mmol MeOH was injected into the cell at 25 °C. Before the data collection, the cell was flushed using dry He to remove weakly bound MeOH. The samples were cooled to <15 K before data collection. After each reaction, the cell was quenched in liquid nitrogen for INS collection to detect the presence of possible reaction intermediates. INS spectra of pure solid compounds for both starting material and reaction products were collected at 5 K. For measurements on MAPS, the samples were immediately and quickly transferred to an aluminium-can in a glovebox after reaction was quenched in liquid nitrogen. All MAPS spectra were recorded using the high-resolution A-chopper package at incident energies of 250 and 650 meV, with frequencies of 400 and 600 Hz, respectively. The MAPS spectra were integrated over a momentum transfer (*Q*) range of 0 ≤ *Q* ≤ 9 Å^−1^ to minimise overtone contributions present at higher *Q* values.

## Supplementary information

Supplementary Information

Peer Review File

## Data Availability

The raw time-of-flight TOSCA and MAPS spectra are available from: 10.5286/ISIS.E.RB1910009 and 10.5286/ISIS.E.RB2010255, respectively. The crystal structures are available free of charge from the Cambridge Crystallographic Data Centre under reference number CCDC-2022750–2022754. All relevant data are available from the corresponding authors, and/or are included with the manuscript.
