# Supplementary material for: Control of zeolite microenvironment for propene synthesis from methanol
Source: Nat Commun. 2021 Feb 5;12:822. doi: 10.1038/s41467-021-21062-1 (PMC7865006; doi:10.1038/s41467-021-21062-1)
Supplement: Supplementary file 1 — Supplementary Information [file 41467_2021_21062_MOESM1_ESM.pdf]

Supplementary Materials

Control of zeolite microenvironment for propene synthesis from methanol

Longfei Lin[1], Mengtian Fan[1], Alena M. Sheveleva[1,2], Xue Han[1], Zhimou Tang[3], Joseph H. Carter[1,4], Ivan da Silva[5], Christopher M. A. Parlett[4,6,7,8], Floriana Tuna[1,2], Eric J. L. McInnes[1,2], German Sastre[9], Svemir Rudić[5], Hamish Cavaye[5], Stewart F. Parker[5,8], Yongqiang Cheng[10], Luke L. Daemen[10], Anibal J. Ramirez-Cuesta[10], Martin P. Attfield[1], Yueming Liu[3], Chiu C. Tang[4], Buxing Han[11] and Sihai Yang[1]*

1. Department of Chemistry, University of Manchester, Manchester, M13 9PL (UK)
Email: Sihai.Yang@manchester.ac.uk
2. Photon Science Institute, University of Manchester, Manchester, M13 9PL (UK)
3. Shanghai Key Laboratory of Green Chemistry and Chemical Processes, School of Chemistry and Molecular Engineering, East China Normal University, Shanghai, 200062 (China)
4. Diamond Light Source, Harwell Science and Innovation Campus, Didcot, Oxfordshire, OX11 0DE (UK)
5. ISIS Facility, STFC Rutherford Appleton Laboratory, Chilton, Oxfordshire, OX11 0QX (UK)
6. Department of Chemical Engineering and Analytical Science, University of Manchester, Manchester, M13 9PL (UK)
7. University of Manchester at Harwell, Diamond Light Source, Harwell Campus, Didcot, Oxfordshire, OX11 0DE (UK)
8. UK Catalysis Hub, Research Complex at Harwell, Harwell Science and Innovation Campus, Didcot, Oxfordshire OX11 0FA (UK)
9. Instituto de Tecnologia Quimica, UPV-CSIC Universidad Politecnica de Valencia. Avenida Los Naranjos s/n, 46022 Valencia (Spain)
10. Neutron Scattering Division, Oak Ridge National Laboratory, Oak Ridge, TN 37831 (USA)
11. Beijing National Laboratory for Molecular Sciences, CAS Key Laboratory of Colloid, Interface and Chemical Thermodynamics, Institute of Chemistry, Chinese Academy of Science, Beijing, 100190 (China)

Table of Contents

Supplementary Methods **3**

Catalyst characterisation 3

DFT calculations and modelling of the INS spectra 4

Supplementary Notes (1-4) **6**

1. EPR spectroscopy 6
2. Interaction between methanol and Ta/Al/H sites 7
3. Distribution of Ta/Al/H sites 7
4. Inelastic neutron scattering 9
5. Temperature programmed mass spectroscopy 10

Supplementary Figure **11**

Supplementary Table **62**

Supplementary References **90**

Supplementary Methods

Catalyst characterisation

Powder X-ray diffraction (PXRD) patterns were recorded on a Philips X'pert X-ray diffractometer (40 kV and 30 mA) using Cu $K\alpha_1$ radiation (λ = 1.5406 Å). N_2 adsorption was carried out at 77 K on a Micromeritics 3Flex instrument after activating the samples for 10 h under dynamic vacuum at 623 K. The crystal morphology and size were measured by scanning electron microscopy (SEM) on a Quanta FEG 650 microscope. The ratios of Ta/Al/Si in the sample were quantified by EDX using multiple regions over a sample on a Bruker XTrace instrument. Thermogravimetric analysis was carried out with a SDTQ600 TA instrument. Samples were heated from room temperature to 800 °C at a rate of 10 °C min^{-1} under an air flow at 100 mL min^{-1}. Attenuated total internal reflection Flourier transformed infrared (ATR-FTIR) spectra were collected with Nicolet iS5 spectrometer. The acidity was measured by temperature-programmed desorption of ammonia (NH_3-TPD) with a Quantachrome Autosorb-1 equipped with a thermal conductivity detector (TCD). Typically, 100 mg of sample was pre-treated in a helium stream (30 mL min^{-1}) at 600 °C for 2 h. The adsorption of NH_3 was carried out at 50 °C for 1 h. The catalyst was flushed with helium at 100 °C for 2 h to remove physisorbed NH_3 from the catalyst surface. The TPD profile was recorded at a heating rate of 10 °C min^{-1} from 100 to 600 °C. Used catalysts were calcined at 600 °C for 6 h in air flow prior to the NH_3-TPD experiments. The Brønsted and Lewis acid sites of the samples were investigated by FT-IR of adsorbed pyridine in an *in situ* cell with CaF_2 windows. Wafers with a weight of 25 mg and radius of 6.5 mm were degassed for 1 h under vacuum at 600 °C. Then pyridine was admitted, and after equilibration, the samples were outgassed for 0.5 h at increasing temperatures (150, 200, 250, 350 and 450 °C). The spectra were recorded on a Nicolet iS50 FT-IR spectrometer. Solid-state ^{29}Si NMR spectra were recorded with a frequency of 79.46 MHz, a spinning rate of 10.0 kHz, and a recycling delay of 10 s. Tetramethylsilane was used as the reference for the chemical shift.

For the measurement of electron paramagnetic resonance (EPR) spectroscopy, the sample was placed in a 2.8 mm o.d. quartz tube and connected to a vacuum line. Prior to irradiation, zeolite materials were activated by pumping at 10^{-5} Torr for 12 h at 150 ˚C and then flame sealed under vacuum. The samples were exposed to γ-irradiation from a ^{60}Co source at 77 K to a total dose of 4.1 MRad at a dose rate of 0.48 MRad h^{-1}. CW EPR measurements were carried out at the X-band frequency (9.4 GHz) using a Bruker EMX spectrometer equipped with an Oxford Instruments temperature control system at 77 K. EPR spectra were

detected with modulation amplitudes of 0.2 and 1 mT, and microwave powers varied in the range ~0.7-70 mW. For the data presented here, 7 to 70 mW was chosen to provide optimum signal intensity. Pulsed EPR measurements of samples after irradiation were performed at the X-band frequency (*ca.* 9.7 GHz) on a Bruker Elexsys E580 spectrometer. The microwave frequency was measured with a built-in digital counter and the magnetic field was calibrated using the microwave pulse the sequence ($\pi/2 - \tau - \pi - \tau$ – echo) with $\pi/2$ and π pulse length of 16 and 32 ns. The interpulse delay τ was 150 ns. HYSCORE[1] spectra were measured at X-band using a pulse sequence ($\pi/2 - \tau - \pi/2 - T_1 - \pi - T_2 - \pi/2 - \tau$ – echo) with $\pi/2$ = 16 ns and π = 26 ns, and fixed τ = 136 ns or τ = 200 ns; The initial values of T_1 and T_2 were 100 ns. The (128×128) HYSCORE data array was recorded with the time increment of 16 ns, and then, data were Fourier transformed (FT) to produce 2D spectra. Theoretical modelling of all EPR data was performed using EasySpin toolbox (Version 5.2.28) for Matlab.

Synthesis of TMO@TaAlS-1 and temperature-programmed mass spectroscopy (TP-MS)

TMO@TaAlS-1 was prepared using a method reported in the literature[2]. TaAlS-1(0.013/0.027/1) (1.5 g) was suspended in pre-cooled, dry nitromethane (9.0 g) and to it was added trimethyloxonium tetrafluoroborate (0.9 g) in the same solvent (3 g; -20 °C). All operations were carried out using anhydrous Schlenkware. After vigorous manual shaking, this suspension was set aside (-20°C; 1 h), filtered, washed with a small portion of pre-cooled nitromethane, then dried in vacuo at room temperature for 3 hours.

The experiments of TP-MS were carried out in a stainless-steel continuous-flow reactor (6.35 mm i.d.). 0.33 g of TMO@TaAlS-1(0.013/0.027/1) or TaAlS-1(0.013/0.027/1) was loaded in the reactor, followed by injection of 0.3 mL of CD_3OD or CH_3OH into the reactor. The reactor temperature was increased from room temperature to 400 °C at 10 °C min^{-1} under a flow of helium at 100 mL min^{-1}. The efflux was monitored by online mass spectroscopy.

DFT calculations and modelling of the INS spectra

Simulation of INS spectra of bare TaAlS-1 zeolite and TMO species. Periodic density functional theory (periodic-DFT) calculations were carried out using the plane wave pseudopotential method as implemented in the CASTEP code[3,4]. Exchange and correlation were approximated using the Perdew-Burke-Ernzerhof (PBE) functional[5]. Ultra-soft pseudopotentials were employed to account for the effects of core electrons.

The Tkatchenko-Scheffler dispersion correction[6] was used for van der Waals interactions. The Energy cut-off for the plane-wave basis set was 380 eV. Phonon frequencies were obtained by diagonalisation of dynamical matrices computed using the finite displacement method. The atomic displacements in each mode that are part of the CASTEP output, enable visualization of the modes to aid assignments and are also all that is required to generate the INS spectrum using the program OCLIMAX[7,8]. DFT calculations of the INS spectrum for single trimethyloxonium tetrafluoroborate molecule was carried out, which were used to identify the modes of vibrational features in the experimental INS spectrum. In addition, DFT calculations of the INS spectra for bare TaAlS-1 were performed to investigate the distribution of Ta/Al/H sites. Due to the large number of configurations involved in these simulations, the CP2K code[9] based on the mixed Gaussian and plane-wave scheme[10] and the Quickstep module[11] were used. The calculation used the molecularly optimised Double-Zeta-Valence plus Polarization (DZVP) basis set[12], Goedecker-Teter-Hutter pseudopotentials[13], and the PBE exchange correlation functional[5]. The plane-wave energy cutoff was 400 Ry. The DFT-D3 level correction for dispersion interactions, as implemented by Grimme *et al*[14], was applied.

Simulation of the INS spectrum of solid methanol. Periodic-DFT calculations were carried out using CASTEP (version 7.03) with the PBE functional. Norm conserving pseudopotentials with a plane wave cut-off of 1000 eV were employed, with a Monkhorst-Pack grid of 10×10×5 (75 *k*-points). After geometry optimisation, the residual forces were converged to better than |0.004| eV $Å^{-1}$. Phonon frequencies were obtained by diagonalization of dynamical matrices computed using density-functional perturbation theory[15]. In addition to the calculation of transition energies and intensities at zero wavevector, phonon dispersion was also calculated along high symmetry directions throughout the Brillouin zone. For this purpose, dynamical matrices were computed on a regular grid of wavevectors throughout the Brillouin zone and Fourier interpolation was used to extend the computed grid to the desired fine set of points along the high-symmetry paths[16]. The INS spectrum was calculated from the atomic displacements in each mode using the program aCLIMAX[8].

Simulation of the INS spectra of dimethyl ether and propene. Optimised geometry and vibrational frequencies of the lowest energy conformer of each molecule were calculated at the B3LYP/6-311++G(d,p) level of theory with the help of Gaussian suite of programs. INS spectra were obtained from calculated

eigenvectors and eigenenergies using the aCLIMAX programme[8], and were compared with the experimental data.

Supplementary Notes

Supplementary Note 1 EPR spectroscopy

The existence of Ta-O$^•$-Si defects after γ-irradiation was previously proposed in a Ta-modified MCM-41 zeolite[17] but the hyperfine structure associated with ^{181}Ta has not been resolved for that material. In the present work, γ-irradiated TaS-1 and TaAlS-1 samples show more detailed features. The spectra were simulated taking into account the hyperfine structure due to interaction of the electron with ^{181}Ta nuclei (I = 7/2; 99.98% natural abundance) and the considerable quadrupole interaction, which is expected to be observed in the continuous wave (CW) EPR spectrum due to the large quadrupole moment of Ta (Fig. 2e, Supplementary Fig. 6 and Supplementary Table 3). The average g-value in our work is in good agreement with literature data for M-O$^•$-Si defects[17,18]. For HZSM-5, EPR spectroscopy reveals interaction of an electron hole with Al(III) within the zeolite structure and the g and A parameters obtained are similar to those reported by Wichterlová *et al.*[18]. For consistency, EPR spectra of samples TaS-1, TaAlS-1 and HZSM-5 were simulated by superposition of the signals for M-O$^•$-Si (M=Ta or Al) and Si-O$^•$-Si defects, and the resulted simulation parameters are presented in Supplementary Table 3.

EPR-active defect sites induced by γ-irradiation in HZSM-5, TaAlS-1 and TaS-1 were further analysed by hyperfine sub-level correlation (HYSCORE) spectoscopy[19]. Experimental and simulated HYSCORE data are shown in Supplementary Fig. 5 and Supplementary Table 4. All data were collected at the same field orientation: 334 mT (9.4 GHz)/346 mT (9.735 GHz). HZSM-5 demonstrates off diagonal cross peaks in the (-,+) quadrant due to hyperfine (HF) coupling with ^{27}Al (A-tensor = [19.0 19.0 21.0] MHz) (magenta color in simulations in Supplementary Fig. 5) as confirmed in the CW spectum of HZSM-5. Additionally, we observe off diagonal cross peaks due to HF coupling with ^{29}Si (cyan colour in simulations in Supplementary Fig. 5) which are unresolved in the CW spectrum of the HZSM-5 sample. In the (+,+) quadrant, we observe peaks correspoding centered at the ^{29}Si larmor frequency. TaAlS-1 clearly demonstrates the presence of the same characreristic off diagonal cross peaks for ^{27}Al with a slight deviation in HFI values from those previously observed in HZSM-5 (A-tensor = [18.5 18.5 21.0] MHz). The latter confirms the presence of Al-O$^•$-Si sites in the TaAlS-1. The appearance of the ^{27}Al larmor frequecy diagonal peak in the (+,+) quadrant supports this analysis. Off diagonal cross peaks due to HF interaction with ^{29}Si

observed in the (-,+) quadrant deviates from those observed for HZSM-5 and therefore could refer to Ta-O$^{\bullet}$-Si sites, which is expected to display a stronger signals in Pulsed EPR due to longer relaxation times for such defects.

Supplementary Note 2 Interaction between methanol and Ta/Al/H sites

Synchrotron XPD has been successfully applied to study the interaction between guest molecules and porous materials, such as the binding of hydrocarbons in porous metal-organic frameworks[20], binding of γ-valerolactone in Zn/ZSM-5[21] and the binding of pyridine in HZSM-5[22]. In this study, over 3200 *hkl* reflections were used for the structural refinement, which allowed for extensive structural variables to be refined in a satisfactory manner. It is almost impossible to distinguish between Al and Si (Z=13 and 14, respectively) sites using X-rays diffraction. Although Ta (Z=73) is heavier, the low occupancy of Ta precludes the determination of its precise location in the framework. Recently, the distribution of active sites in H-ZSM-5[22] and Zn/ZSM-5[21] has been successfully determined by examining the intermolecular distances and angles between guest molecules and framework sites. For example, protonic acid sites of H-ZSM-5 have been located by examining the atomic distances and angles between pyridine and framework atoms[22]. Methanol can be adsorbed on Brønsted acid sites upon interaction between AlO(H)Si and the OH group of methanol[23], while it can also be adsorbed on Ta(V) sites *via* electron donation to the d-band of a Ta(V) site from the OH group of methanol (Fig. 4b). Thus, the distribution of active sites in TaAlS-1 has been revealed by a detailed examination of the MeOH-TaAlS-1 binding distances, particularly that between O_{MeOH} and $O_{zeolites}$ and between O_{MeOH} and T sites (see Supplementary Tables 12,13). Importantly, the distances between O_{MeOHII} and $O6_{zeolites}$, and that between O_{MeOHIV} and $O18_{zeolite}$ are shortest in both MeOH-adsorbed TaAlS-1 and HZSM-5, suggesting that protonic acid sites are located at O6-T2 and O18-T9 centres. Similarly, the distances between $O_{MeOHIII}$ and T5, and between $O_{MeOHIII}$ and T21 are shortest in MeOH-adsorbed TaAlS-1 and TaS-1, respectively, indicating that Ta(V) sites are likely located at T5 and T21 positions (Supplementary Tables 12,13).

Supplementary Note 3 Distribution of Ta/Al/H sites

In order to further study the distribution of Ta/Al/H sites, ^{29}Si NMR, inelastic neutron scattering (INS) and DFT calculations were employed. The ^{29}Si NMR spectra of TaAlS-1(0.013/0.027/1) and HZSM-5(0.027/1)

show notable differences in the range of -108 to -100 ppm (Supplementary Fig. 18a). The spectra were deconvoluted by fitting to a sum of Guassian and Lorentzian line shapes. The assignments and peak areas are listed in Supplementary Table 14. The (Al+Ta)/Si ratios calculated from the ^{29}Si NMR data are consistent with that obtained from EDX. The peak at -98 ppm is absent in the spectrum of HZSM-5(0.027/1), indicating the absence of $Si(OSi)_2(OAl)_2$ species (Supplementary Fig. 18b), consistent with previous reports[24,25]. The intensity of the peak at -108 ppm [assigned to $Si(OSi)_3(OAl/Ta)$] in the spectrum of TaAlS-1(0.013/0.027/1) is higher than that in HZSM-5(0.027/1), indicating the successful incorporating of Ta(V) sites into the framework but not adjacent to Al sites (*i.e.*, no “T pairs” sites), which is consistent with the SXPD result (Fig 3g). The population of the different types of Si (Q^n) of Ta/Al/H sites displayed in Fig. 3g were calculated by zeoTsites software[26] (Supplementary Table 15). The $Si(OSi)_3(OAl/Ta)$ is calculated to account for 13% (Supplementary Table 15), which matches the experimental observation (14%, Supplementary Table 14).

Recently, the distribution of protons in LTA zeolite has been studied using periodic DFT calculations and INS[27,28]. The distribution of Ta/Al/H sites in TaAlS-1(0.013/0.027/1) has been further investigated by combining INS and DFT calculations. Based on ^{29}Si NMR results, only “isolated single” T sites are considered. From the Rietveld refinement of SXPD date (Fig. 3g), one Ta and two Al sites are positioned at T5, T2 and T9, respectively, for the calculation of the corresponding INS spectra (Supplementary Table 16). The H sites have the same probability to appear near all four O around Al, and are thus attached to O6, O1, O2, O13, O18, O25, O8 and O9, respectively (Ta/Al/H_1-16, Supplementary Table 16). For comparison, three other sites are also considered (Ta/Al/H_17-19, Supplementary Table 16). For Ta/Al/H_17, Ta and Al^{I} are positioned at T5 and T2, respectively, same as for Ta/Al/H_1, but moving Al^{II} to T11. For Ta/Al/H_18, Ta and Al^{II} are positioned at T5 and T9, respectively, same as for Ta/Al/H_1, but moving Al^{I} to T4. For Ta/Al/H_19, Al^{I} and Al^{II} are positioned at T2 and T9, respectively, same as for Ta/Al/H_1, but moving Ta to T8. All nineteen Ta/Al/H sites are simulated independently by DFT and the calculated INS spectra are compared with the experimental data of bare TaAlS-1(0.013/0.027/1) (Supplementary Fig. 19). As there is an uncertainty on the position of H, each calculated INS spectra of Ta/Al/H_1-16 is given a same weight to assemble the combined spectrum (combination 1 in Supplementary Fig. 20). The spectrum of combination 2 is produced by combining spectra Ta/Al/H_17-19 with a same weight. All peaks in combination 1 are consistent with those in the experimental data, particularly the peaks assigned to -OH in-plane bending and Si-O stretching at 1096 and 1218 cm^{-1}, respectively (Supplementary Fig. 20, Supplementary Table 17). The

intensities of peaks below 497 cm^{-1} (assigned to relaxation of zeolite framework and -OH out-of-plane bending in combination 1) are lower than that of the experimental data because only one unit cell and a simplified model were used and external SiOH groups are not considered in the simulation. Combination 2 is produced with other Ta/Al/H sites (Supplementary Fig.20), and a number of peaks (53, 148, 267 and 304 cm^{-1}) are absent compared with the experimental data. These results does not mean the TaAlS-1 contains various compounds (with the same Ta/Al/H sites used here for modelling), but demonstrating TaAlS-1 (0.013/0.027/1) has an optimal site distribution (Fig. 3g) that promotes the adsorption of methanol in TaAlS-1 *via* a "TMO-type" mechanism.

Supplementary Note 4 Inelastic neutron scattering

Direct visualisation of the interaction between adsorbed methanol and the active sites is crucial to understand the molecular details of adsorption, activation and conversion into propene. INS is a powerful neutron spectroscopy technique to investigate the dynamics (particularly for the deformational and conformational modes) of methanol. It has several advantages:

- INS spectroscopy is sensitive to the vibrations of hydrogen atoms, and hydrogen is ten times more visible than other elements due to its high neutron scattering cross-section.
- The technique is not subject to any optical selection rules. All vibrations are active and, in principle, measurable.
- INS observations are not restricted to the centre of the Brillouin zone (gamma point) as is the case for optical techniques.
- INS spectra can be readily and accurately modelled: the intensities are proportional to the concentration of elements in the sample and their cross-sections, and the measured INS intensities relate straightforwardly to the associated displacements of the scattering atom. Treatment of background correction is also straightforward.
- Neutrons penetrate deeply into materials and pass readily through the walls of metal containers making neutrons ideal to measure bulk properties of this material (in this case for 11 g catalyst).
- INS spectrometers cover the whole range of the molecular vibrational spectrum, 0-500 meV (0-4000 cm^{-1}).

- INS data can be collected at low temperature (< 15 K in this case), where the thermal motion of the catalyst, the adsorbed methanol and the reacted intermediate molecules can be significantly reduced.
- Peak intensities in INS can be wholly described by the neutron scattering cross-sections and absolute quantities of the atoms involved. This means that predicting INS spectra is often a much more reliable process than for other techniques. For small, isolated molecular species or materials with well-characterised crystal structures, agreement between predicted and experimental spectra can be very good, thus leading to the full assignment of the features in the spectra.

Supplementary Note 5 Temperature programmed mass spectroscopy

A series of experiments based upon TP-MS have been conducted to study the reactivity of TMO ($C_3H_9O^+$) for the formation of olefins within the zeolites (Supplementary Figs. 51,52). Firstly, TMO was dosed to TaAlS-1 to afford TMO@TaAlS-1, and part of the sample was then loaded with CH_3OH or CD_3OD. The systems were heated to 400 °C at 10 °C min^{-1} under a helium flow and the efflux monitored by online MS. Heating TMO@TaAlS-1+CD_3OD at 292 °C yielded a series of MS signals of m/z at 42, 45, 46 and 48 (Supplementary Fig. 52a-b). The signal of m/z 45 corresponds to $C_3H_3D_3$, which was produced *via* the reaction between adsorbed TMO and CD_3OD species (Supplementary Fig. 51). It is worth noting that the signal of m/z 45 is significantly decreased or indeed absent in the reaction of TMO@TaAlS-1 or TaAlS-1+CD_3OD at 292 °C (Supplementary Fig. 52c,d), thus confirming that the reaction between adsorbed TMO and CD_3OD species plays a key role in the formation of C-C bonds in propene. C_3D_6 (m/z 48) was produced from CD_3OD over TaAlS-1 (Supplementary Fig. 52b,d). The signal of m/z 46 could be originated from fragments of C_3D_6 or $C_3H_2D_4$ (Supplementary Fig. 51). Moreover, the formation of CH_3OCH_3 or CD_3OCD_3 peak at around 270 °C (Supplementary Figs. 52b,d,e). The formation of propene in the system of TaAlS-1+CD_3OD (Supplementary Fig. 52d) peaks at 350 °C, whereas it peaks at 292 °C in both systems of TMO@TaAlS-1+CD_3OD (Supplementary Fig. 52b) and TMO@TaAlS-1+CH_3OH (Supplementary Fig. 52e). Thus, this result demonstrates that the presence of TMO in the pore can promote the formation of olefins.

Supplementary Figure

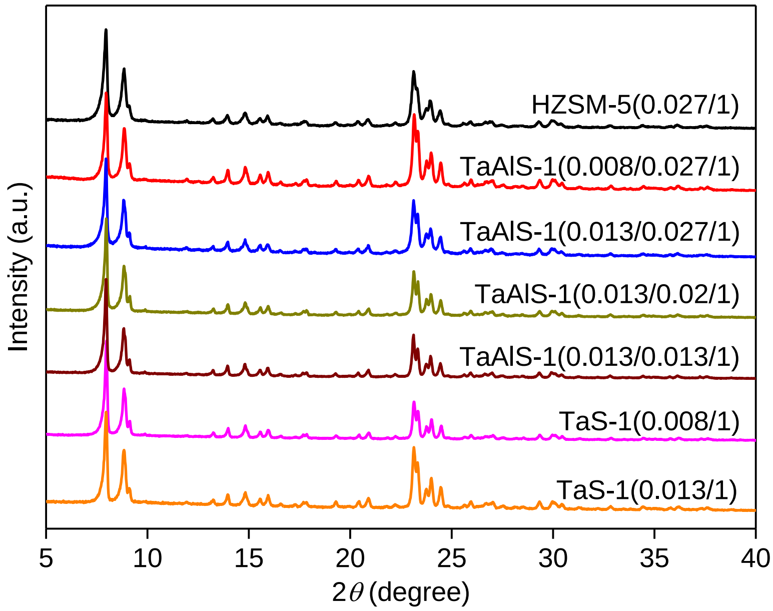

Supplementary Fig. 1. Comparison of PXRD patterns of HZSM-5, TaAlS-1 and TaS-1 (λ = 1.5406 Å).

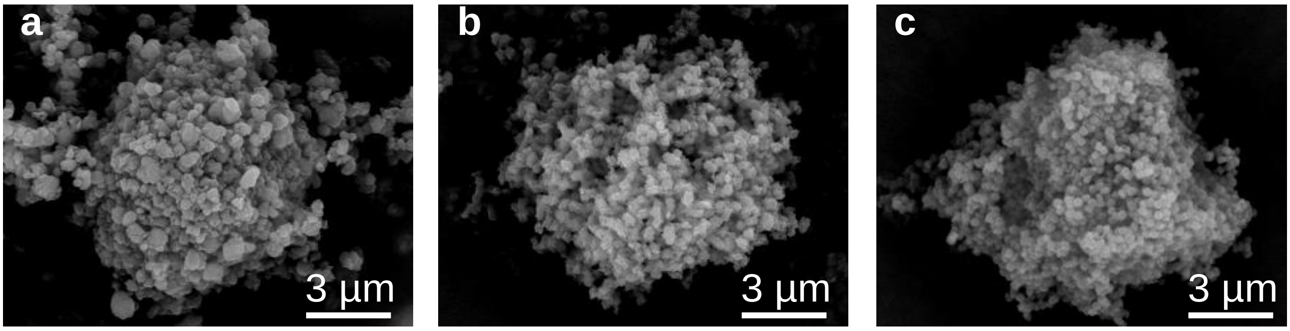

Supplementary Fig. 2. SEM images of (a) HZSM-5(0.027/1), (b) TaAlS-1 (0.013/0.027/1) and (c) TaS-1(0.013/1).

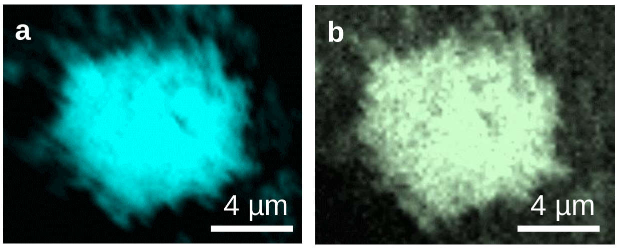

Supplementary Fig. 3. EDX map of Si K_α (**a**) and Al K_α (**b**) in TaAlS-1(0.013/0.027/1).

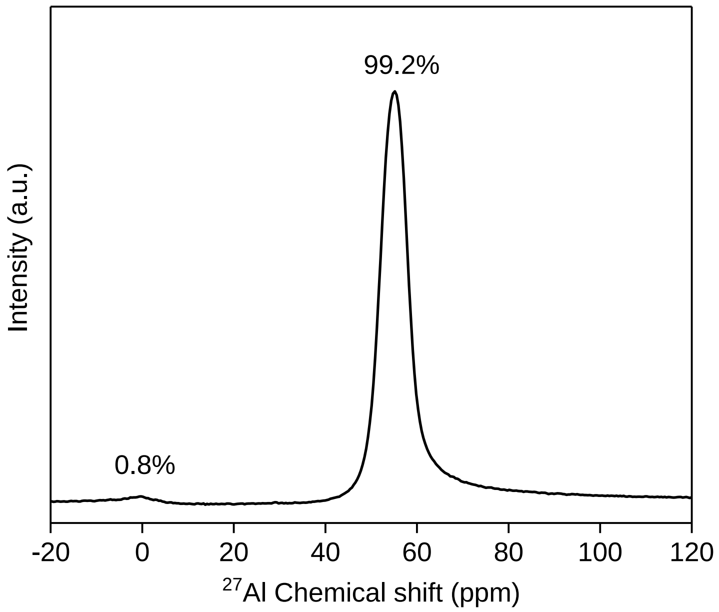

Supplementary Fig. 4. ^{27}Al NMR spectrum of TaAlS-1(0.013/0.027/1).

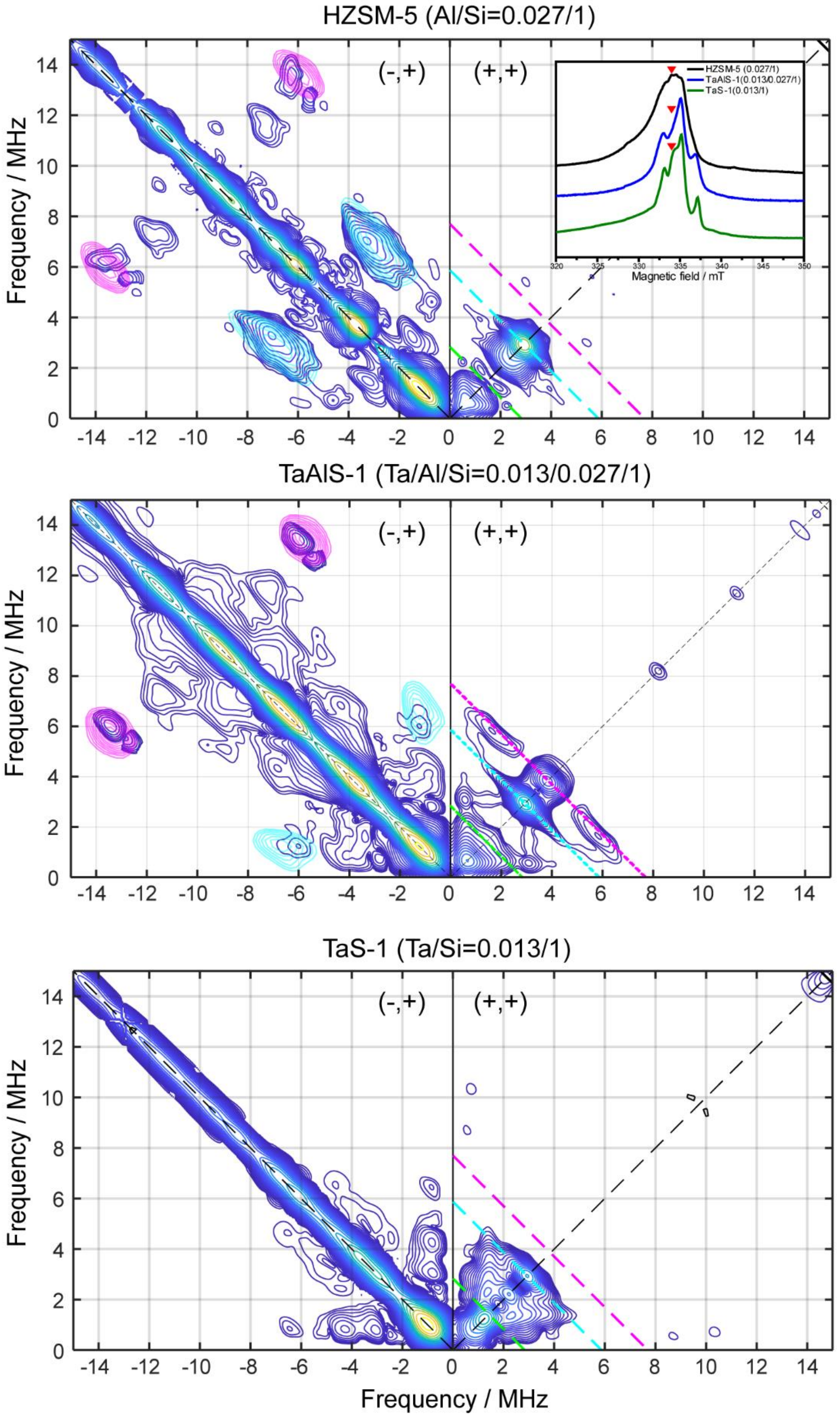

Supplementary 5. X-band (9.735 GHz) HYSCORE spectrum recorded at 6.5 K and at 346 mT (9.735 GHz)/334 mT(9.4 GHz) field position with τ value of 200 ns (in ED insert graph field is normalised for frequency value of 9.4 GHz in order to match with the CW data). Off diagonal dashed lines correspond to the position of the Larmor frequency of different nuclei: magenta dashed line - ^{27}Al; cyan dashed line - ^{29}Si; green dashed line - ^{181}Ta. Simulated cross-peaks are shown in magenta. The parameters used for the simulation are presented in Supplementary Table 4.

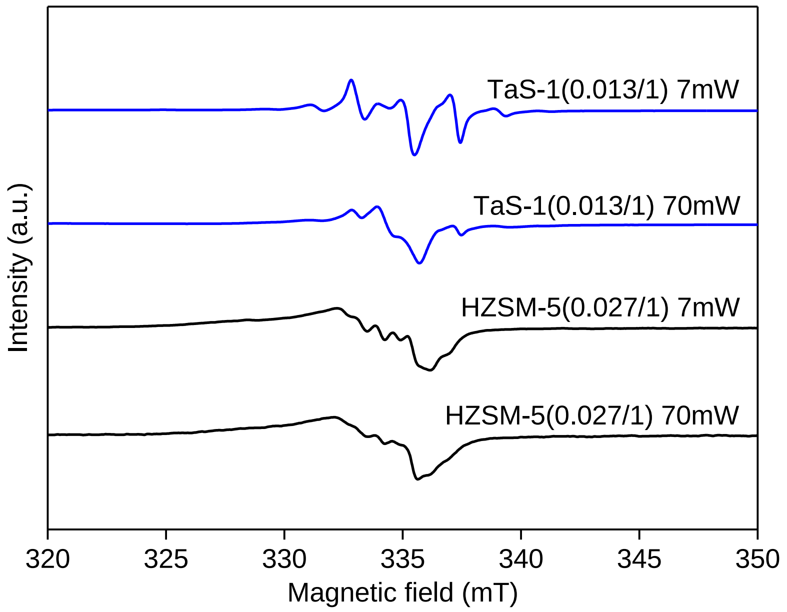

Supplementary Fig. 6. X-band (9.4 GHz) CW EPR spectra of TaS-1 and HZSM-5 activated by γ-irradiation at 77 K, recorded with microwave powers of 7 and 70 mW.

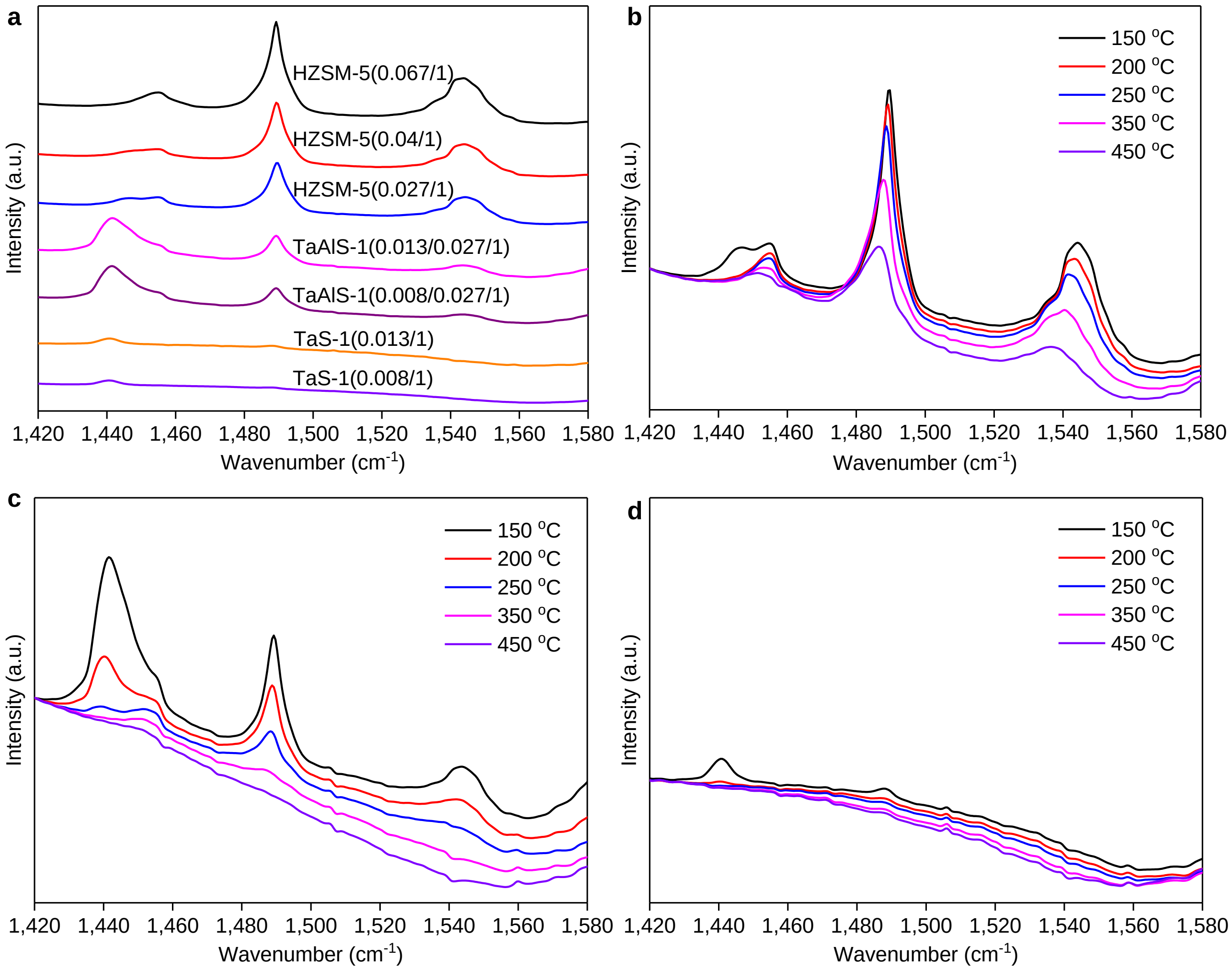

Supplementary Fig. 7. Characterisation of the acidity of zeolites by pyridine-IR. (**a**) Py-IR spectra after vacuum treatment at 150 °C, Py-IR spectra of (**b**) HZSM-5(0.027/1), (**c**) TaAlS-1(0.013/0.027/1) and (**d**) TaS-1(0.013/1) after vacuum treatment at variable temperatures. The peaks at 1454 and 1545 cm^{-1} are assigned to the coordinatively bound pyridine molecules on Lewis acid sites and pyridinium ion on Brønsted acid sites, respectively[29]. Both the coordinatively bound pyridine and pyridinium ion have the vibration peak at 1490 cm^{-1} [29]. The Lewis acid band is centred at 1454 cm^{-1} in the spectra of HZSM-5, while it shifts to 1447 cm^{-1} in the spectra of Ta-doped zeolite. The decrease of frequency of the band is related to the reduced stability of the pyridine complex[29], which is verified by the desorption experiments. **b**, **c** and **d** show the Py-IR spectra of HZSM-5(0.027/1), TaAlS-1(0.013/0.027/1) and TaS-1(0.013/1), respectively, after desorption of pyridine at variable temperatures. With the increase of temperature, the peak intensities related to both Lewis and Brønsted acid sites of HZSM-5 decrease slowly. Both peaks are visible even after the vacuum treatment at 450 °C (**b**). However, the intensities of these two peaks in the spectra of TaAl-1 and TaS-1 decreased dramatically upon increasing temperature and all disappeared at 450 °C (**c** and **d**). This result indicates that the TaAlS-1 samples have only weak Lewis and weak Brønsted acid sites, entirely consistent with the NH_3-TPD results.

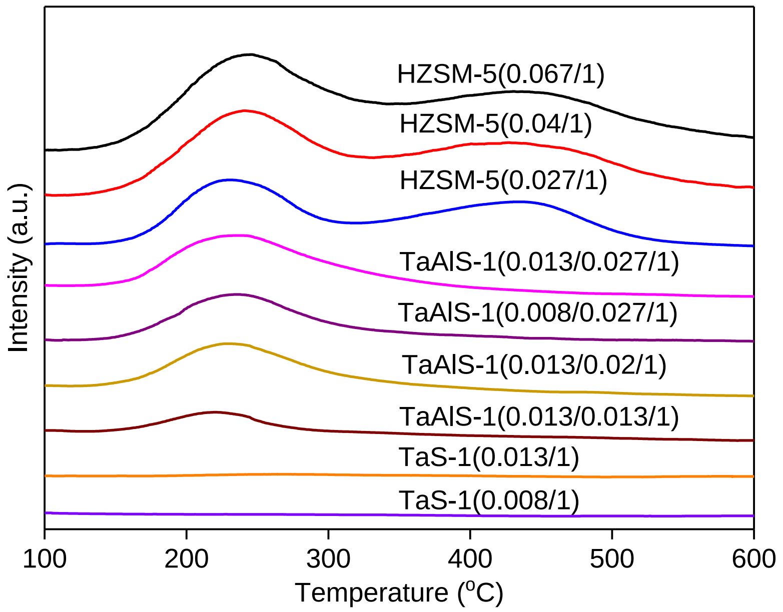

Supplementary Fig. 8. Characterisation of the acidity of zeolites by NH_3-TPD. Two peaks at 240 and 435 °C correspond to NH_3 eluted from the weak and strong acid sites, respectively[30].

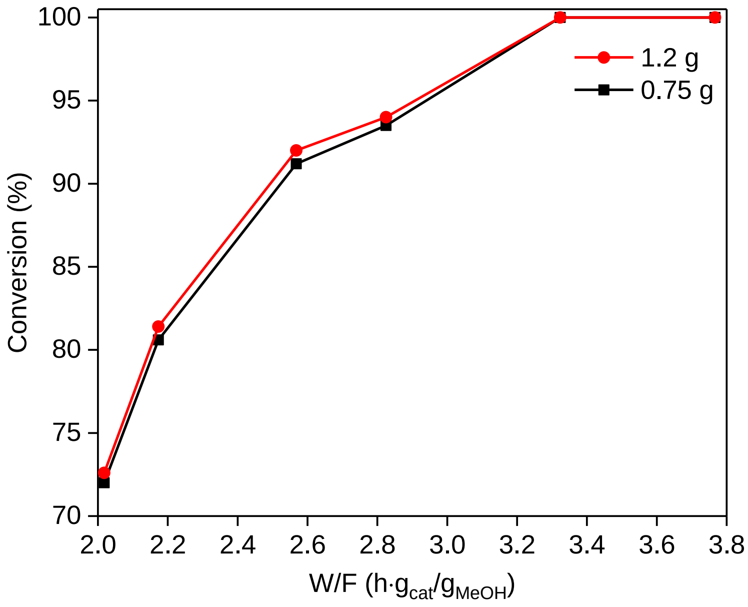

Supplementary Fig. 9. Studies of external mass transfer for MTO reactions on TaAlS-1(0.013/0.027/1). The effect of external mass transfer is negligible in the studied region. The conversion is calculated based on the unconverted methanol and dimethyl ether.

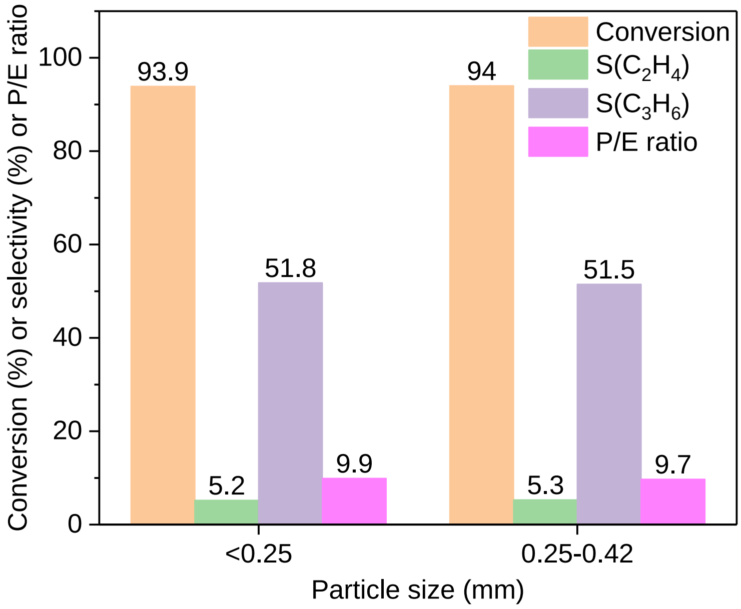

Supplementary Fig. 10. **S**tudies of internal mass transfer for MTO reactions on TaAlS-1(0.013/0.027/1). The effect of internal mass transfer is negligible in the studied region. The conversion is calculated based on the unconverted methanol and dimethyl ether.

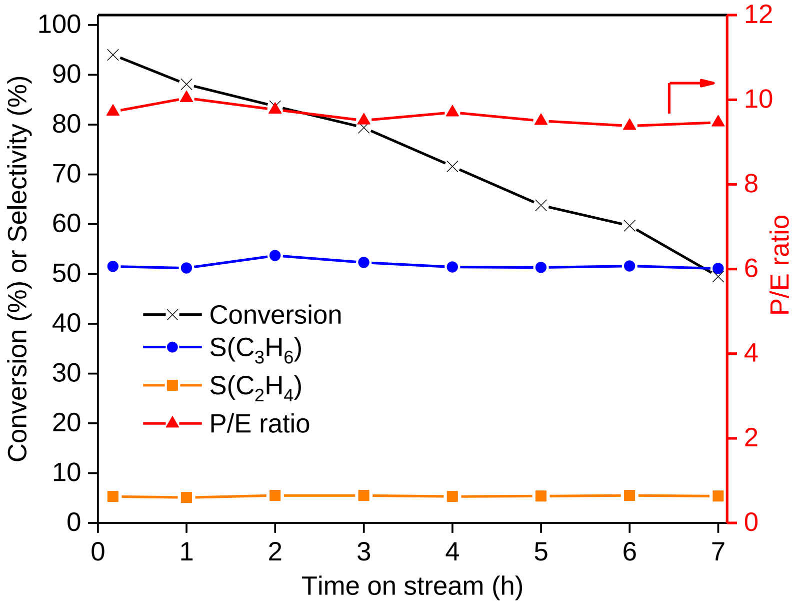

Supplementary Fig. 11. Lifetime study of TaAlS-1(0.013/0.027/1) at 400 °C, W/F of 2.83 h g_{cat} g_{MeOH}^{-1}, particle size of 250-420 μm, within the kinetically-controlled regime at the conversion of 94%. The conversion is calculated based on the unconverted methanol and dimethyl ether.

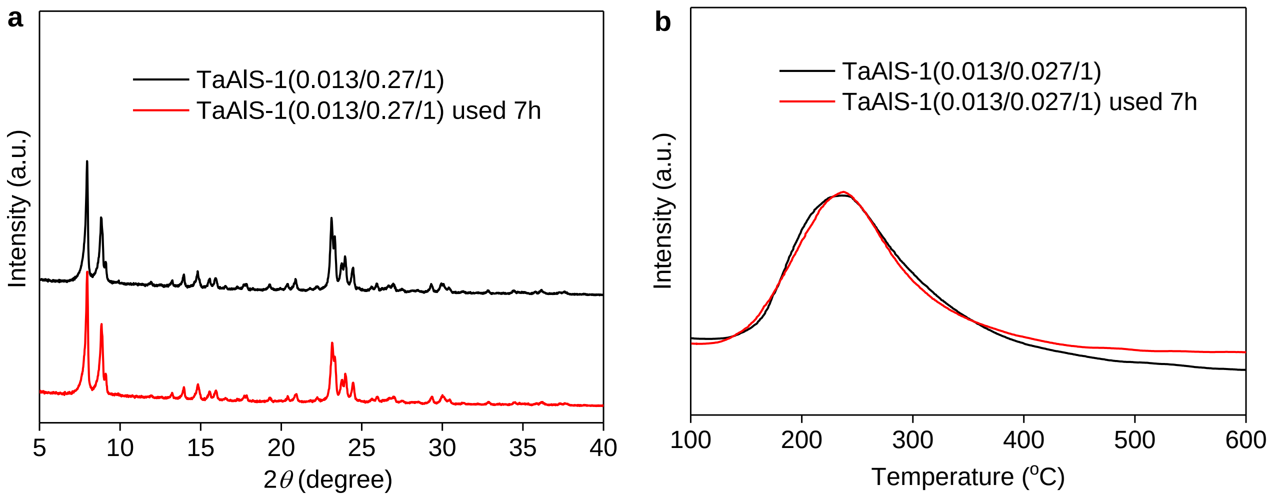

Supplementary Fig. 12. Comparisons of (**a**) XRD patterns (λ = 1.5406 Å) and (**b**) acidities determined by NH_3-TPD of TaAlS-1(0.013/0.027/1) before and after the conversion of methanol at 400 °C for 7h, W/F of 2.83 h g_{cat} g_{MeOH}^{-1}, particle size of 250-420 μm.

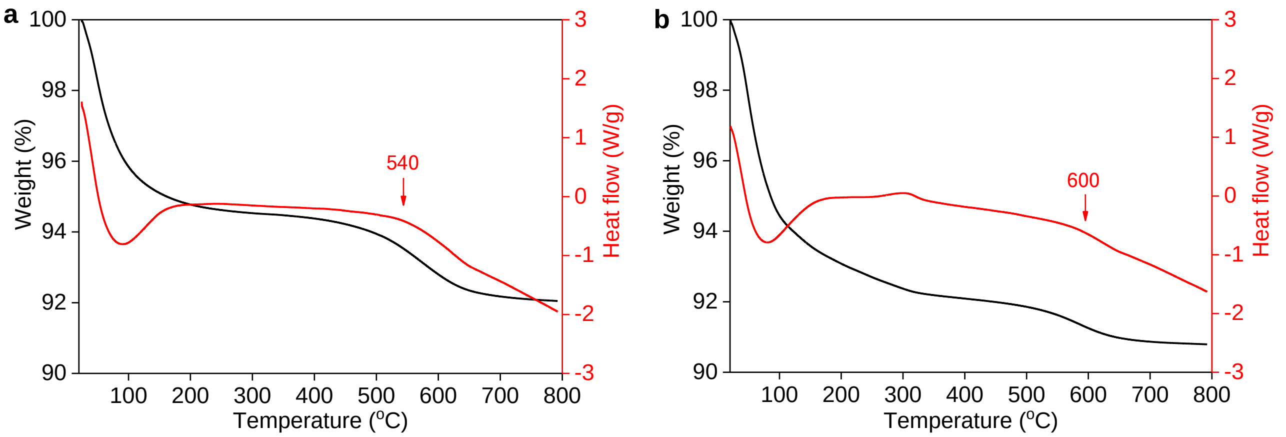

Supplementary Fig. 13. TGA (black) and DSC (red) plots of used TaAlS-1(0.013/0.027/1) (a) and HZSM-5(0.027/1) (b) catalysts after the conversion of methanol at 400 ºC for 54 h. The by-products (*e.g.*, coke) deposited on the surface of used HZSM-5(0.027/1) are more stable than those on used TaAlS-1(0.013/0.027/1).

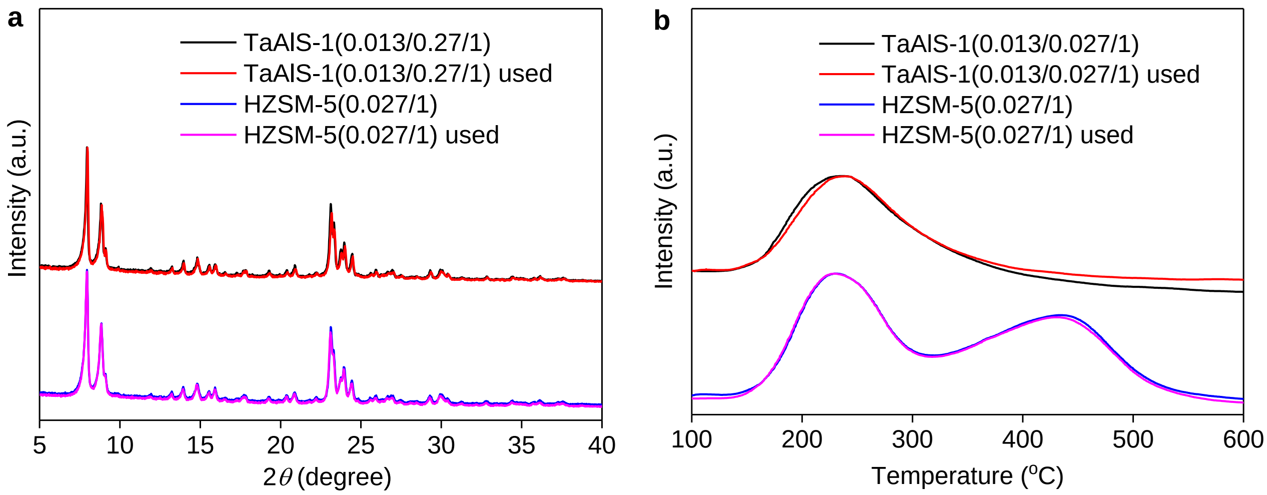

Supplementary Fig. 14. Comparison of (**a**) XRD patterns (λ = 1.5406 Å) and (**b**) acidities determined by NH_3-TPD of TaAlS-1(0.013/0.027/1) and HZSM-5(0.027/1) before and after the conversion of methanol at 400 °C for 54h.

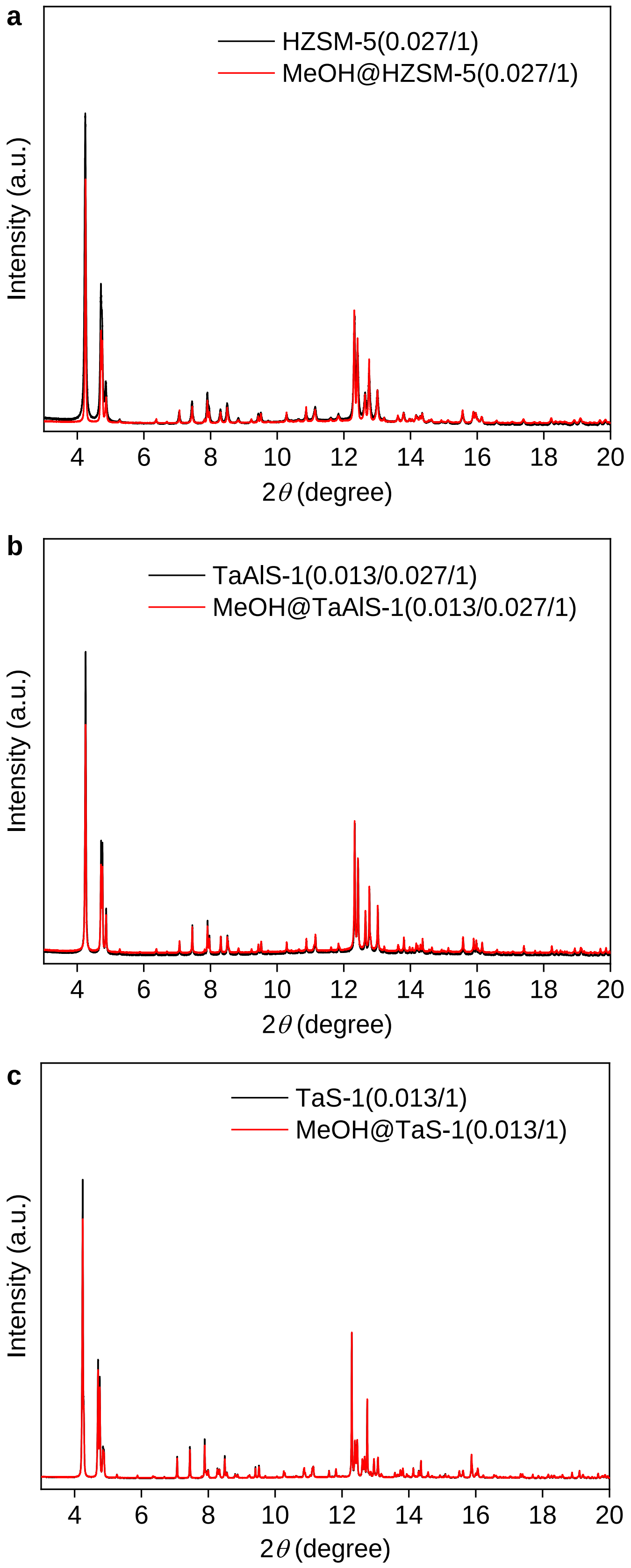

Supplementary Fig. 15. Comparison of SXPD patterns of (a) HZSM-5(0.027/1), (b) TaAlS-1(0.013/0.027/1) and (c) TaS-1(0.013/1) before and after adsorption of methanol at room temperature [λ = 0.82487(1) Å].

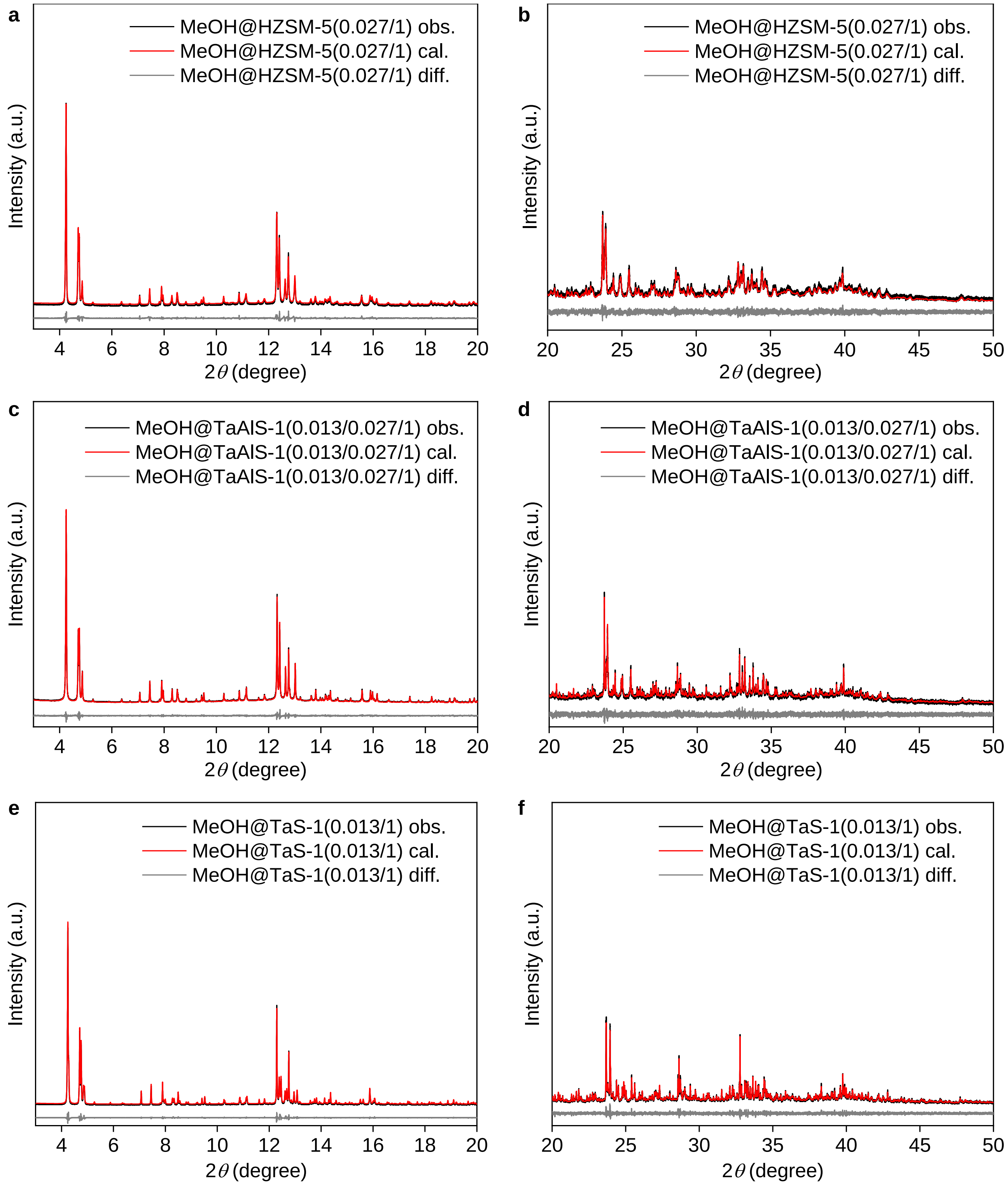

Supplementary Fig. 16. Comparison of the experimental data (black line) and Rietveld refinement (red line) and the difference between them (grey line) for SXPD patterns of (a,b) MeOH@HZSM-5(0.027/1), (c,d) MeOH@TaAlS-1(0.013/0.027/1), (e,f) MeOH@TaS-1(0.013/1) at room temperature at a 2θ range of (a,c,e) 3 - 20° and (b,d,f) 20 - 50° [λ = 0.82487(1) Å].

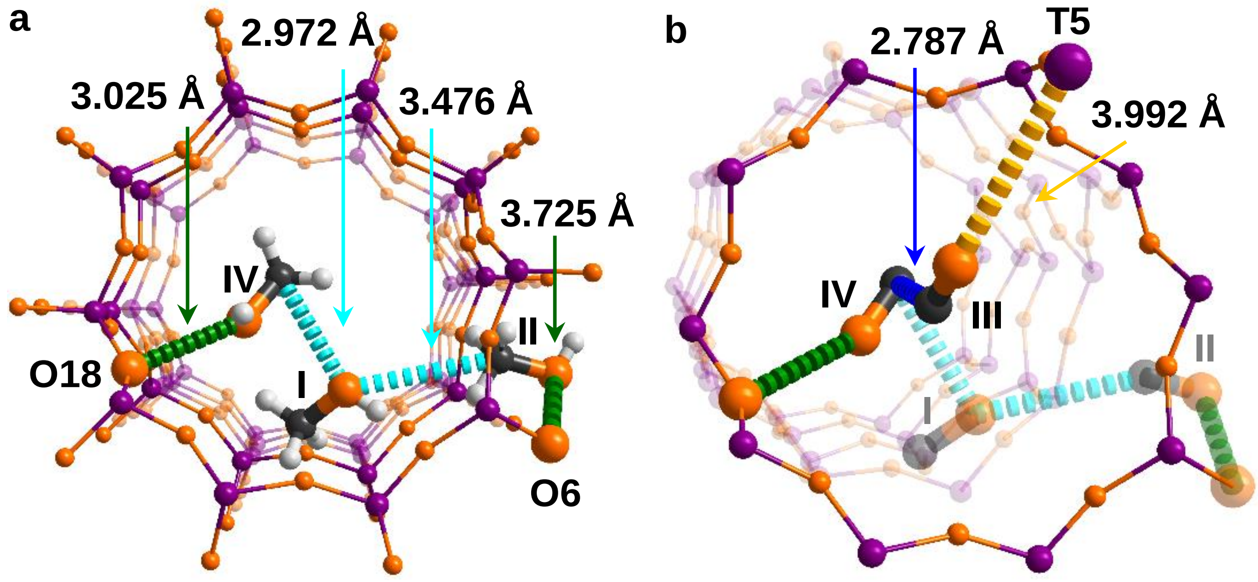

Supplementary Fig. 17. Enlarged and detailed views of MeOH^{I,II,IV} (**a**) and MeOH^{III} (**b**) in TaAlS-1(0.013/0.027/1). MeOH^{I,II,III,IV} are denoted as I, II, III and IV, respectively. MeOH molecules and the functional sites involved in the cooperative binding are highlighted using an amplified ball-and-stick model (Ta/Al/Si, violet; C, grey; O, orange; H, white). The O···O(H) interactions and O···Ta interactions are highlighted in green and yellow, respectively. The C···O distances and C···C distances are highlighted in cyan and blue, respectively. Owing to the uncertainty on locations of protons, all hydrogen bonds in this report are described as the distance between the O_{MeOH} and the $O_{zeolite}$ centres. The hydrogen atoms of MeOH are not displayed in **b**.

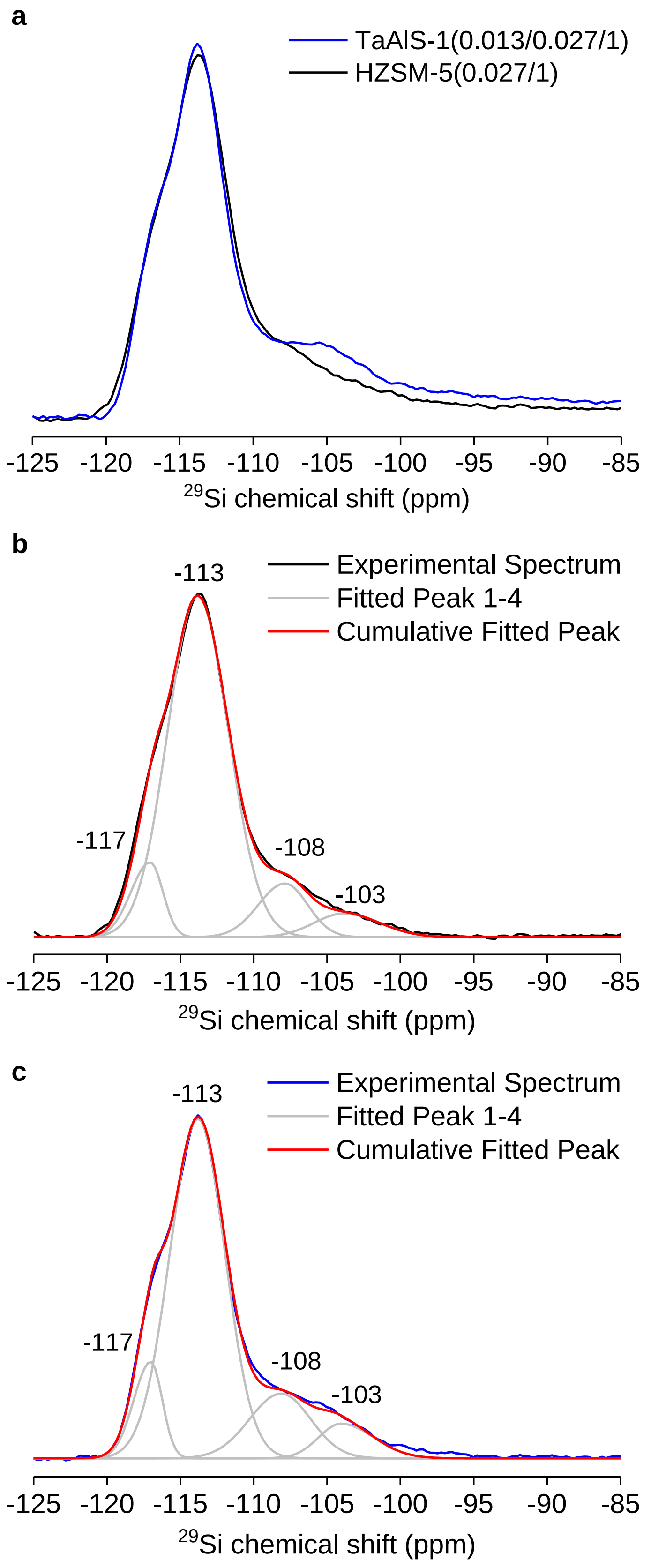

Supplementary Fig. 18. (a) ^{29}Si NMR spectra of HZSM-5 and TaAlS-1; de-convolution of ^{29}Si NMR spectra of HZSM-5 (b) and TaAlS-1 (c) by Guassian and Lorentzian line shapes.

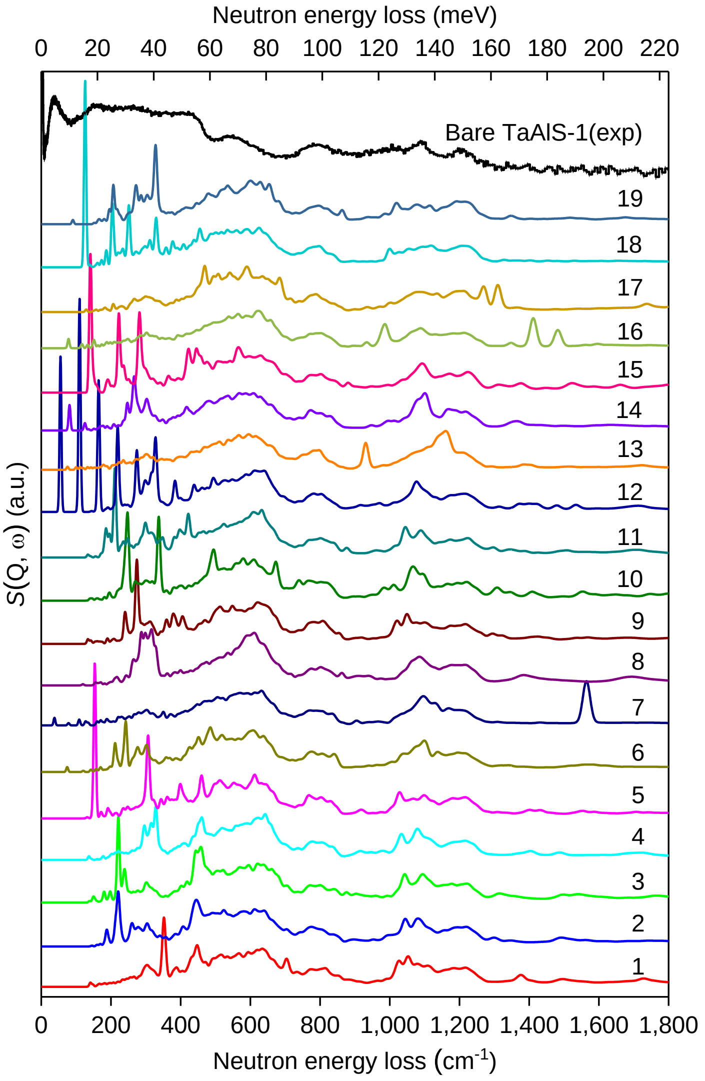

Supplementary Fig. 19. Comparison of the experimental INS spectrum of bare TaAlS-1(0.013/0.027/1) and calculated INS spectra for 19 types of Ta/Al/H sites.

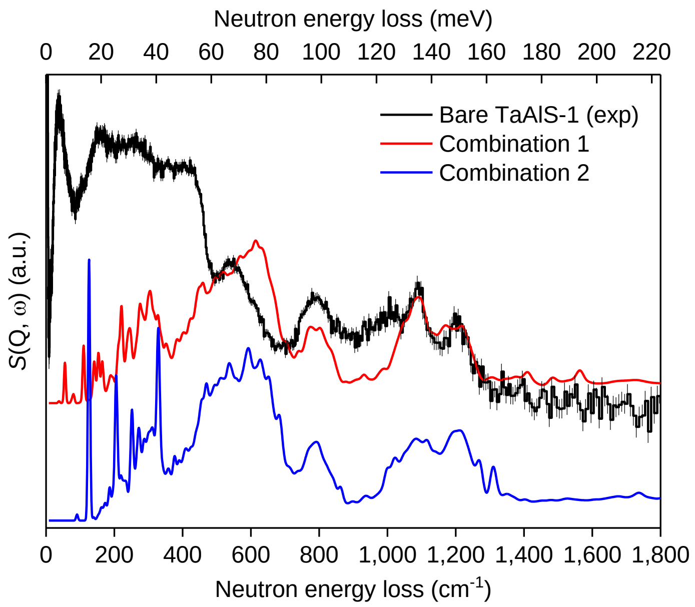

Supplementary Fig. 20. Comparison of the experimental INS spectrum of bare TaAlS-1(0.013/0.027/1) and combination of calculated INS spectra of different Ta/Al/H sites. Combination 1 = (Site 1 + Site 2 + …+ Site 16)/16; Combination 2 = (Site 17 + Site 18 + Site 19)/3. Abscissa is scaled by 0.96 for combination 1 and 2.

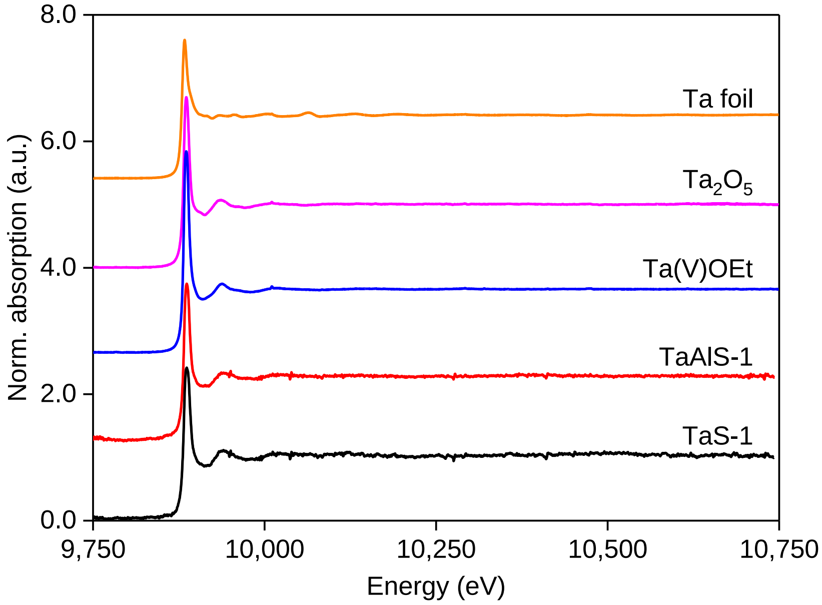

Supplementary Fig. 21. Ta L_3 edge X-ray absorption spectra of Ta foil, Ta(V) reference compounds and Ta-zeolites.

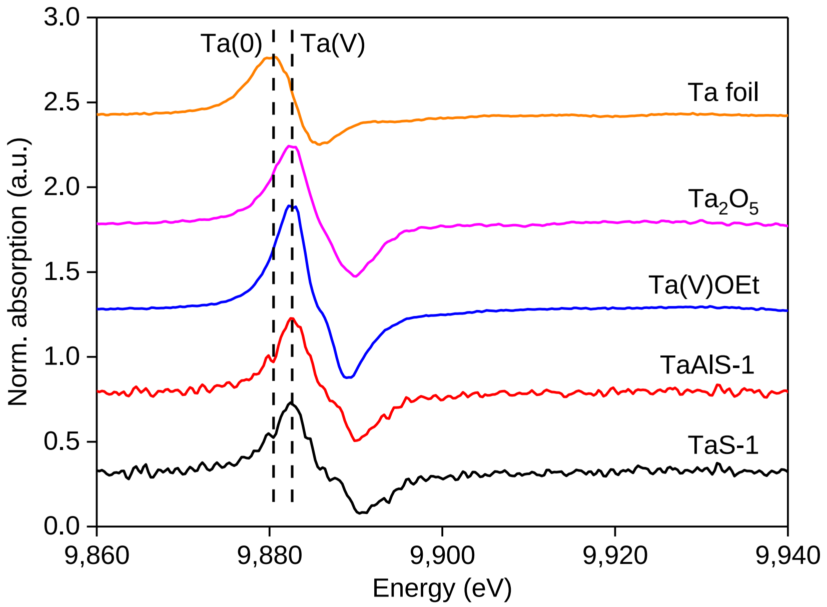

Supplementary Fig. 22. First derivate of Ta L_3 adsorption edge of Ta foil, Ta(V) reference compounds and Ta-zeolites.

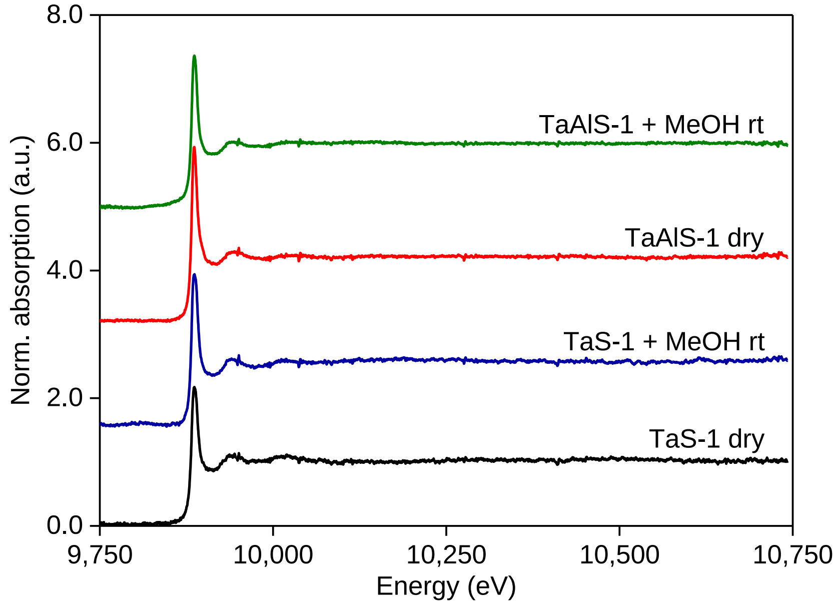

Supplementary Fig. 23. Ta L_3 edge X-ray absorption spectra of activated and MeOH-adsorbed Ta-zeolites.

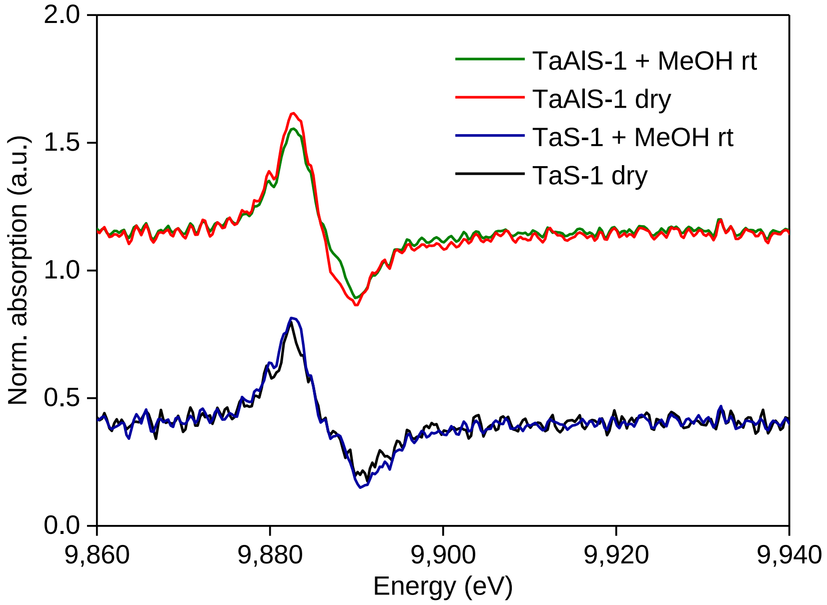

Supplementary Fig. 24. First derivative of Ta L_3 adsorption edge of activated and MeOH-adsorbed Ta-zeolites.

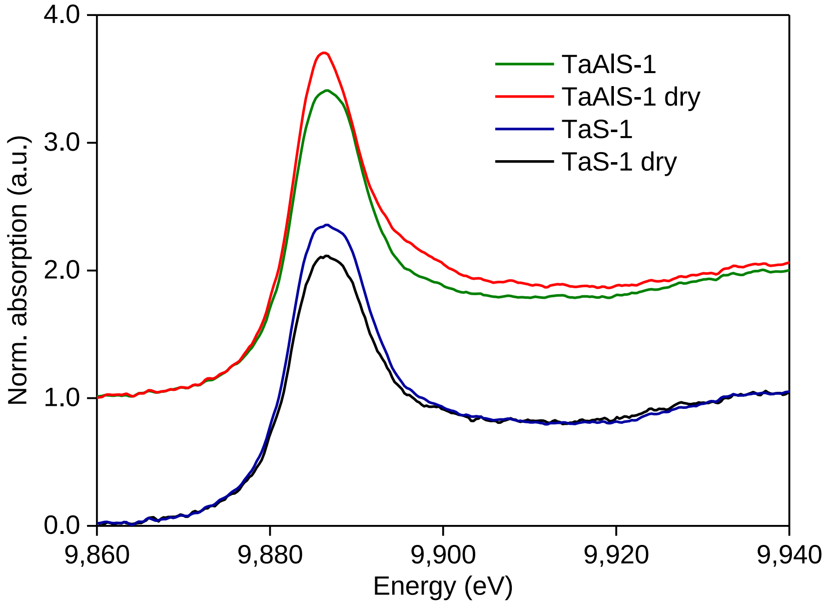

Supplementary Fig. 25. Ta L_3 edge X-ray absorption spectra of before and after activated Ta-zeolites. Due to the amphoteric nature of water, its adsorption, present within the as-synthesised materials prior to activation, gives rise to a similar influence on the Ta(V) site 5d-orbital occupancy as described in MeOH-loaded TaAlS-1.

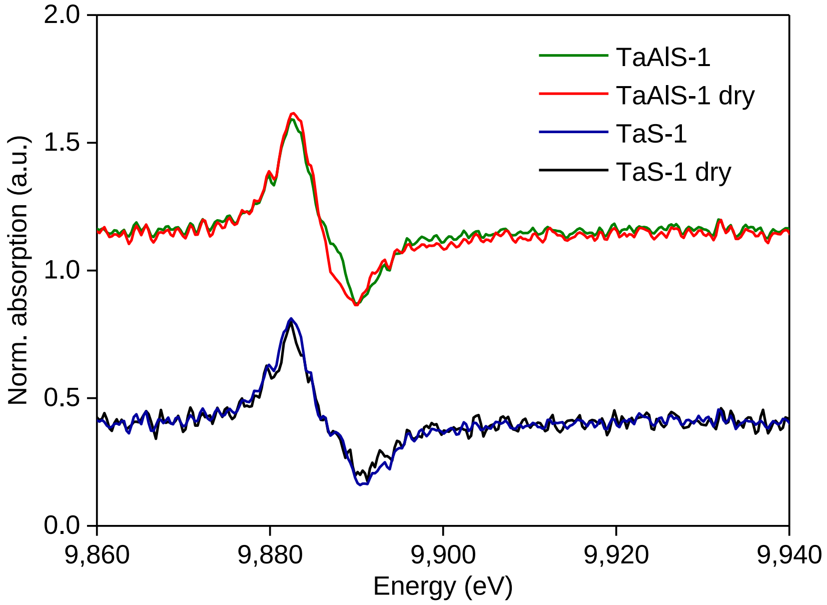

Supplementary Fig. 26. First derivative of Ta L_3 adsorption edge of before and after activated Ta-zeolites.

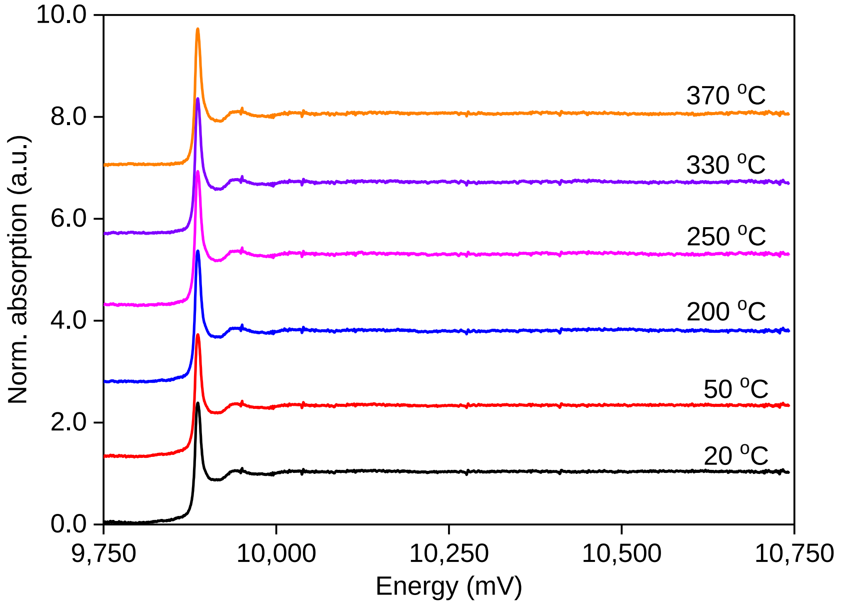

Supplementary Fig. 27. *Operando* Ta L_3 edge X-ray absorption spectra of TaAlS-1 zeolite during the conversion of methanol, only selected spectra are shown.

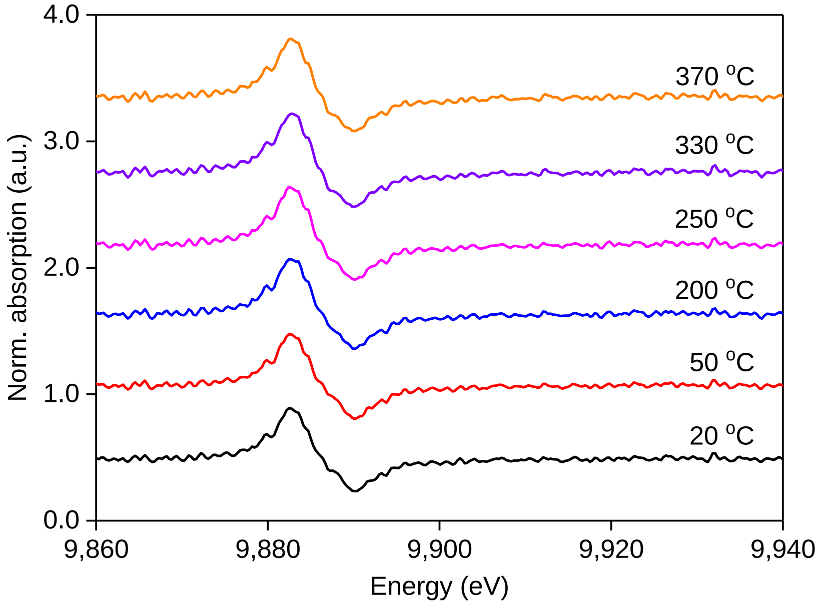

Supplementary Fig. 28. First derivative of *operando* Ta L_3 edge X-ray absorption spectra of TaAlS-1 zeolite during the conversion of methanol, only selected spectra shown.

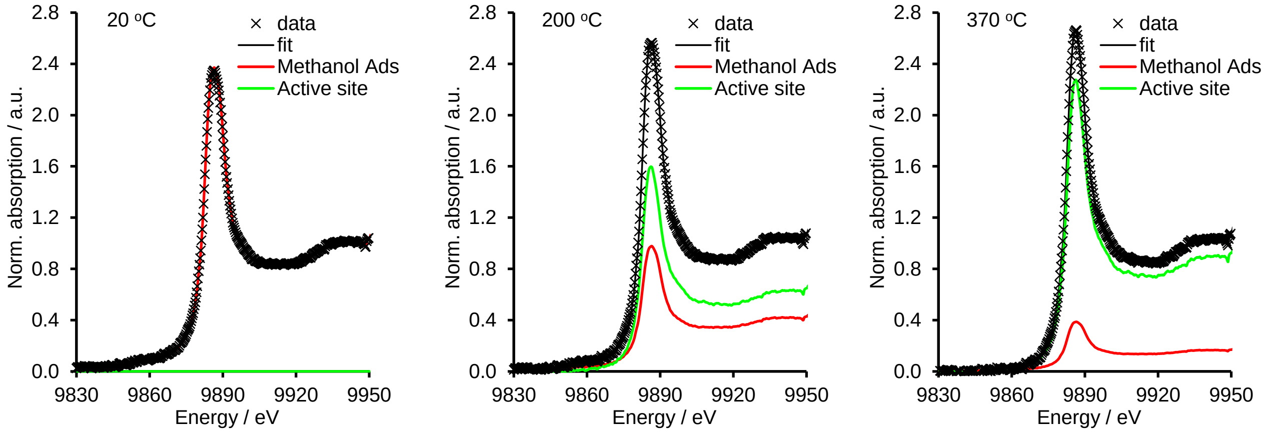

Supplementary Fig. 29. Deconvolution of XANES spectra via linear combination fitting to activated and adsorbed species representative fit result at 20 °C, 200 °C and 370 °C.

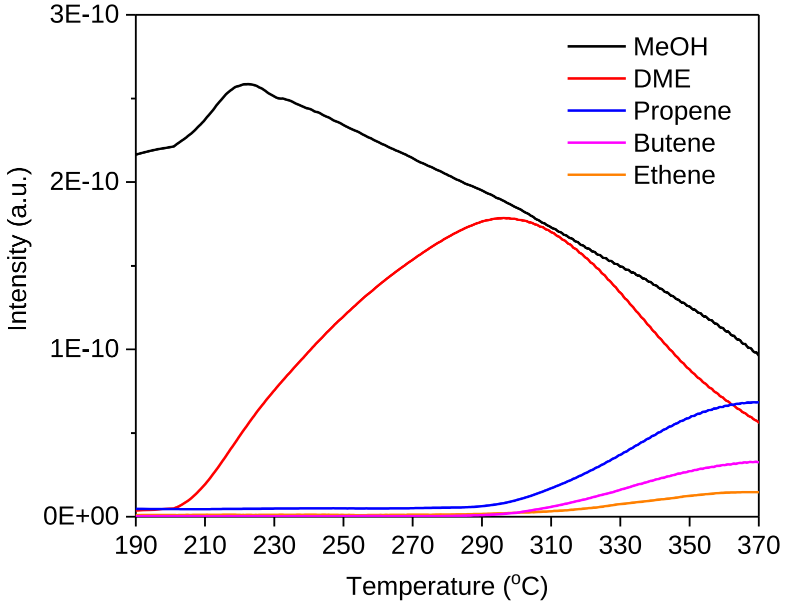

Supplementary Fig. 30. On-line mass spectra of reactants and products during the conversion of methanol on TaAlS-1 zeolite, for *operando* Ta L_3 edge X-ray absorption experiments.

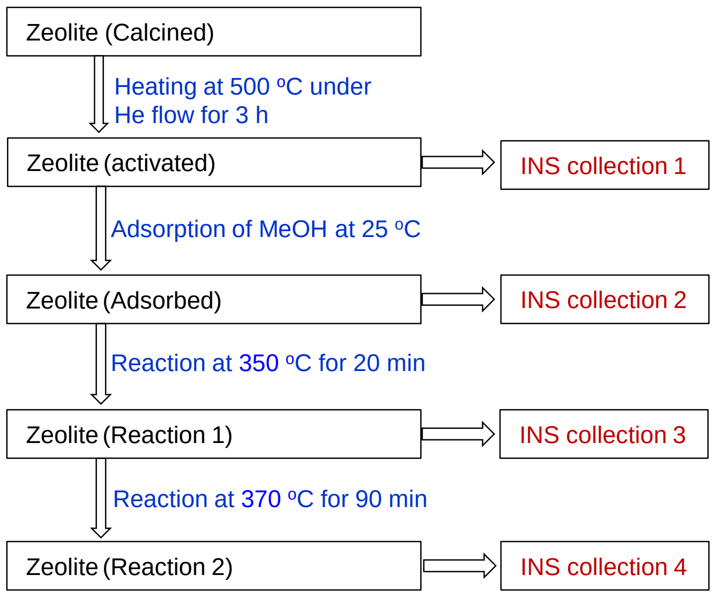

Supplementary Fig. 31. Schematic view of the procedure of INS experiment and data collection at TOSCA and MAPS. Before measurements at MAPS, the samples were transferred from the Inconel cell to an aluminium-can in order to reduce cell background signals.

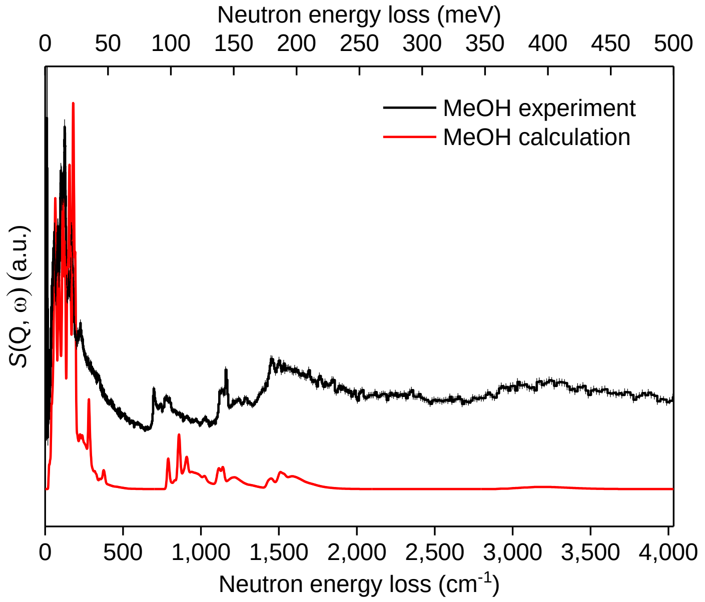

Supplementary Fig. 32. Comparison of the experimental INS spectra of condensed methanol in the solid-state (black, obtained in TOSCA) and calculated INS spectra of crystal methanol (red).

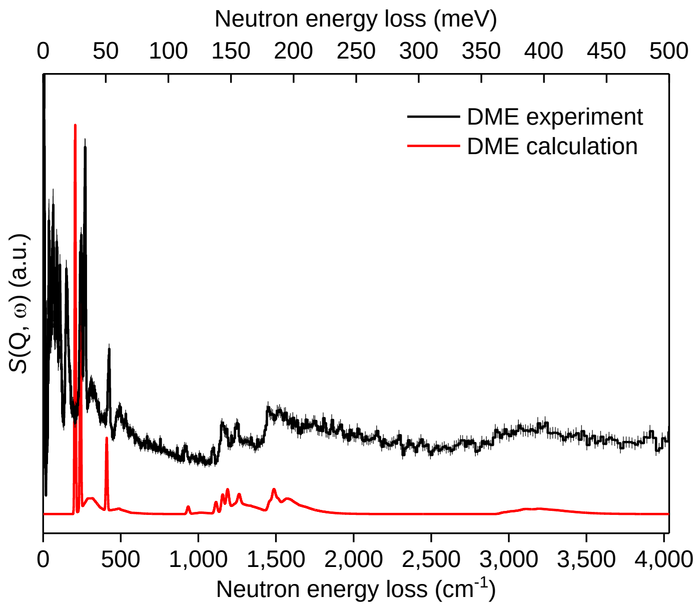

Supplementary Fig. 33. Comparison of the experimental INS spectra of condensed DME in the solid-state (black, obtained on TOSCA) and calculated INS spectra of single DME molecule (red).

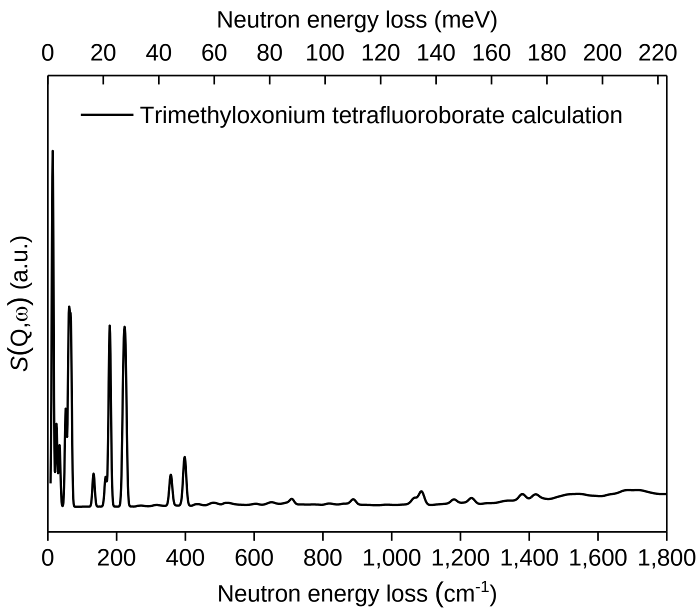

Supplementary Fig. 34. The calculated INS spectra of a single molecule of trimethyloxonium tetrafluoroborate.

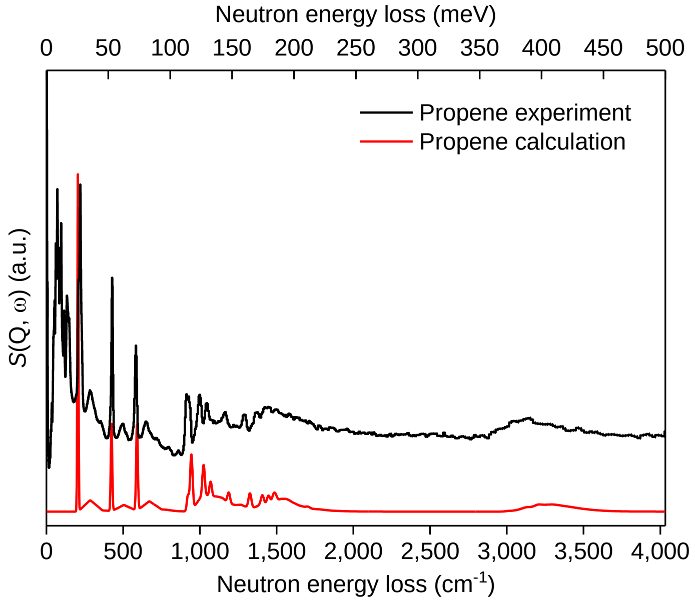

Supplementary Fig. 35. Comparison of the experimental INS spectra of condensed propene in the solid-state (black, obtained on TOSCA) and calculated INS spectra of single propene molecule (red). The lattice modes of condensed propene (below 150 cm^{-1}) are absent in the calculated propene spectrum as an isolated propene molecule has no lattice modes.

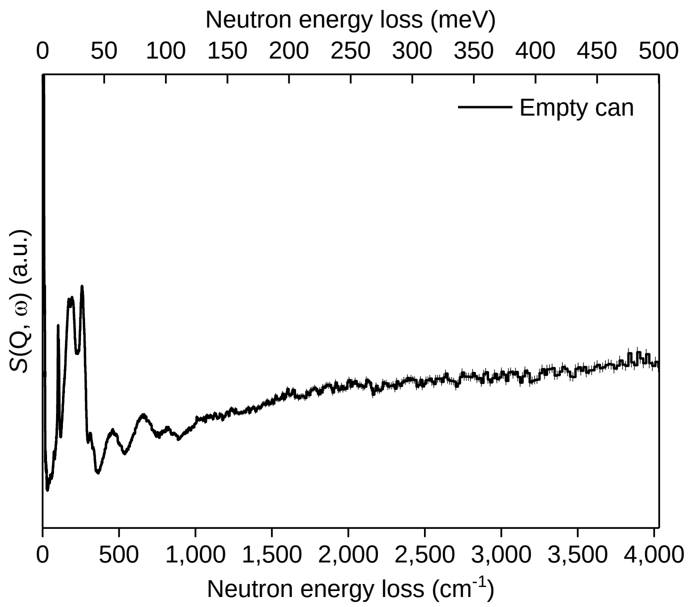

Supplementary Fig. 36. View of the INS spectrum of the empty Inconel cell used for TOSCA measurements. The empty can has features below 300 cm^{-1} and at approximately 457 and 659 cm^{-1}.

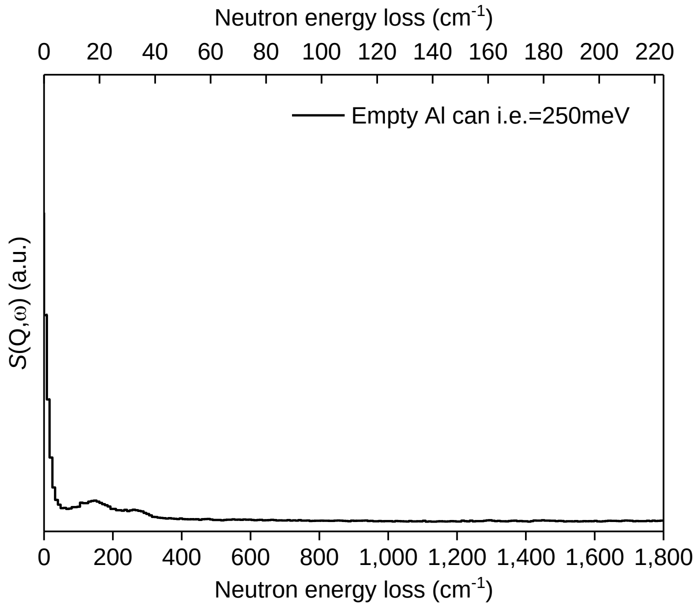

Supplementary Fig. 37. View of the INS spectrum for the empty aluminum can used for MAPS measurements with an incident energy of 250 meV. Spectra integrated over the momentum transfer range 0 $Å^{-1} \leq Q \leq 9$ $Å^{-1}$. The empty can has features at approximately 142 and 268 cm^{-1}.

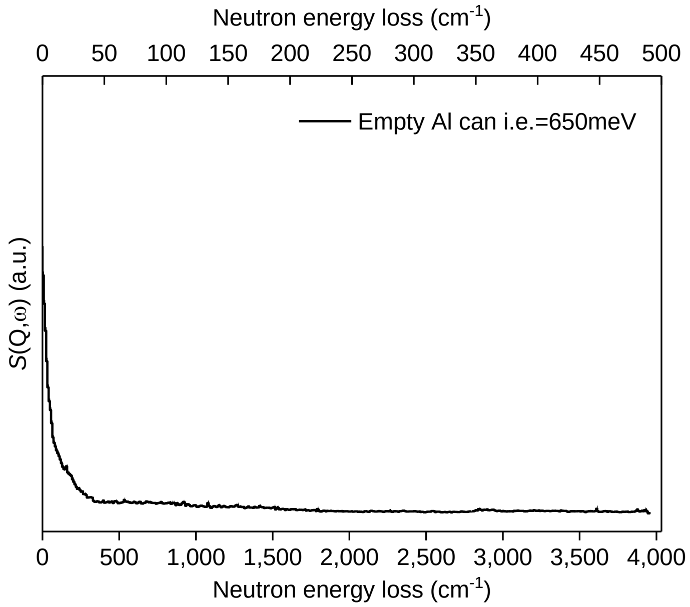

Supplementary Fig. 38. View of the INS spectrum for the empty aluminum can used for MAPS measurements with an incident energy of 650 meV. Spectra integrated over the momentum transfer range 0 $Å^{-1} \leq Q \leq 9$ $Å^{-1}$.

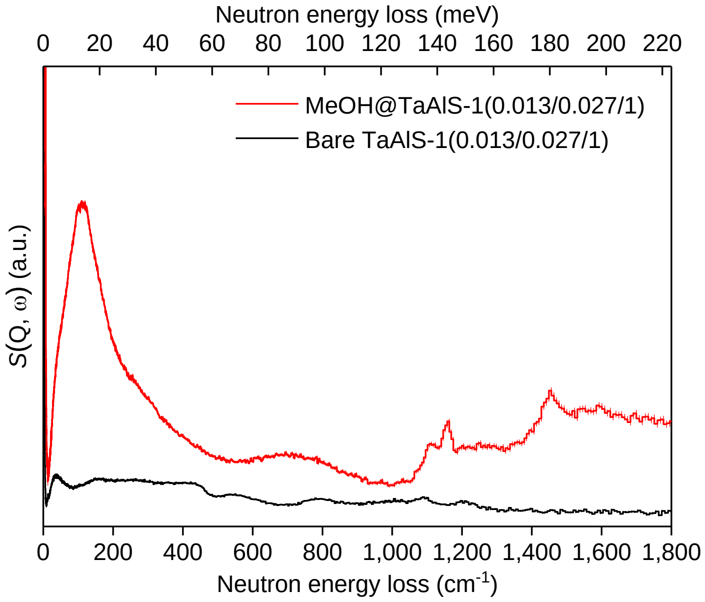

Supplementary Fig. 39. Comparison of the experimental INS spectra for bare and methanol-adsorbed catalyst. Spectra shown are after subtraction of the empty cell. The INS spectrum of the catalyst on methanol adsorption shows a significant increase in total intensity, demonstrating the binding of methanol to the catalyst sites.

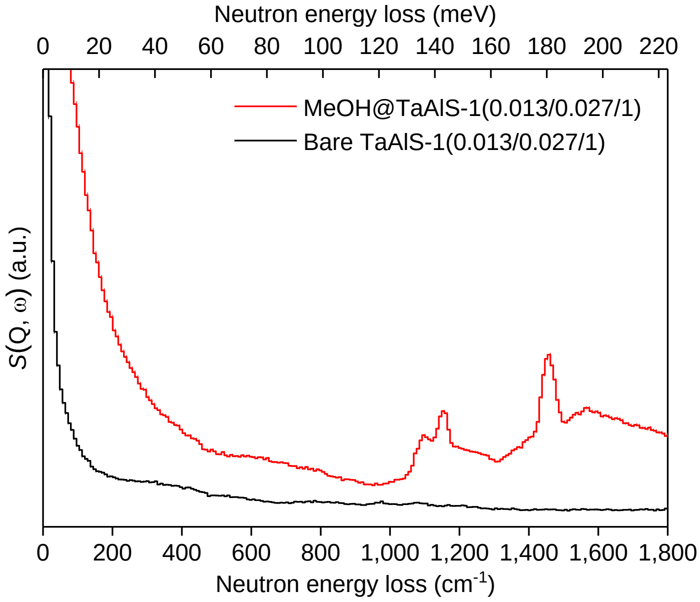

Supplementary Fig. 40. Comparison of the experimental INS spectra for bare and methanol-adsorbed catalyst with an incident energy of 250 meV at MAPS. Spectra were integrated over the momentum transfer range 0 $Å^{-1} \leq Q \leq 9$ $Å^{-1}$. Spectra shown are after subtraction of the empty cell. The INS spectrum of the catalyst on methanol adsorption shows a significant increase in total intensity, demonstrating the binding of methanol to the active sites.

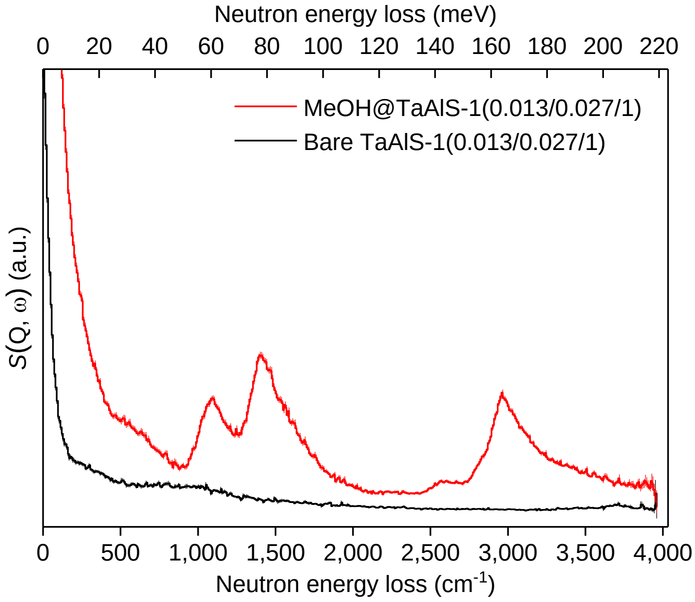

Supplementary Fig. 41. Comparison of the experimental INS spectra for bare and methanol-adsorbed catalyst with an incident energy of 650 meV. Spectra integrated over the momentum transfer range 0 $Å^{-1} \leq Q \leq 9 Å^{-1}$ (obtained in MAPS). Spectra shown are after subtraction of the empty cell. The INS spectrum of the catalyst on methanol adsorption at shows a significant increase in total intensity, demonstrating the binding of methanol to the active sites.

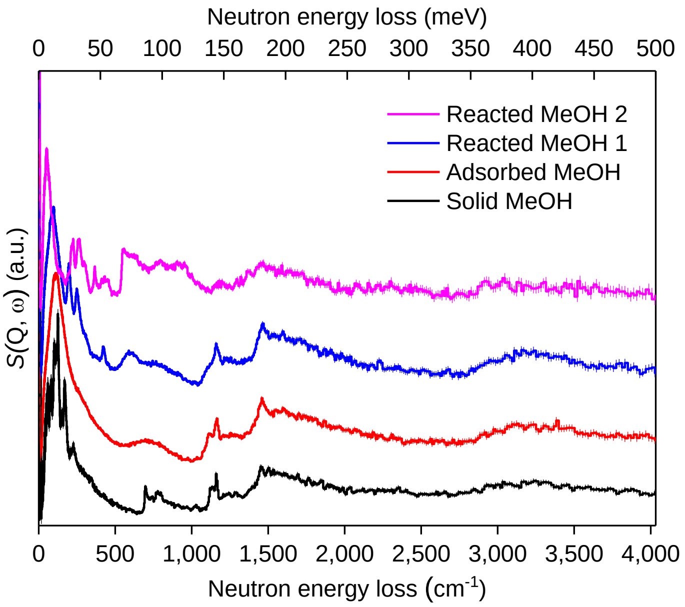

Supplementary Fig. 42. View of the range of 0-4000 cm^{-1} of INS spectrum for Fig. 5a.

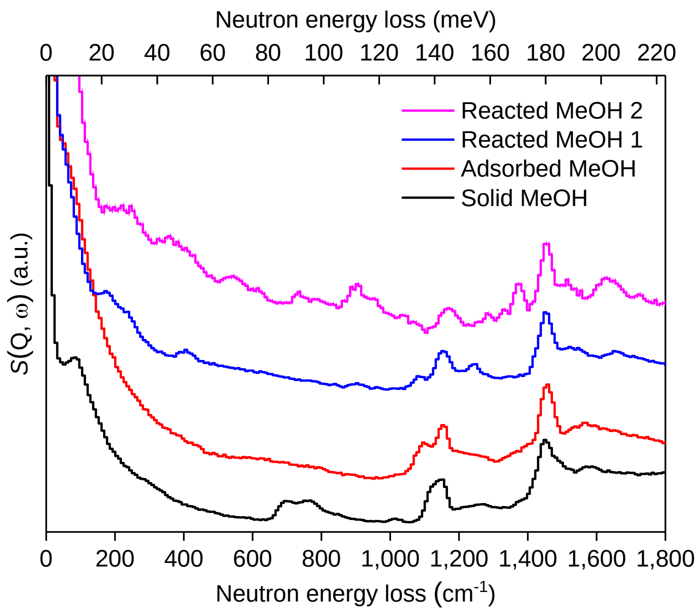

Supplementary Fig. 43. View of the range of 0-1800 cm^{-1} of INS spectrum for Fig. 5b.

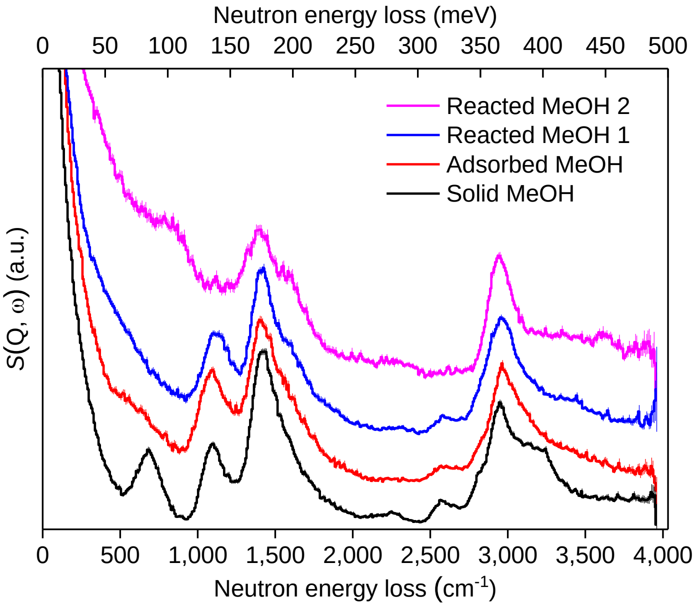

Supplementary Fig. 44. View of the range of 0-4000 cm^{-1} of INS spectrum for Fig. 5c.

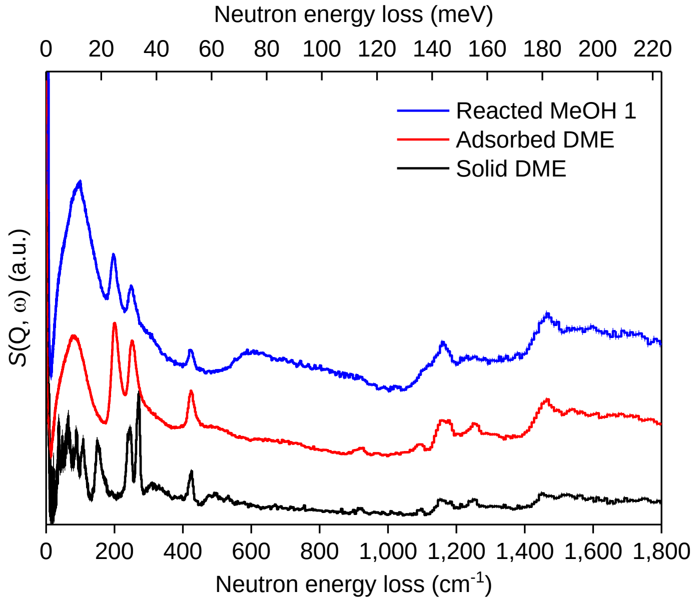

Supplementary Fig. 45. Comparison of the experimental INS spectra of solid DME, adsorbed DME and reacted methanol 1 (obtained on TOSCA).

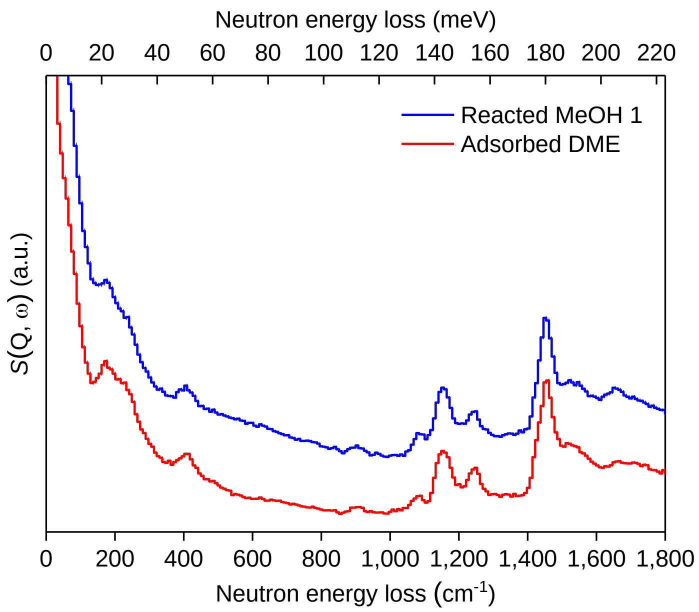

Supplementary Fig. 46. Comparison of the experimental INS spectra of adsorbed DME and reacted methanol 1 at an incident energy of 250 meV. Spectra integrated over the momentum transfer range 0 $Å^{-1} \leq Q \leq 9$ $Å^{-1}$ (obtained on MAPS).

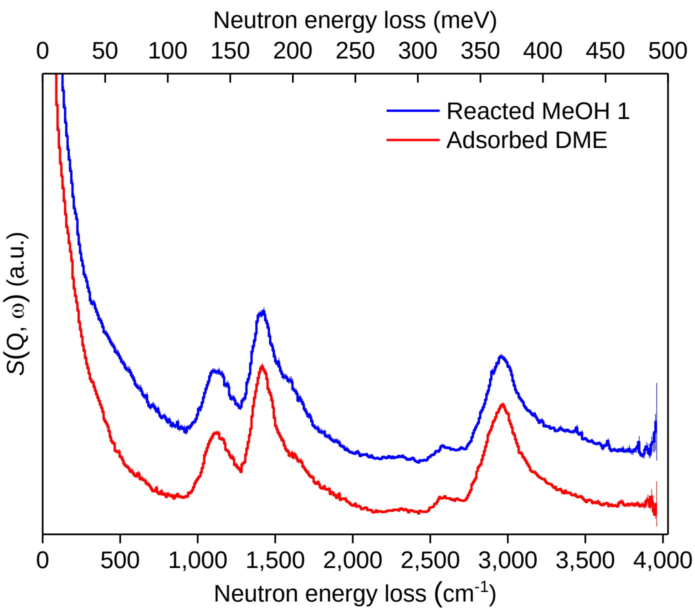

Supplementary Fig. 47. Comparison of the experimental INS spectra of adsorbed DME and reacted methanol 1 at an incident energy of 650 meV. Spectra integrated over the momentum transfer range 0 $Å^{-1} \leq Q \leq 9$ $Å^{-1}$ (obtained on MAPS).

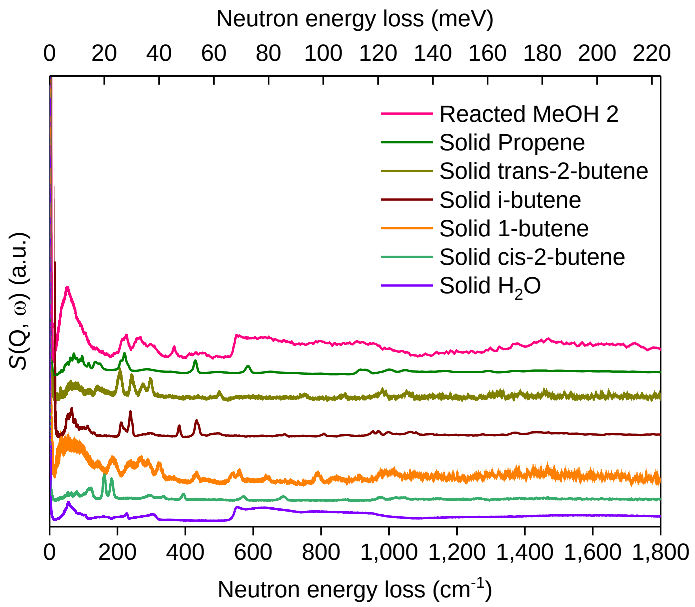

Supplementary Fig. 48. Comparison of the experimental INS spectra of propene, butene, H_2O and reacted methanol 2 (obtained on TOSCA).

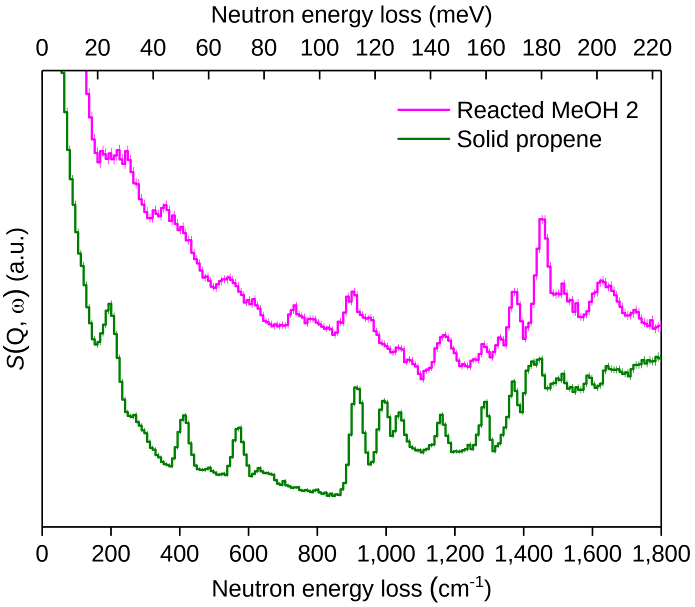

Supplementary Fig. 49. Comparison of the experimental INS spectra of solid propene and reacted methanol 2 with an incident energy of 250 meV on MAPS. Spectra integrated over the momentum transfer range 0 Å^{-1} ≤ Q ≤ 9 Å^{-1}. All the peaks of propene were found in the spectrum of reacted MeOH 2, indicating the formation of propene. The intensity of peaks at 916, 997, 1289, 1426 cm^{-1} (=CH_2 or =CH- vibrations) are weaker, due to interaction between propene and acid sites. The umbrella vibrational mode at 365 cm^{-1} is also visible. The peak at 736 cm^{-1} was assigned to the in-phase methylene rocking[31,32]. The presence of this peak is due to partial oligomerisation of propene during cooling, which matches previous observation[31,32]. The peaks at 546 and 1636 cm^{-1} are due to water from dehydration.

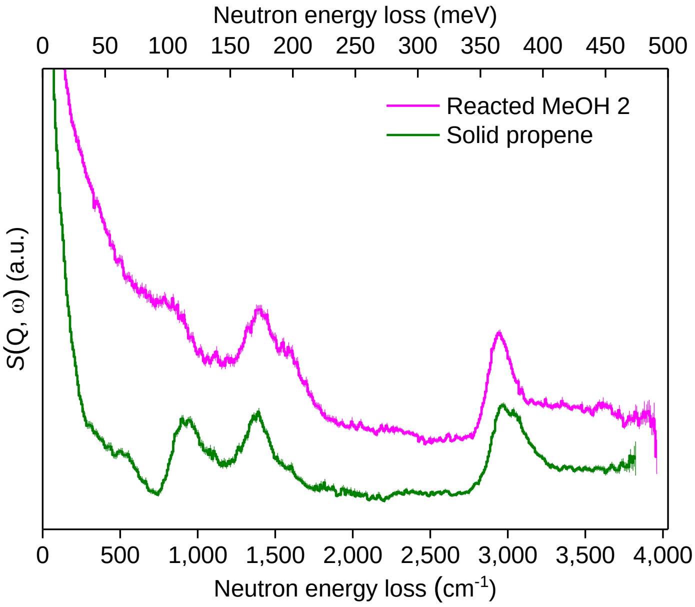

Supplementary Fig. 50. Comparison of the experimental INS spectra of solid propene and reacted methanol 2 with an incident energy of 650 meV. Spectra integrated over the momentum transfer range 0 $Å^{-1} \leq Q \leq 9$ $Å^{-1}$ (obtained on MAPS).

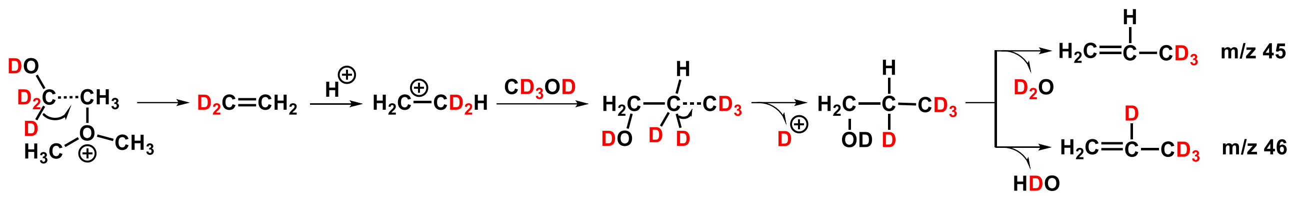

Supplementary Fig. 51. The proposed reaction pathways between TMO and CD_3OD to form $C_3H_3D_3$ and $C_3H_2D_4$.

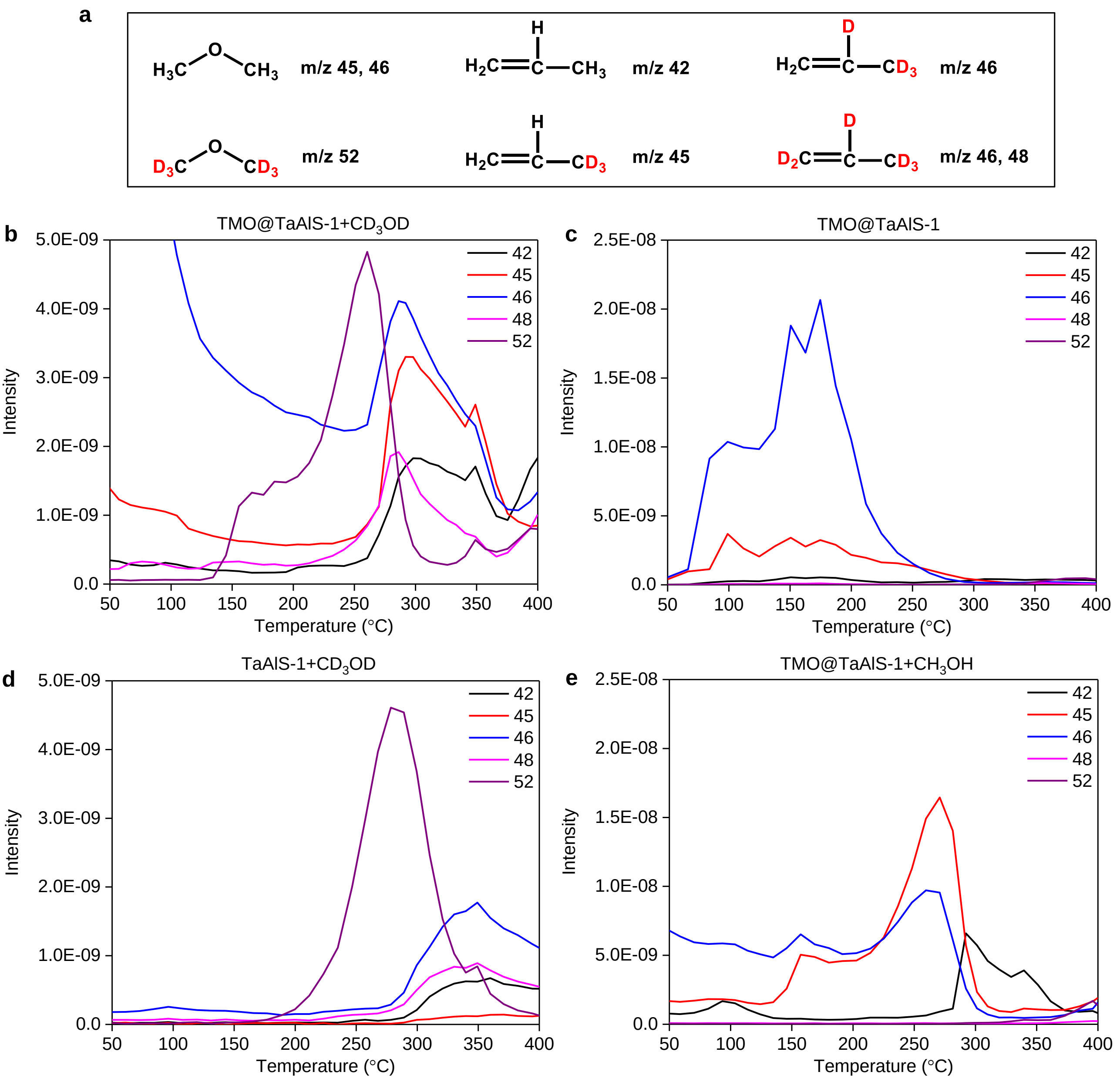

Supplementary Fig. 52. MS signals of DME and propene (**a**) and TP-MS plots of TMO@TaAlS-1+CD_3OD (**b**), TMO@TaAlS-1 (**c**), TaAlS-1+CD_3OD (**d**) and TMO@TaAlS-1+CH_3OH (**e**).

Supplementary Table

Supplementary Table 1. Comparison of the catalytic performance of reported catalysts for the conversion of pure methanol to olefins.

Catalyst	Reaction temperature (°C)	Conversion (%)	Selectivity (%)		P/E ratio	Lifetime (hour)	Reference
			C_2H_4	C_3H_6			
NbAlS-1(0.013/0.027/1)[a]	400	92	0.1	0.2	2	-	This work
TaAlS-1(0.013/0.027/1)[b]	400	100	6.2	53	8.5	54	This work
CaZSM-5_AE3	500	100	9.0	38	4.2	95	33
MgZSM-5_AE7	500	100	12	39	3.3	95	33
CaZSM-5_AE5	500	100	7.5	51	6.8	60	33
SrZSM-5_AE6	500	100	10	42	4.2	24	33
ZSM-5_Z1	500	100	18	30	1.7	13	33
Mesoporous ZSM-5_M1	500	100	5.0	38	7.6	33	33
Dealumination ZSM-5_M4	500	100	7.5	46	6.1	58	33
CON-type zeolite	500	100	22	60	2.7	25	34
High-Si beta zeolite	550	>99	5.9	58	9.8	23	35
RUB-13	350	100-95[c]	15	45	3.0	4.5	36
SAPO-14	450	99	16	66	4.1	0.3	37
SSZ-13	360	100	46	36	0.8	4.0	38
CHA-1	400	100-95[c]	56	30	0.5	11	39
ITE-type zeolite	400	100-95[c]	24	48	2.0	6.3	39
SSZ-39	350	100	9.6	21	2.2	16	40
ZSM-11	450	99.6	10	38	3.8	43	41
ZSM-22	400	99	4.5	30	6.7	1.3	42
SAPO-34	400	100	33	42	1.3	4.3	43
MCM-22	450	100	6.3	26	4.1	48	44

[a]The main product is dimethyl ether with selectivity of 99%; [b]Selectivity and P/E ratio values are average of all points between 0-54 h. [c]The lifetime tests started at full conversion and the lifetime is the period of time from the start of reaction to the conversion dropped to 95%.

Supplementary Table 2. Specific surface areas and pore volumes of all zeolites used in this study, data determined from N_2 sorption isotherms at 77 K and t-plot.

Samples	A_{BET} (m^2 g^{-1})[a]	A_{mic} (m^2 g^{-1})[b]	A_{ext} (m^2 g^{-1})[c]	V_{mic} (cm^3 g^{-1})[d]	V_{total} (cm^3 g^{-1})[e]	Crystallite size[f] (nm)
HZSM-5 (0.027/1)	433	335	99	0.17	0.29	200-500
TaS-1(0.008/1)	407	354	53	0.17	0.24	300-500
TaS-1(0.013/1)	402	341	61	0.17	0.25	300-500
TaAlS-1(0.008/0.027/1)	416	318	98	0.16	0.3	300-500
TaAlS-1(0.013/0.027/1)	424	320	104	0.15	0.32	300-500
TaAlS-1(0.013/0.013/1)	403	346	57	0.17	0.25	300-500
TaAlS-1(0.013/0.02/1)	409	343	66	0.17	0.26	300-500
HZSM-5 (0.027/1) used	434	339	95	0.17	0.28	200-500
TaAlS-1(0.013/0.027/1) used	423	318	105	0.15	0.33	300-500
TaAlS-1(0.013/0.027/1) used 7 h[g]	431	328	103	0.16	0.32	300-500

[a]BET specific surface area;
[b]Micropore surface area;
[c]External surface area;
[d]Micropore volume;
[e]Total pore volume;
[f]Obtainted from SEM;
[g]TaAlS-1(0.013/0.027/1) used at 400 °C, W/F of 2.83 h g_{cat} g_{MeOH}^{-1}, for 7 h.

Supplementary Table 3. Simulation parameters of CW EPR spectra at X-band Ta, Al and Si defect.

Signal	weight	Electron Spin	Nuclei Spin	Q (MHz), ŋ	g-tensor	g-Strain	A-tensor (MHz)	lw (mT)
TaS-1(Ta/Si=0.013/1)								
Ta defect	0.7	1/2	7/2	Q= 15.4; ŋ=0.32	[2.0152 1.9954 2.0032]	[0.0 0.0 0.0035]	[13.2 8.2 48]	[0 0.35]
Si defect	0.3	1/2	1/2	-	[2.0124 2.012 2.004]	-	-	[0 0.4]
TaAlS-1(Ta/Al/Si=0.013/0.027/1)								
Ta defect	0.75	1/2	7/2	Q= 15.4; ŋ=0.32	[2.0152 1.9954 2.0032]	[0.0 0.0 0.0035]	[13.2 8.2 48]	[0 0.85]
Si defect	0.25	1/2	1/2	-	[2.0124 2.012 2.004]	-	-	[0 0.85]
HZSM-5 (Al/Si=0.027/1)								
Al defect	0.7	1/2	5/2	-	[2.0012 2.009 2.04]	[0.0 0.003 0.04]	[19.0 19.0 21.0]	[0.0 0.3]
Si defect	0.3	1/2	1/2	-	[2.0124 2.012 2.004]	-	-	[0.0 0.4]

lw is the homogeneous Lorentzian linewidth, g and A are the corresponding g- and HFI-tensors. Q and ŋ are parameters of quadrupole tensor: Q[-1+ ŋ, -1- ŋ, 2], were Q= eeQqh/(4I(2I-1)), ŋ-anisotropy parameter

Supplementary Table 4. Simulation parameters of Pulse HYSCORE EPR spectra at X-band for Si and Al defects shown in Supplementary Fig. 5, lw is the homogeneous Lorentzian linewidth, *g* and *A* are the corresponding *g* and HFI-tensors.

HZSM-5 (Al/Si=0.027/1)				
Nuclei	Electron Spin	Nuclei Spin	*g*-tensor	*A*-tensor (MHz)
^{27}Al	1/2	5/2	[2.0012 2.009 2.04]	[19 19 21]
^{29}Si	1/2	1/2	[2.0141 2.00 2.0022]	[3.5 4.5 20]
TaAlS-1 (Ta/Al/Si=0.013/0.027/1)				
^{27}Al	1/2	5/2	[2.0012 2.009 2.04]	[18.5 18.5 21.0]
^{29}Si	1/2	1/2	[2.0141 2.00 2.0022]	[6.5 4.5 9.5]

Supplementary Table 5. Summary of acidity of all zeolites used in this work. Data were determined by NH_3-TPD studies.

Catalyst	Weak acid[a] (mmol g^{-1})	Strong acid[a] (mmol g^{-1})	Total acid[a] (mmol g^{-1})	L/B acid ratio[b]
HZSM-5(0.027/1)	0.15	0.25	0.4	0.39
HZSM-5(0.04/1)	0.24	0.28	0.52	0.37
HZSM-5(0.067/1)	0.33	0.3	0.63	0.27
TaS-1(0.008/1)	0.01	0	0.01	-
TaS-1(0.013/1)	0.02	0	0.02	-
TaAlS-1(0.008/0.027/1)	0.14	0	0.14	6.9
TaAlS-1(0.013/0.027/1)	0.18	0	0.18	7.8
TaAlS-1(0.013/0.02/1)	0.11	0	0.11	-
TaAlS-1(0.013/0.013/1)	0.05	0	0.05	-

[a]From NH_3-TPD.
[b]From Pyridine-IR.

Supplementary Table 6. Crystallographic data and details of TaAlS-1and zeolites samples after MeOH adsorption.

Samples	MeOH@HZSM-5(0.027/1)	TaAlS-1(0.013/0.027/1)	MeOH@TaAlS-1(.013/0.027/1)	TaS-1(0.013/1)	MeOH@TaS-1(0.013/1)
Crystal system	Orthorhombic	Orthorhombic	Orthorhombic	Monoclinic	Monoclinic
Space group	*Pnma*	*Pnma*	*Pnma*	$P2_1/n$	$P2_1/n$
Chemical formula	$Al_{2.5}Si_{93.5}O_{192}$ · 12.3MeOH	$Al_{2.5}Ta_{1.2}Si_{92.3}O_{192}$	$Al_{2.5}Ta_{1.2}Si_{92.3}O_{192}$ · 8.6MeOH	$Ta_{1.2}Si_{94.8}O_{192}$	$Ta_{1.2}Si_{94.8}O_{192}$ · 4.0MeOH
2θ range for refinement (°)	3 – 50	3 – 50	3 – 50	3 – 50	3 – 50
Detector	Multi-analyser crystals	Multi-analyser crystals	Multi-analyser crystals	Multi-analyser crystals	Multi-analyser crystals
Number of hkls	3220	3203	3209	6200	6200
Refinement methods	Rietveld	Rietveld	Rietveld	Rietveld	Rietveld
a (Å)	20.08635(9)	20.05316(5)	20.06500(5)	19.89501(4)	19.89402(4)
b (Å)	19.91624(9)	19.87888(6)	19.88840(5)	20.11998(4)	20.11925(3)
c (Å)	13.39546(7)	13.37708(4)	13.38156(4)	13.38518(3)	13.38204(3)
V (Å^3)	5358.78(4)	5332.57(3)	5340.05(3)	5357.66(2)	5355.92(2)
Beta angle (°)	90	90	90	90.56	90.58
$R_{wp}/R_{exp}/R_p$ (%)	6.273/3.501/4.820	5.352/3.546/4.165	5.147/3.568/3.977	6.079/3.338/4.630	5.770/3.226/4.403
Wavelength (Å)	0.82487(1)	0.82487(1)	0.82487(1)	0.82487(1)	0.82487(1)
Gof χ^2	1.792	1.509	1.442	1.821	1.789

Supplementary Table 7. Atomic parameters from the Rietveld refinement of MeOH@HZSM-5(0.027/1) at room temperature.

Species	Atom	*x*	*y*	*z*	SOF	B_{eq} (Å^2)	Wyckoff
HZSM-5(0.0271/)	Al1	-0.0768(3)	0.0530(3)	0.8288(4)	0.02629	1.259(19)	8d
	Al2	0.1869(3)	-0.0322(2)	0.3182(4)	0.02629	1.259(19)	8d
	Al3	0.2212(2)	-0.0574(3)	0.5291(4)	0.02629	1.259(19)	8d
	Al4	0.1187(3)	0.0599(3)	1.0300(4)	0.02629	1.259(19)	8d
	Al5	-0.0763(3)	-0.0264(3)	0.1868(4)	0.02629	1.259(19)	8d
	Al6	0.1869(3)	0.0609(3)	0.6734(4)	0.02629	1.259(19)	8d
	Al7	-0.0751(3)	0.6726(3)	0.8268(4)	0.02629	1.259(19)	8d
	Al8	0.1943(3)	0.1303(3)	0.3153(4)	0.02629	1.259(19)	8d
	Al9	0.2275(3)	0.1720(3)	0.5317(4)	0.02629	1.259(19)	8d
	Al10	0.1194(3)	0.6769(3)	1.0334(4)	0.02629	1.259(19)	8d
	Al11	-0.0678(3)	0.1292(3)	0.1834(4)	0.02629	1.259(19)	8d
	Al12	0.1855(3)	0.6732(2)	0.6847(4)	0.02629	1.259(19)	8d
	Si1	-0.0768(3)	0.0530(3)	0.8288(4)	0.97371	1.259(19)	8d
	Si2	0.1869(3)	-0.0322(2)	0.3182(4)	0.97371	1.259(19)	8d
	Si3	0.2212(2)	-0.0574(3)	0.5291(4)	0.97371	1.259(19)	8d
	Si4	0.1187(3)	0.0599(3)	1.0300(4)	0.97371	1.259(19)	8d
	Si5	-0.0763(3)	-0.0264(3)	0.1868(4)	0.97371	1.259(19)	8d
	Si6	0.1869(3)	0.0609(3)	0.6734(4)	0.97371	1.259(19)	8d
	Si7	-0.0751(3)	0.6726(3)	0.8268(4)	0.97371	1.259(19)	8d
	Si8	0.1943(3)	0.1303(3)	0.3153(4)	0.97371	1.259(19)	8d
	Si9	0.2275(3)	0.1720(3)	0.5317(4)	0.97371	1.259(19)	8d
	Si10	0.1194(3)	0.6769(3)	1.0334(4)	0.97371	1.259(19)	8d
	Si11	-0.0678(3)	0.1292(3)	0.1834(4)	0.97371	1.259(19)	8d
	Si12	0.1855(3)	0.6732(2)	0.6847(4)	0.97371	1.259(19)	8d
	O1	0.1241(6)	-0.0506(7)	0.2589(7)	1	1.36(4)	8d
	O2	0.1938(6)	-0.0583(6)	0.4130(6)	1	1.36(4)	8d
	O3	0.1991(5)	0.0592(5)	1.0178(5)	1	1.36(4)	8d
	O4	-0.0971(5)	-0.0718(5)	1.0803(8)	1	1.36(4)	8d
	O5	0.1205(5)	0.0544(6)	0.7273(7)	1	1.36(4)	8d
	O6	0.2455(5)	0.0547(7)	0.7539(7)	1	1.36(4)	8d
	O7	0.1266(5)	0.1634(5)	0.2745(7)	1	1.36(4)	8d
	O8	0.1938(6)	0.1552(5)	0.4262(6)	1	1.36(4)	8d
	O9	0.1872(5)	0.6498(4)	0.0297(6)	1	1.36(4)	8d
	O10	-0.0908(5)	0.1515(5)	1.0863(8)	1	1.36(4)	8d
	O11	0.1119(5)	0.6608(5)	0.7558(7)	1	1.36(4)	8d
	O12	0.2414(5)	0.6426(5)	0.7275(7)	1	1.36(4)	8d
	O13	0.1854(5)	0.0535(5)	0.3056(5)	1	1.36(4)	8d
	O14	-0.0847(4)	0.0521(6)	0.1721(6)	1	1.36(4)	8d
	O15	-0.0843(5)	0.1310(5)	0.8830(8)	1	1.36(4)	8d
	O16	-0.0874(5)	0.0113(5)	0.9276(7)	1	1.36(4)	8d
	O17	-0.0968(5)	0.6385(5)	-0.0571(7)	1	1.36(4)	8d
	O18	0.1788(5)	0.1272(5)	0.6218(7)	1	1.36(4)	8d

	O19	0.1987(6)	0.0016(5)	0.6024(7)	1	1.36(4)	8d
	O20	0.1943(6)	0.6283(5)	0.5830(7)	1	1.36(4)	8d
	O21	0.0007(6)	0.0449(6)	0.7946(7)	1	1.36(4)	8d
	O22	-0.0039(5)	0.6536(6)	0.7918(7)	1	1.36(4)	8d
	O23	-0.0794(8)	0.75	0.8701(10)	1	1.36(4)	4c
	O24	0.1986(8)	0.75	0.6558(9)	1	1.36(4)	4c
	O25	0.2183(7)	0.25	0.5655(10)	1	1.36(4)	4c
	O26	0.1052(8)	0.75	0.0834(10)	1	1.36(4)	4c
$MeOH^{I}$	C1	-0.0187(19)	0.761(6)	-0.755(3)	0.303(6)	15.0(8)	8d
	O1	0.034(3)	0.779(9)	-0.691(6)	0.303(6)	15.0(8)	8d
	H1	0.070(2)	0.776(10)	-0.721(9)	0.303(6)	18.0(10)	8d
	H2A	-0.019(4)	0.790(6)	-0.814(4)	0.303(6)	18.0(10)	8d
	H2B	-0.061(2)	0.765(6)	-0.720(5)	0.303(6)	18.0(10)	8d
	H2C	-0.012(4)	0.714(6)	-0.776(5)	0.303(6)	18.0(10)	8d
$MeOH^{II}$	C1	-0.2100(7)	-0.7883(18)	-0.3945(18)	0.500(5)	15.0(8)	8d
	O1	-0.2776(9)	-0.788(3)	-0.365(3)	0.500(5)	15.0(8)	8d
	H1	-0.3020(8)	-0.784(4)	-0.415(4)	0.500(5)	18.0(10)	8d
	H2A	-0.2021(18)	-0.8254(19)	-0.441(2)	0.500(5)	18.0(10)	8d
	H2B	-0.1816(13)	-0.7936(19)	-0.336(2)	0.500(5)	18.0(10)	8d
	H2C	-0.1996(17)	-0.7456(19)	-0.428(2)	0.500(5)	18.0(10)	8d
$MeOH^{III}$	C1	-0.493(2)	-0.103(2)	-0.052(4)	0.373(7)	15.0(8)	8d
	O1	-0.509(5)	-0.037(3)	-0.022(8)	0.373(7)	15.0(8)	8d
	H1	-0.529(8)	-0.038(6)	0.033(6)	0.373(7)	18.0(10)	8d
	H2A	-0.465(4)	-0.125(4)	-0.002(5)	0.373(7)	18.0(10)	8d
	H2B	-0.470(5)	-0.102(4)	-0.117(6)	0.373(7)	18.0(10)	8d
	H2C	-0.535(3)	-0.129(3)	-0.060(11)	0.373(7)	18.0(10)	8d
$MeOH^{IV}$	C1	-0.4612(19)	-0.773(2)	0.008(3)	0.356(7)	15.0(8)	8d
	O1	-0.467(4)	-0.828(4)	-0.059(6)	0.356(7)	15.0(8)	8d
	H1	-0.485(4)	-0.861(3)	-0.029(10)	0.356(7)	18.0(10)	8d
	H2A	-0.505(2)	-0.761(3)	0.032(4)	0.356(7)	18.0(10)	8d
	H2B	-0.441(2)	-0.735(3)	-0.027(5)	0.356(7)	18.0(10)	8d
	H2C	-0.433(3)	-0.786(4)	0.064(3)	0.356(7)	18.0(10)	8d

Supplementary Table 8. Atomic parameters from the Rietveld refinement of TaAlS-1(0.013/0.027/1) at room temperature.

Species	Atom	*x*	*y*	*z*	SOF	B_{eq} (Å^2)	Wyckoff
TaAlS-1(0.013/0.027/1)	Al1	-0.0770(3)	0.0553(4)	0.8341(4)	0.025962	2.21(2)	8d
	Al2	0.1897(3)	-0.0293(2)	0.3140(4)	0.025962	2.21(2)	8d
	Al3	0.2216(2)	-0.0629(3)	0.5300(4)	0.025962	2.21(2)	8d
	Al4	0.1216(2)	0.0621(3)	1.0281(4)	0.025962	2.21(2)	8d
	Al5	-0.0752(3)	-0.0298(3)	0.1854(4)	0.025962	2.21(2)	8d
	Al6	0.1873(3)	0.0595(3)	0.6739(4)	0.025962	2.21(2)	8d
	Al7	-0.0760(3)	0.6732(3)	0.8238(5)	0.025962	2.21(2)	8d
	Al8	0.1947(3)	0.1297(3)	0.3184(4)	0.025962	2.21(2)	8d
	Al9	0.2231(3)	0.1719(2)	0.5317(4)	0.025962	2.21(2)	8d
	Al10	0.1184(3)	0.6768(3)	1.0298(4)	0.025962	2.21(2)	8d
	Al11	-0.0678(3)	0.1310(3)	0.1815(4)	0.025962	2.21(2)	8d
	Al12	0.1878(3)	0.6727(2)	0.6836(4)	0.025962	2.21(2)	8d
	Ta1	-0.0770(3)	0.0553(4)	0.8341(4)	0.0125	2.21(2)	8d
	Ta2	0.1897(3)	-0.0293(2)	0.3140(4)	0.0125	2.21(2)	8d
	Ta3	0.2216(2)	-0.0629(3)	0.5300(4)	0.0125	2.21(2)	8d
	Ta4	0.1216(2)	0.0621(3)	1.0281(4)	0.0125	2.21(2)	8d
	Ta5	-0.0752(3)	-0.0298(3)	0.1854(4)	0.0125	2.21(2)	8d
	Ta6	0.1873(3)	0.0595(3)	0.6739(4)	0.0125	2.21(2)	8d
	Ta7	-0.0760(3)	0.6732(3)	0.8238(5)	0.0125	2.21(2)	8d
	Ta8	0.1947(3)	0.1297(3)	0.3184(4)	0.0125	2.21(2)	8d
	Ta9	0.2231(3)	0.1719(2)	0.5317(4)	0.0125	2.21(2)	8d
	Ta10	0.1184(3)	0.6768(3)	1.0298(4)	0.0125	2.21(2)	8d
	Ta11	-0.0678(3)	0.1310(3)	0.1815(4)	0.0125	2.21(2)	8d
	Ta12	0.1878(3)	0.6727(2)	0.6836(4)	0.0125	2.21(2)	8d
	Si1	-0.0770(3)	0.0553(4)	0.8341(4)	0.961539	2.21(2)	8d
	Si2	0.1897(3)	-0.0293(2)	0.3140(4)	0.961539	2.21(2)	8d
	Si3	0.2216(2)	-0.0629(3)	0.5300(4)	0.961539	2.21(2)	8d
	Si4	0.1216(2)	0.0621(3)	1.0281(4)	0.961539	2.21(2)	8d
	Si5	-0.0752(3)	-0.0298(3)	0.1854(4)	0.961539	2.21(2)	8d
	Si6	0.1873(3)	0.0595(3)	0.6739(4)	0.961539	2.21(2)	8d
	Si7	-0.0760(3)	0.6732(3)	0.8238(5)	0.961539	2.21(2)	8d
	Si8	0.1947(3)	0.1297(3)	0.3184(4)	0.961539	2.21(2)	8d
	Si9	0.2231(3)	0.1719(2)	0.5317(4)	0.961539	2.21(2)	8d
	Si10	0.1184(3)	0.6768(3)	1.0298(4)	0.961539	2.21(2)	8d
	Si11	-0.0678(3)	0.1310(3)	0.1815(4)	0.961539	2.21(2)	8d
	Si12	0.1878(3)	0.6727(2)	0.6836(4)	0.961539	2.21(2)	8d
	O1	0.1254(5)	-0.0578(7)	0.2476(6)	1	1.98(4)	8d
	O2	0.1928(6)	-0.0652(5)	0.4217(7)	1	1.98(4)	8d
	O3	0.2041(5)	0.0549(6)	1.0264(5)	1	1.98(4)	8d
	O4	-0.0963(4)	-0.0632(6)	1.0805(8)	1	1.98(4)	8d
	O5	0.1186(5)	0.0587(7)	0.7208(7)	1	1.98(4)	8d
	O6	0.2461(5)	0.0499(8)	0.7561(7)	1	1.98(4)	8d

	O7	0.1296(5)	0.1617(5)	0.2748(7)	1	1.98(4)	8d
	O8	0.1918(6)	0.1520(4)	0.4267(7)	1	1.98(4)	8d
	O9	0.1928(6)	0.6594(4)	0.0253(5)	1	1.98(4)	8d
	O10	-0.0939(5)	0.1594(5)	1.0797(8)	1	1.98(4)	8d
	O11	0.1126(5)	0.6557(5)	0.7401(7)	1	1.98(4)	8d
	O12	0.2425(6)	0.6524(6)	0.7521(8)	1	1.98(4)	8d
	O13	0.1972(5)	0.0438(5)	0.3135(5)	1	1.98(4)	8d
	O14	-0.0723(4)	0.0524(6)	0.1728(6)	1	1.98(4)	8d
	O15	-0.0836(5)	0.1254(6)	0.8858(8)	1	1.98(4)	8d
	O16	-0.0951(6)	-0.0054(5)	0.9196(8)	1	1.98(4)	8d
	O17	-0.0980(5)	0.6349(5)	-0.0575(7)	1	1.98(4)	8d
	O18	0.1853(7)	0.1280(5)	0.6170(7)	1	1.98(4)	8d
	O19	0.1961(8)	0.0021(5)	0.5949(7)	1	1.98(4)	8d
	O20	0.1959(7)	0.6309(5)	0.5832(7)	1	1.98(4)	8d
	O21	0.0013(5)	0.0489(8)	0.7896(7)	1	1.98(4)	8d
	O22	-0.0059(6)	0.6469(6)	0.7879(7)	1	1.98(4)	8d
	O23	-0.0893(7)	0.75	0.8609(10)	1	1.98(4)	4c
	O24	0.1885(10)	0.75	0.6460(9)	1	1.98(4)	4c
	O25	0.2146(7)	0.25	0.5653(10)	1	1.98(4)	4c
	O26	0.1057(7)	0.75	0.0869(10)	1	1.98(4)	4c

Supplementary Table 9. Atomic parameters from the Rietveld refinement of MeOH@TaAlS-1(0.013/0.027/1) at room temperature.

Species	Atom	*x*	*y*	*z*	SOF	B_{eq} (Å^2)	Wyckoff
TaAlS-1(0.013/0.027/1)	Al1	-0.0773(3)	0.0573(3)	0.8332(4)	0.025962	2.46(2)	8d
	Al2	0.1886(3)	-0.0296(2)	0.3164(4)	0.025962	2.46(2)	8d
	Al3	0.2213(2)	-0.0619(3)	0.5294(4)	0.025962	2.46(2)	8d
	Al4	0.1214(2)	0.0622(3)	1.0290(4)	0.025962	2.46(2)	8d
	Al5	-0.0767(3)	-0.0294(2)	0.1876(4)	0.025962	2.46(2)	8d
	Al6	0.1859(3)	0.0594(3)	0.6749(4)	0.025962	2.46(2)	8d
	Al7	-0.0760(3)	0.6731(2)	0.8230(4)	0.025962	2.46(2)	8d
	Al8	0.1929(3)	0.1308(3)	0.3184(3)	0.025962	2.46(2)	8d
	Al9	0.2254(2)	0.1703(2)	0.5347(4)	0.025962	2.46(2)	8d
	Al10	0.1175(3)	0.6772(3)	1.0310(4)	0.025962	2.46(2)	8d
	Al11	-0.0693(2)	0.1306(3)	0.1800(4)	0.025962	2.46(2)	8d
	Al12	0.1877(3)	0.6726(2)	0.6827(4)	0.025962	2.46(2)	8d
	Ta1	-0.0773(3)	0.0573(3)	0.8332(4)	0.0125	2.46(2)	8d
	Ta2	0.1886(3)	-0.0296(2)	0.3164(4)	0.0125	2.46(2)	8d
	Ta3	0.2213(2)	-0.0619(3)	0.5294(4)	0.0125	2.46(2)	8d
	Ta4	0.1214(2)	0.0622(3)	1.0290(4)	0.0125	2.46(2)	8d
	Ta5	-0.0767(3)	-0.0294(2)	0.1876(4)	0.0125	2.46(2)	8d
	Ta6	0.1859(3)	0.0594(3)	0.6749(4)	0.0125	2.46(2)	8d
	Ta7	-0.0760(3)	0.6731(2)	0.8230(4)	0.0125	2.46(2)	8d
	Ta8	0.1929(3)	0.1308(3)	0.3184(3)	0.0125	2.46(2)	8d
	Ta9	0.2254(2)	0.1703(2)	0.5347(4)	0.0125	2.46(2)	8d
	Ta10	0.1175(3)	0.6772(3)	1.0310(4)	0.0125	2.46(2)	8d
	Ta11	-0.0693(2)	0.1306(3)	0.1800(4)	0.0125	2.46(2)	8d
	Ta12	0.1877(3)	0.6726(2)	0.6827(4)	0.0125	2.46(2)	8d
	Si1	-0.0773(3)	0.0573(3)	0.8332(4)	0.961539	2.46(2)	8d
	Si2	0.1886(3)	-0.0296(2)	0.3164(4)	0.961539	2.46(2)	8d
	Si3	0.2213(2)	-0.0619(3)	0.5294(4)	0.961539	2.46(2)	8d
	Si4	0.1214(2)	0.0622(3)	1.0290(4)	0.961539	2.46(2)	8d
	Si5	-0.0767(3)	-0.0294(2)	0.1876(4)	0.961539	2.46(2)	8d
	Si6	0.1859(3)	0.0594(3)	0.6749(4)	0.961539	2.46(2)	8d
	Si7	-0.0760(3)	0.6731(2)	0.8230(4)	0.961539	2.46(2)	8d
	Si8	0.1929(3)	0.1308(3)	0.3184(3)	0.961539	2.46(2)	8d
	Si9	0.2254(2)	0.1703(2)	0.5347(4)	0.961539	2.46(2)	8d
	Si10	0.1175(3)	0.6772(3)	1.0310(4)	0.961539	2.46(2)	8d
	Si11	-0.0693(2)	0.1306(3)	0.1800(4)	0.961539	2.46(2)	8d
	Si12	0.1877(3)	0.6726(2)	0.6827(4)	0.961539	2.46(2)	8d
	O1	0.1261(5)	-0.0517(7)	0.2515(6)	1	2.35(4)	8d
	O2	0.1935(6)	-0.0640(5)	0.4233(7)	1	2.35(4)	8d
	O3	0.2017(5)	0.0524(6)	1.0261(5)	1	2.35(4)	8d
	O4	-0.0943(4)	-0.0641(6)	1.0789(7)	1	2.35(4)	8d
	O5	0.1194(5)	0.0571(6)	0.7253(6)	1	2.35(4)	8d
	O6	0.2442(4)	0.0602(6)	0.7606(6)	1	2.35(4)	8d

	O7	0.1292(5)	0.1616(5)	0.2707(7)	1	2.35(4)	8d
	O8	0.1915(6)	0.1521(4)	0.4302(6)	1	2.35(4)	8d
	O9	0.1933(6)	0.6576(4)	0.0264(5)	1	2.35(4)	8d
	O10	-0.0926(5)	0.1604(5)	1.0803(8)	1	2.35(4)	8d
	O11	0.1117(6)	0.6545(5)	0.7361(7)	1	2.35(4)	8d
	O12	0.2451(5)	0.6505(5)	0.7498(8)	1	2.35(4)	8d
	O13	0.1854(4)	0.0453(5)	0.3071(5)	1	2.35(4)	8d
	O14	-0.0805(4)	0.0558(6)	0.1722(6)	1	2.35(4)	8d
	O15	-0.0816(5)	0.1276(5)	0.8931(8)	1	2.35(4)	8d
	O16	-0.0914(6)	0.0006(6)	0.9155(8)	1	2.35(4)	8d
	O17	-0.0939(5)	0.6347(5)	-0.0624(7)	1	2.35(4)	8d
	O18	0.1838(5)	0.1290(5)	0.6262(6)	1	2.35(4)	8d
	O19	0.1976(7)	0.0014(5)	0.5938(7)	1	2.35(4)	8d
	O20	0.1950(7)	0.6287(5)	0.5872(6)	1	2.35(4)	8d
	O21	-0.0020(5)	0.0480(6)	0.7951(7)	1	2.35(4)	8d
	O22	-0.0028(6)	0.6513(6)	0.7871(7)	1	2.35(4)	8d
	O23	-0.0881(7)	0.75	0.8604(9)	1	2.35(4)	4c
	O24	0.1910(9)	0.75	0.6474(9)	1	2.35(4)	4c
	O25	0.2170(6)	0.25	0.5623(9)	1	2.35(4)	4c
	O26	0.1086(8)	0.75	0.0829(10)	1	2.35(4)	4c
$MeOH^{I}$	C1	-0.002(3)	0.765(5)	-0.724(3)	0.242(6)	15.0(11)	8d
	O1	0.047(4)	0.796(7)	-0.664(7)	0.242(6)	15.0(11)	8d
	H1	0.081(4)	0.805(8)	-0.698(10)	0.242(6)	18.0(13)	8d
	H2A	-0.014(4)	0.796(5)	-0.778(4)	0.242(6)	18.0(13)	8d
	H2B	-0.041(3)	0.754(5)	-0.684(6)	0.242(6)	18.0(13)	4c
	H2C	0.016(4)	0.723(5)	-0.752(5)	0.242(6)	18.0(13)	8d
$MeOH^{II}$	C1	-0.2166(11)	-0.789(2)	-0.388(3)	0.317(5)	15.0(11)	8d
	O1	-0.2812(19)	-0.767(5)	-0.361(5)	0.317(5)	15.0(11)	8d
	H1	-0.303(2)	-0.757(5)	-0.413(6)	0.317(5)	18.0(13)	8d
	H2A	-0.220(3)	-0.829(2)	-0.431(3)	0.317(5)	18.0(13)	8d
	H2B	-0.1915(18)	-0.801(3)	-0.328(3)	0.317(5)	18.0(13)	8d
	H2C	-0.193(2)	-0.753(3)	-0.424(3)	0.317(5)	18.0(13)	4c
$MeOH^{III}$	C1	-0.513(3)	-0.100(2)	-0.004(4)	0.300(5)	15.0(11)	8d
	O1	-0.504(5)	-0.040(3)	-0.060(7)	0.300(5)	15.0(11)	8d
	H1	-0.512(6)	-0.049(5)	-0.121(6)	0.300(5)	18.0(13)	8d
	H2A	-0.559(3)	-0.116(3)	-0.011(6)	0.300(5)	18.0(13)	8d
	H2B	-0.504(3)	-0.091(4)	0.067(5)	0.300(5)	18.0(13)	8d
	H2C	-0.482(3)	-0.134(3)	-0.028(5)	0.300(5)	18.0(13)	8d
$MeOH^{IV}$	C1	-0.475(3)	-0.761(5)	0.023(4)	0.214(6)	15.0(11)	8d
	O1	-0.435(5)	-0.793(8)	-0.049(8)	0.214(6)	15.0(11)	8d
	H1	-0.435(6)	-0.835(7)	-0.038(14)	0.214(6)	18.0(13)	8d
	H2A	-0.521(3)	-0.778(6)	0.020(8)	0.214(6)	18.0(13)	8d
	H2B	-0.475(3)	-0.712(6)	0.010(9)	0.214(6)	18.0(13)	8d
	H2C	-0.457(4)	-0.769(8)	0.090(4)	0.214(6)	18.0(13)	8d

Supplementary Table 10. Atomic parameters from the Rietveld refinement of TaS-1(0.013/1) at room temperature.

Species	Atom	*x*	*y*	*z*	SOF	B_{eq} (Å^2)	Wyckoff
TaS-1(0.013/1)	Si1	-0.0571(4)	-0.0737(3)	0.1651(5)	0.987167	1.193(16)	4e
	Si2	-0.0275(3)	-0.1858(4)	0.3103(4)	0.987167	1.193(16)	4e
	Si3	-0.4696(3)	-0.1879(4)	0.3316(4)	0.987167	1.193(16)	4e
	Si4	-0.0600(4)	-0.2229(3)	0.5293(5)	0.987167	1.193(16)	4e
	Si5	-0.4383(3)	-0.2207(3)	0.5483(4)	0.987167	1.193(16)	4e
	Si6	0.0567(3)	-0.1940(4)	0.6837(4)	0.987167	1.193(16)	4e
	Si7	-0.0639(4)	-0.3765(4)	0.5317(5)	0.987167	1.193(16)	4e
	Si8	-0.4442(4)	-0.0771(4)	0.1779(5)	0.987167	1.193(16)	4e
	Si9	0.0307(4)	-0.0761(3)	0.8254(5)	0.987167	1.193(16)	4e
	Si10	0.1324(3)	-0.1891(4)	0.3321(4)	0.987167	1.193(16)	4e
	Si11	-0.5605(3)	-0.1867(4)	0.6804(4)	0.987167	1.193(16)	4e
	Si12	-0.5279(4)	-0.0703(3)	0.8211(5)	0.987167	1.193(16)	4e
	Si13	0.1727(4)	-0.0797(3)	0.1835(5)	0.987167	1.193(16)	4e
	Si14	0.1687(4)	-0.2267(3)	0.5442(5)	0.987167	1.193(16)	4e
	Si15	-0.6285(3)	-0.1906(4)	0.3226(4)	0.987167	1.193(16)	4e
	Si16	-0.6729(4)	-0.0742(3)	0.1787(5)	0.987167	1.193(16)	4e
	Si17	-0.6744(4)	-0.2281(3)	0.5360(5)	0.987167	1.193(16)	4e
	Si18	-0.1287(4)	-0.0712(4)	0.8244(5)	0.987167	1.193(16)	4e
	Si19	-0.1763(4)	-0.1177(3)	0.0316(5)	0.987167	1.193(16)	4e
	Si20	-0.4371(4)	-0.3778(4)	0.5340(5)	0.987167	1.193(16)	4e
	Si21	-0.3712(4)	-0.0717(3)	0.8188(5)	0.987167	1.193(16)	4e
	Si22	-0.1659(3)	-0.1904(4)	0.6867(4)	0.987167	1.193(16)	4e
	Si23	-0.3212(3)	-0.1930(4)	0.6996(4)	0.987167	1.193(16)	4e
	Si24	-0.3287(4)	-0.1174(3)	0.0388(5)	0.987167	1.193(16)	4e
	Ta1	-0.0571(4)	-0.0737(3)	0.1651(5)	0.012833	1.193(16)	4e
	Ta2	-0.0275(3)	-0.1858(4)	0.3103(4)	0.012833	1.193(16)	4e
	Ta3	-0.4696(3)	-0.1879(4)	0.3316(4)	0.012833	1.193(16)	4e
	Ta4	-0.0600(4)	-0.2229(3)	0.5293(5)	0.012833	1.193(16)	4e
	Ta5	-0.4383(3)	-0.2207(3)	0.5483(4)	0.012833	1.193(16)	4e
	Ta6	0.0567(3)	-0.1940(4)	0.6837(4)	0.012833	1.193(16)	4e
	Ta7	-0.0639(4)	-0.3765(4)	0.5317(5)	0.012833	1.193(16)	4e
	Ta8	-0.4442(4)	-0.0771(4)	0.1779(5)	0.012833	1.193(16)	4e
	Ta9	0.0307(4)	-0.0761(3)	0.8254(5)	0.012833	1.193(16)	4e
	Ta10	0.1324(3)	-0.1891(4)	0.3321(4)	0.012833	1.193(16)	4e
	Ta11	-0.5605(3)	-0.1867(4)	0.6804(4)	0.012833	1.193(16)	4e
	Ta12	-0.5279(4)	-0.0703(3)	0.8211(5)	0.012833	1.193(16)	4e
	Ta13	0.1727(4)	-0.0797(3)	0.1835(5)	0.012833	1.193(16)	4e
	Ta14	0.1687(4)	-0.2267(3)	0.5442(5)	0.012833	1.193(16)	4e
	Ta15	-0.6285(3)	-0.1906(4)	0.3226(4)	0.012833	1.193(16)	4e
	Ta16	-0.6729(4)	-0.0742(3)	0.1787(5)	0.012833	1.193(16)	4e
	Ta17	-0.6744(4)	-0.2281(3)	0.5360(5)	0.012833	1.193(16)	4e
	Ta18	-0.1287(4)	-0.0712(4)	0.8244(5)	0.012833	1.193(16)	4e

	Ta19	-0.1763(4)	-0.1177(3)	0.0316(5)	0.012833	1.193(16)	4e
	Ta20	-0.4371(4)	-0.3778(4)	0.5340(5)	0.012833	1.193(16)	4e
	Ta21	-0.3712(4)	-0.0717(3)	0.8188(5)	0.012833	1.193(16)	4e
	Ta22	-0.1659(3)	-0.1904(4)	0.6867(4)	0.012833	1.193(16)	4e
	Ta23	-0.3212(3)	-0.1930(4)	0.6996(4)	0.012833	1.193(16)	4e
	Ta24	-0.3287(4)	-0.1174(3)	0.0388(5)	0.012833	1.193(16)	4e
	O1	-0.0511(8)	-0.1196(7)	0.2585(10)	1	1.03(3)	4e
	O2	-0.4389(8)	-0.1202(7)	0.2761(10)	1	1.03(3)	4e
	O3	-0.0540(6)	-0.1882(8)	0.4256(8)	1	1.03(3)	4e
	O4	-0.4329(5)	-0.1949(8)	0.4410(8)	1	1.03(3)	4e
	O5	-0.0636(5)	-0.2990(7)	0.5078(7)	1	1.03(3)	4e
	O6	0.0490(9)	-0.1160(7)	0.7335(10)	1	1.03(3)	4e
	O7	0.0659(7)	-0.1018(6)	0.9160(9)	1	1.03(3)	4e
	O8	0.0440(7)	-0.2456(6)	0.7665(10)	1	1.03(3)	4e
	O9	-0.5624(8)	-0.1179(8)	0.7429(10)	1	1.03(3)	4e
	O10	0.1557(8)	-0.1238(7)	0.2816(10)	1	1.03(3)	4e
	O11	-0.5585(8)	-0.2504(6)	0.7539(11)	1	1.03(3)	4e
	O12	0.1467(5)	-0.1839(6)	0.4420(8)	1	1.03(3)	4e
	O13	-0.6513(8)	-0.1197(7)	0.2745(9)	1	1.03(3)	4e
	O14	-0.1508(7)	-0.1212(6)	0.7478(9)	1	1.03(3)	4e
	O15	-0.6637(6)	-0.1891(8)	0.4252(8)	1	1.03(3)	4e
	O16	-0.1447(8)	-0.2483(6)	0.7470(10)	1	1.03(3)	4e
	O17	0.1554(5)	-0.3094(7)	0.5253(6)	1	1.03(3)	4e
	O18	0.0482(6)	-0.2023(7)	0.3102(8)	1	1.03(3)	4e
	O19	-0.1679(7)	-0.0930(6)	0.9199(10)	1	1.03(3)	4e
	O20	-0.1291(7)	-0.0806(6)	0.1121(9)	1	1.03(3)	4e
	O21	-0.3394(7)	-0.1251(7)	0.7405(8)	1	1.03(3)	4e
	O22	0.0007(7)	-0.0863(6)	0.0848(9)	1	1.03(3)	4e
	O23	-0.3333(7)	-0.2503(6)	0.7815(10)	1	1.03(3)	4e
	O24	-0.4516(7)	-0.0797(7)	0.8218(9)	1	1.03(3)	4e
	O25	-0.5427(6)	-0.1818(7)	0.3401(8)	1	1.03(3)	4e
	O26	0.1278(6)	-0.2014(8)	0.6358(8)	1	1.03(3)	4e
	O27	-0.0525(7)	-0.0794(7)	0.8370(9)	1	1.03(3)	4e
	O28	-0.4496(6)	-0.2987(7)	0.5446(7)	1	1.03(3)	4e
	O29	-0.3726(7)	-0.0870(7)	0.1225(9)	1	1.03(3)	4e
	O30	0.0009(6)	-0.2027(7)	0.5975(7)	1	1.03(3)	4e
	O31	-0.5040(7)	-0.1069(6)	0.1082(9)	1	1.03(3)	4e
	O32	-0.6583(5)	-0.3055(7)	0.5334(6)	1	1.03(3)	4e
	O33	0.1305(7)	-0.1023(6)	0.0832(9)	1	1.03(3)	4e
	O34	-0.1283(6)	-0.1986(8)	0.5738(8)	1	1.03(3)	4e
	O35	-0.6282(6)	-0.1877(7)	0.6207(8)	1	1.03(3)	4e
	O36	-0.5549(7)	-0.0836(6)	0.9324(9)	1	1.03(3)	4e
	O37	-0.5006(6)	-0.1829(6)	0.6067(8)	1	1.03(3)	4e
	O38	-0.0521(7)	-0.0013(8)	0.2058(9)	1	1.03(3)	4e

	O39	-0.3642(6)	-0.2078(7)	0.6066(8)	1	1.03(3)	4e
	O40	-0.3437(6)	-0.0822(6)	0.9347(9)	1	1.03(3)	4e
	O41	-0.4471(8)	-0.0013(8)	0.2070(9)	1	1.03(3)	4e
	O42	0.1448(7)	-0.0006(7)	0.2097(8)	1	1.03(3)	4e
	O43	-0.6482(7)	-0.0011(7)	0.2118(9)	1	1.03(3)	4e
	O44	-0.6309(7)	-0.0968(7)	0.0869(10)	1	1.03(3)	4e
	O45	0.2503(7)	-0.0720(6)	0.1522(9)	1	1.03(3)	4e
	O46	-0.2446(6)	-0.1990(7)	0.6603(7)	1	1.03(3)	4e
	O47	0.2478(7)	-0.2129(6)	0.5666(8)	1	1.03(3)	4e
	O48	-0.2530(8)	-0.1130(6)	0.0671(9)	1	1.03(3)	4e

Supplementary Table 11. Atomic parameters from the Rietveld refinement of MeOH@TaS-1(0.013/1) at room temperature.

Species	Atom	*x*	*y*	*z*	SOF	B_{eq} (Å^2)	Wyckoff
TaS-1(0.013/1)	Si1	-0.0572(3)	-0.0711(3)	0.1668(4)	0.987167	1.245(15)	4e
	Si2	-0.0289(3)	-0.1865(3)	0.3111(4)	0.987167	1.245(15)	4e
	Si3	-0.4688(3)	-0.1889(4)	0.3330(4)	0.987167	1.245(15)	4e
	Si4	-0.0595(3)	-0.2226(3)	0.5292(4)	0.987167	1.245(15)	4e
	Si5	-0.4386(3)	-0.2215(3)	0.5495(4)	0.987167	1.245(15)	4e
	Si6	0.0565(3)	-0.1934(4)	0.6882(4)	0.987167	1.245(15)	4e
	Si7	-0.0644(3)	-0.3789(3)	0.5323(4)	0.987167	1.245(15)	4e
	Si8	-0.4448(3)	-0.0794(3)	0.1786(5)	0.987167	1.245(15)	4e
	Si9	0.0298(3)	-0.0744(3)	0.8224(4)	0.987167	1.245(15)	4e
	Si10	0.1315(3)	-0.1867(4)	0.3303(4)	0.987167	1.245(15)	4e
	Si11	-0.5607(3)	-0.1864(3)	0.6791(4)	0.987167	1.245(15)	4e
	Si12	-0.5279(3)	-0.0713(3)	0.8192(4)	0.987167	1.245(15)	4e
	Si13	0.1732(3)	-0.0759(3)	0.1839(4)	0.987167	1.245(15)	4e
	Si14	0.1707(3)	-0.2279(3)	0.5450(4)	0.987167	1.245(15)	4e
	Si15	-0.6264(3)	-0.1889(4)	0.3224(4)	0.987167	1.245(15)	4e
	Si16	-0.6748(3)	-0.0755(3)	0.1793(4)	0.987167	1.245(15)	4e
	Si17	-0.6760(3)	-0.2280(3)	0.5354(4)	0.987167	1.245(15)	4e
	Si18	-0.1285(3)	-0.0714(3)	0.8266(4)	0.987167	1.245(15)	4e
	Si19	-0.1754(3)	-0.1189(3)	0.0335(4)	0.987167	1.245(15)	4e
	Si20	-0.4384(3)	-0.3757(3)	0.5374(4)	0.987167	1.245(15)	4e
	Si21	-0.3696(3)	-0.0727(3)	0.8234(5)	0.987167	1.245(15)	4e
	Si22	-0.1662(3)	-0.1888(3)	0.6866(4)	0.987167	1.245(15)	4e
	Si23	-0.3221(3)	-0.1927(4)	0.6984(4)	0.987167	1.245(15)	4e
	Si24	-0.3287(3)	-0.1163(3)	0.0364(4)	0.987167	1.245(15)	4e
	Ta1	-0.0572(3)	-0.0711(3)	0.1668(4)	0.012833	1.245(15)	4e
	Ta2	-0.0289(3)	-0.1865(3)	0.3111(4)	0.012833	1.245(15)	4e
	Ta3	-0.4688(3)	-0.1889(4)	0.3330(4)	0.012833	1.245(15)	4e
	Ta4	-0.0595(3)	-0.2226(3)	0.5292(4)	0.012833	1.245(15)	4e
	Ta5	-0.4386(3)	-0.2215(3)	0.5495(4)	0.012833	1.245(15)	4e
	Ta6	0.0565(3)	-0.1934(4)	0.6882(4)	0.012833	1.245(15)	4e
	Ta7	-0.0644(3)	-0.3789(3)	0.5323(4)	0.012833	1.245(15)	4e
	Ta8	-0.4448(3)	-0.0794(3)	0.1786(5)	0.012833	1.245(15)	4e
	Ta9	0.0298(3)	-0.0744(3)	0.8224(4)	0.012833	1.245(15)	4e
	Ta10	0.1315(3)	-0.1867(4)	0.3303(4)	0.012833	1.245(15)	4e
	Ta11	-0.5607(3)	-0.1864(3)	0.6791(4)	0.012833	1.245(15)	4e
	Ta12	-0.5279(3)	-0.0713(3)	0.8192(4)	0.012833	1.245(15)	4e
	Ta13	0.1732(3)	-0.0759(3)	0.1839(4)	0.012833	1.245(15)	4e
	Ta14	0.1707(3)	-0.2279(3)	0.5450(4)	0.012833	1.245(15)	4e
	Ta15	-0.6264(3)	-0.1889(4)	0.3224(4)	0.012833	1.245(15)	4e
	Ta16	-0.6748(3)	-0.0755(3)	0.1793(4)	0.012833	1.245(15)	4e
	Ta17	-0.6760(3)	-0.2280(3)	0.5354(4)	0.012833	1.245(15)	4e
	Ta18	-0.1285(3)	-0.0714(3)	0.8266(4)	0.012833	1.245(15)	4e

	Ta19	-0.1754(3)	-0.1189(3)	0.0335(4)	0.012833	1.245(15)	4e
	Ta20	-0.4384(3)	-0.3757(3)	0.5374(4)	0.012833	1.245(15)	4e
	Ta21	-0.3696(3)	-0.0727(3)	0.8234(5)	0.012833	1.245(15)	4e
	Ta22	-0.1662(3)	-0.1888(3)	0.6866(4)	0.012833	1.245(15)	4e
	Ta23	-0.3221(3)	-0.1927(4)	0.6984(4)	0.012833	1.245(15)	4e
	Ta24	-0.3287(3)	-0.1163(3)	0.0364(4)	0.012833	1.245(15)	4e
	O1	-0.0456(7)	-0.1180(7)	0.2580(9)	1	0.99(3)	4e
	O2	-0.4454(7)	-0.1235(6)	0.2683(9)	1	0.99(3)	4e
	O3	-0.0498(5)	-0.1876(7)	0.4237(7)	1	0.99(3)	4e
	O4	-0.4358(5)	-0.1962(7)	0.4414(7)	1	0.99(3)	4e
	O5	-0.0652(5)	-0.3012(6)	0.5044(6)	1	0.99(3)	4e
	O6	0.0485(7)	-0.1180(6)	0.7331(9)	1	0.99(3)	4e
	O7	0.0683(6)	-0.1031(5)	0.9193(8)	1	0.99(3)	4e
	O8	0.0473(7)	-0.2472(6)	0.7769(9)	1	0.99(3)	4e
	O9	-0.5645(6)	-0.1189(6)	0.7407(9)	1	0.99(3)	4e
	O10	0.1576(6)	-0.1186(6)	0.2836(8)	1	0.99(3)	4e
	O11	-0.5600(7)	-0.2467(6)	0.7513(9)	1	0.99(3)	4e
	O12	0.1482(5)	-0.1847(6)	0.4458(7)	1	0.99(3)	4e
	O13	-0.6562(6)	-0.1169(6)	0.2748(8)	1	0.99(3)	4e
	O14	-0.1514(6)	-0.1165(6)	0.7453(8)	1	0.99(3)	4e
	O15	-0.6646(5)	-0.1870(7)	0.4289(7)	1	0.99(3)	4e
	O16	-0.1403(6)	-0.2505(5)	0.7432(9)	1	0.99(3)	4e
	O17	0.1533(5)	-0.3087(7)	0.5231(6)	1	0.99(3)	4e
	O18	0.0505(6)	-0.1897(7)	0.3078(6)	1	0.99(3)	4e
	O19	-0.1656(6)	-0.0932(5)	0.9176(9)	1	0.99(3)	4e
	O20	-0.1301(6)	-0.0815(6)	0.1067(8)	1	0.99(3)	4e
	O21	-0.3367(6)	-0.1226(6)	0.7485(8)	1	0.99(3)	4e
	O22	-0.0019(6)	-0.0844(5)	0.0846(8)	1	0.99(3)	4e
	O23	-0.3312(6)	-0.2485(6)	0.7782(9)	1	0.99(3)	4e
	O24	-0.4487(6)	-0.0864(6)	0.8160(8)	1	0.99(3)	4e
	O25	-0.5469(6)	-0.1818(6)	0.3452(7)	1	0.99(3)	4e
	O26	0.1294(5)	-0.1978(7)	0.6403(7)	1	0.99(3)	4e
	O27	-0.0515(6)	-0.0782(6)	0.8340(8)	1	0.99(3)	4e
	O28	-0.4465(5)	-0.2968(6)	0.5427(6)	1	0.99(3)	4e
	O29	-0.3729(6)	-0.0846(6)	0.1176(7)	1	0.99(3)	4e
	O30	0.0027(5)	-0.2063(6)	0.6011(7)	1	0.99(3)	4e
	O31	-0.5058(6)	-0.1078(5)	0.1116(8)	1	0.99(3)	4e
	O32	-0.6569(4)	-0.3056(7)	0.5315(6)	1	0.99(3)	4e
	O33	0.1341(6)	-0.0989(6)	0.0807(8)	1	0.99(3)	4e
	O34	-0.1271(5)	-0.1956(7)	0.5827(7)	1	0.99(3)	4e
	O35	-0.6261(6)	-0.1857(6)	0.6157(7)	1	0.99(3)	4e
	O36	-0.5565(6)	-0.0828(5)	0.9292(8)	1	0.99(3)	4e
	O37	-0.4993(5)	-0.1849(6)	0.6057(7)	1	0.99(3)	4e
	O38	-0.0486(6)	-0.0000(6)	0.2041(8)	1	0.99(3)	4e

	O39	-0.3673(5)	-0.2081(6)	0.6043(7)	1	0.99(3)	4e
	O40	-0.3436(5)	-0.0829(5)	0.9303(8)	1	0.99(3)	4e
	O41	-0.4581(7)	-0.0007(7)	0.2078(8)	1	0.99(3)	4e
	O42	0.1483(7)	-0.0003(6)	0.2110(7)	1	0.99(3)	4e
	O43	-0.6517(6)	-0.0003(6)	0.2113(8)	1	0.99(3)	4e
	O44	-0.6359(6)	-0.0960(6)	0.0801(9)	1	0.99(3)	4e
	O45	0.2514(6)	-0.0757(5)	0.1552(8)	1	0.99(3)	4e
	O46	-0.2459(6)	-0.2009(6)	0.6569(7)	1	0.99(3)	4e
	O47	0.2462(6)	-0.2132(5)	0.5643(7)	1	0.99(3)	4e
	O48	-0.2516(7)	-0.1130(5)	0.0806(8)	1	0.99(3)	4e
$MeOH^{II}$	C1	-0.751(4)	-0.2992(13)	-0.182(3)	0.361(6)	15.0(13)	4e
	O1	-0.749(6)	-0.2313(17)	-0.154(7)	0.361(6)	15.0(13)	4e
	H1	-0.757(8)	-0.227(4)	-0.093(7)	0.361(6)	18.0(15)	4e
	H2A	-0.717(4)	-0.324(3)	-0.143(4)	0.361(6)	18.0(15)	4e
	H2B	-0.742(4)	-0.304(3)	-0.253(3)	0.361(6)	18.0(15)	4e
	H2C	-0.796(4)	-0.317(3)	-0.168(6)	0.361(6)	18.0(15)	4e
$MeOH^{III}$	C1	-0.329(3)	-1.026(3)	-0.545(3)	0.334(7)	15.0(13)	4e
	O1	-0.378(4)	-1.016(5)	-0.471(6)	0.334(7)	15.0(13)	4e
	H1	-0.371(5)	-1.042(7)	-0.423(5)	0.334(7)	18.0(15)	4e
	H2A	-0.284(3)	-1.017(3)	-0.517(5)	0.334(7)	18.0(15)	4e
	H2B	-0.337(3)	-0.995(4)	-0.601(5)	0.334(7)	18.0(15)	4e
	H2C	-0.331(4)	-1.072(3)	-0.570(6)	0.334(7)	18.0(15)	4e
$MeOH^{IV}$	C1	0.125(3)	0.989(3)	-0.452(4)	0.315(7)	15.0(13)	4e
	O1	0.070(4)	0.999(6)	-0.518(7)	0.315(7)	15.0(13)	4e
	H1	0.044(4)	1.028(6)	-0.495(12)	0.315(7)	18.0(15)	4e
	H2A	0.150(3)	1.031(4)	-0.445(6)	0.315(7)	18.0(15)	4e
	H2B	0.155(3)	0.955(4)	-0.479(7)	0.315(7)	18.0(15)	4e
	H2C	0.108(4)	0.975(5)	-0.387(4)	0.315(7)	18.0(15)	4e

Supplementary Table 12. Displaying the interatomic distances between the adsorption site and framework atoms of HZSM-5 and TaAlS-1, from the Rietveld refinements of the corresponding SXPD data at 25 °C.

Interatomic distances (Å)	HZSM-5(0.027/1)			TaAlS-1(0.013/0.027/1)		
	O-MeOH^{II}	O-MeOH^{III}	O-MeOH^{IV}	O-MeOH^{II}	O-MeOH^{III}	O-MeOH^{IV}
O1	4.6755(463)		4.6639(811)	4.9837(792)		5.6009(1230)
O2	3.5524(540)		5.1008(811)	3.8842(895)		5.9268(1171)
O3	3.6971(548)		7.1042(807)	4.2302(920)		6.9727(1362)
O4	3.8296(469)		5.3843(810)	4.2624(831)		5.7630(1221)
O5	4.1765(503)		3.6961(808)	4.4170(859)		3.9544(1391)
**O6**	**3.4990(577)**		5.5224(809)	**3.7254(957)**		5.4285(1222)
O7	5.8930(402)		4.2530(809)	5.9411(678)		4.0428(1100)
O8	6.0128(417)		3.7023(811)	6.0106(700)		3.188(111)
O9	5.0002(404)		5.6516(810)	5.0407(681)		4.957(104)
O10	7.0582(274)		6.8147(811)	7.0518(493)		7.0849(1074)
O11	6.2871(374)		5.1279(809)	6.2609(635)		5.3121(1072)
O12	5.1009(427)		6.3718(809)	5.5030(708)		5.6395(1071)
O13	5.6590(573)		5.1442(807)	5.9031(949)		5.1629(1288)
O14	5.9633(546)		5.8293(806)	6.3938(917)		6.4288(1440)
O15	5.3588(350)		5.0056(810)	5.6570(617)		5.6879(1107)
O16	5.9028(505)		6.3622(808)	6.0878(850)		7.1239(1262)
O17	3.8897(380)		6.8278(809)	4.1668(683)		6.6938(1104)
**O18**	3.772(45)		**3.1750(809)**	3.8263(777)		**3.0248(1197)**
O19	5.4913(533)		4.7898(808)	5.8465(890)		4.9144(1441)
O20	3.7563(430)		5.0212(812)	4.0565(737)		5.7419(1073)
O21	5.6383(404)		4.0977(808)	5.8262(707)		4.7599(1326)
O22	6.4610(343)		4.7492(809)	6.6021(601)		4.7975(1104)
O23	4.6273(371)		6.0266(814)	4.5702(606)		5.5644(1092)
O24	4.2744(407)		5.6861(816)	4.2519(643)		5.8331(1047)
O25	4.0835(431)		4.0348(813)	4.0116(683)		3.1717(1064)
O26	5.1763(359)		5.7509(815)	5.0958(576)		6.0934(1071)
T1		4.6855(1012)			4.3900(891)	
T2		4.3875(990)			4.2861(961)	
T3		4.6426(1003)			4.5603(1001)	
T4		6.4090(1063)			5.9994(947)	
T5		4.4435(1033)			**3.9920(916)**	
T6		4.4546(1026)			4.5602(934)	
T7		5.0723(958)			4.6343(850)	
T8		4.6962(970)			4.5020(943)	
T9		5.1966(917)			5.2966(919)	
T10		7.5665(1014)			7.2877(907)	
T11		4.6183(1005)			4.1850(894)	
T12		5.2321(928)			4.9439(900)	

Supplementary Table 13. Displaying the interatomic distances between the adsorption site and framework atoms of TaS-1, from the Rietveld refinements of the corresponding SXPD data at 25 °C.

TaS-1(0.013/1)	O-MeOH^{III}
T1	8.1290(809)
T2	7.7013(933)
T3	4.7066(920)
T4	7.5778(867)
T5	4.3164(992)
T6	7.5209(933)
T7	6.5969(845)
T8	5.0261(818)
T9	8.6062(814)
T10	6.6362(883)
T11	5.0747(939)
T12	5.0443(817)
T13	5.8701(820)
T14	6.4919(925)
T15	4.5772(974)
T16	4.4319(841)
T17	5.1008(994)
T18	6.4298(804)
T19	8.0647(821)
T20	7.3368(1003)
**T21**	**4.1033(823)**
T22	5.8400(877)
T23	4.3548(945)
T24	6.9733(825)

Supplementary Table 14. Summary of different types of Si atoms from the ^{29}Si NMR data and (Al+Ta)/Si ratio.

Chemical Shift (ppm)	Assignment[25,45,46]	Area (%)		(Al+Ta)/Si by ^{29}Si NMR[a]		(Al+Ta)/Si by EDX	
		HZSM-5	TaAlS-1	HZSM-5	TaAlS-1	HZSM-5	TaAlS-1
-113 to -117	$Si(OSi)_4$	85	79				
-105 to 108	$Si(OSi)_3(OAl/Ta)$	9	14	0.024	0.036	0.027	0.04
-103	$Si(OSi)_3(OH)$	6	7				

[a](Al+Ta)/Si = 0.25* $I_{Si(1Al)}/I$, where $I_{Si(1Al)}$ denotes the area of the NMR peak corresponding to the $Si(OSi)_3(OAl/Ta)$ atoms, and I denotes the total area of the ^{29}Si NMR peaks[24,47].

Supplementary Table 15. Different types of 1st neighbours and occurrence (#Num) in TaAlS-1, with chemical composition $Si_{93}Al_2TaO_{192}H_2$ calculated from ZeoTsites program.

Atom	Num(Si)	Num(Al)	Num(Ta)	#Num	Percentage (%)
Si	4	0	0	81	87
Si	3	1	0	8	13
Si	3	0	1	4	
Al	4	0	0	2	-
Ta	4	0	0	1	-

Supplementary Table 16. Contents of different Ta/Al/H sites on TaAlS-1 with chemical composition $Si_{93}Al_2TaO_{192}H_2$.

Ta/Al/H site	Position Ta	Position Al^I	O^I attached H	Position AlII	OII attached H
1	T5	T2	O6	T9	O18
2	T5	T2	O6	T9	O25
3	T5	T2	O6	T9	O8
4	T5	T2	O6	T9	O9
5	T5	T2	O1	T9	O18
6	T5	T2	O1	T9	O25
7	T5	T2	O1	T9	O8
8	T5	T2	O1	T9	O9
9	T5	T2	O2	T9	O18
10	T5	T2	O2	T9	O25
11	T5	T2	O2	T9	O8
12	T5	T2	O2	T9	O9
13	T5	T2	O13	T9	O18
14	T5	T2	O13	T9	O25
15	T5	T2	O13	T9	O8
16	T5	T2	O13	T9	O9
17	T5	T2	O6	T11	O10
18	T5	T4	O16	T9	O18
19	T8	T2	O6	T9	O18

Supplementary Table 17. Assignment of INS peaks of bare TaAlS-1.

Wavenumber in simulation (cm^{-1})	Wavenumber in experiment (cm^{-1})	Vibrational mode
58	53	OH out-of-plane bending
151	148	Ta-O bending
221	212	Zeolite framework
245	241	Zeolite framework
276	267	Zeolite framework + OH out-of-plane bending
305	304	Zeolite framework + OH out-of-plane bending
355	357	Zeolite framework + OH out-of-plane bending
450	435	Zeolite framework + OH out-of-plane bending
614	552	Zeolite framework
801	799	Zeolite framework
931	930	OH in-plane bending + Si-O stretching
1096	1090	OH in-plane bending
1218	1213	OH in-plane bending + Si-O stretching

Supplementary Table 18. Assignment of INS peaks of methanol.

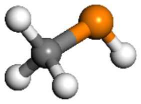		
Wavenumber in simulation (cm^{-1})	Wavenumber in experiment (cm^{-1})	Vibrational mode[b]
789 and 858	697 and 778	OH deformation
1141	1162	CH3 rocking
1451	1454	CH3 scissoring
2928	2955	CH3 stretching
3140	3241	OH stretching

Supplementary Table 19. Assignment of INS peaks of DME.

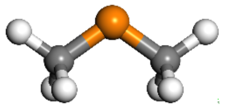

Wavenumber in simulation (cm^{-1})	Wavenumber in experiment (cm^{-1})	Vibrational mode
206	246	CH3 torsion
240	271	CH3 torsion
409	422	C-O-C scissoring
934	917	C-O-C symmetric stretching
1113	1096	C-O-C asymmetric stretching
1156	1155	CH3 rocking
1262	1251	CH3 rocking
1459	1449	CH3 wagging
1513	1522	CH3 scissoring
2970	2959	CH3 stretching

Supplementary Table 20. Assignment of INS peaks of TMO.

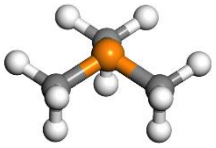

Wavenumber in simulation (cm^{-1})	Wavenumber in experimental spectrum of Reacted MeOH 2 (cm^{-1})	Vibrational mode
180	190	CH3 torsion
223	225	CH3 torsion
356	365	umbrella
399	410	C-O-C scissoring

Supplementary Table 21. Assignment of INS peaks of propene.

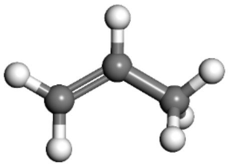

Wavenumber in simulation (cm^{-1})	Wavenumber in experiment (cm^{-1})	Vibrational mode
205	221	$-CH_3$ torsion
425	429	C=C-C scissoring
590	584	$C=CH_2$ torsion
941	916	$C=CH_2$ out-of-plane rocking
1024	997	=CH- out-of-plane rocking
1070	1044	$-CH_3$ out-of-plane rocking
1189	1161	$-CH_3$ in-plane rocking
1327	1289	=CH- in-plane rocking
1407	1371	-CH3 umbrella vibration
1447	1426	=CH2 scissoring
1480,1494	1453	-CH3 scissoring
1704	1644	C=C stretching
3013	2972	Sp3 CH stretching
3121	3062	Sp2 CH stretching

Supplementary References

1. Höfer, P., Grupp, A., Nebenführ, H. & Mehring, M. Hyperfine sublevel correlation (hyscore) spectroscopy: a 2D ESR investigation of the squaric acid radical. *Chem. Phys. Lett.* **132**, 279–282 (1986).
2. Hellring, S. D., Schmitt, K. D. & Chang, C. D. Synthesis and decomposition of trimethyloxonium ZSM-5, a purported intermediate in methanol conversion into gasoline. *J. Chem. Soc. Chem. Commun.* 1320–1322 (1987).
3. Clark, S. J. *et al.* First principles methods using CASTEP. *Z. Für Krist. - Cryst. Mater.* **220**, 567–570 (2005).
4. Refson, K., Tulip, P. R. & Clark, S. J. Variational density-functional perturbation theory for dielectrics and lattice dynamics. *Phys. Rev. B* **73**, 155114 (2006).
5. Perdew, J. P., Burke, K. & Ernzerhof, M. Generalized gradient approximation made simple. *Phys. Rev. Lett.* **77**, 3865–3868 (1996).
6. McNellis, E. R., Meyer, J. & Reuter, K. Azobenzene at coinage metal surfaces: role of dispersive van der Waals interactions. *Phys. Rev. B* **80**, 205414 (2009).
7. Cheng, Y. Q., Daemen, L. L., Kolesnikov, A. I. & Ramirez-Cuesta, A. J. Simulation of inelastic neutron scattering spectra using OCLIMAX. *J. Chem. Theory Comput.* **15**, 1974–1982 (2019).
8. Ramirez-Cuesta, A. J. aCLIMAX 4.0.1, The new version of the software for analyzing and interpreting INS spectra. *Comput. Phys. Commun.* **157**, 226–238 (2004).
9. Hutter, J., Iannuzzi, M., Schiffmann, F. & VandeVondele, J. cp2k: atomistic simulations of condensed matter systems. *Wiley Interdiscip. Rev. Comput. Mol. Sci.* **4**, 15–25 (2014).
10. Lippert, B. G., Hutter, J. & Parrinello, M. A hybrid Gaussian and plane wave density functional scheme. *Mol. Phys.* **92**, 477–488 (1997).
11. VandeVondele, J. *et al.* Quickstep: Fast and accurate density functional calculations using a mixed Gaussian and plane waves approach. *Comput. Phys. Commun.* **167**, 103–128 (2005).
12. VandeVondele, J. & Hutter, J. Gaussian basis sets for accurate calculations on molecular systems in gas and condensed phases. *J. Chem. Phys.* **127**, 114105 (2007).
13. Goedecker, S., Teter, M. & Hutter, J. Separable dual-space Gaussian pseudopotentials. *Phys. Rev. B* **54**, 1703–1710 (1996).
14. Grimme, S., Antony, J., Ehrlich, S. & Krieg, H. A consistent and accurate ab initio parametrization of density functional dispersion correction (DFT-D) for the 94 elements H-Pu. *J. Chem. Phys.* **132**, 154104 (2010).
15. Milman, V. *et al.* Structural, electronic and vibrational properties of tetragonal zirconia under pressure: a density functional theory study. *J. Phys. Condens. Matter* **21**, 485404 (2009).
16. Gonze, X., Charlier, J.-C., Allan, D. C. & Teter, M. P. Interatomic force constants from first principles: the case of alpha-quartz. *Phys. Rev. B* **50**, 13035–13038 (1994).

17. Hartmann, M., Ernst, S., Prakash, A. M. & Kevan, L. Characterization of mesoporous and microporous molecular sieves containing niobium and tantalum. *Stud. Surf. Sci. Catal.* **129**, 201–208 (2000).
18. Wichterlová, B., Nováková, J. & Prášil, Z. Structure of defects in γ-irradiated ZSM-5 and Y zeolites: an esr study. *Zeolites* **8**, 117–121 (1988).
19. Doorslaer, S. V. Hyperfine Spectroscopy: ESEEM. in *eMagRes* 51–70 (American Cancer Society, 2017). doi:10.1002/9780470034590.emrstm1517.
20. Yang, S. *et al.* Supramolecular binding and separation of hydrocarbons within a functionalized porous metal–organic framework. *Nat. Chem.* **7**, 121–129 (2015).
21. Ye, L. *et al.* Decarboxylation of lactones over Zn/ZSM-5: elucidation of the structure of the active site and molecular interactions. *Angew. Chem. Int. Ed.* **56**, 10711–10716 (2017).
22. Lo, B. T. W. *et al.* Elucidation of adsorbate structures and interactions on Brønsted acid sites in H-ZSM-5 by Synchrotron X-ray Powder Diffraction. *Angew. Chem. Int. Ed.* **55**, 5981–5984 (2016).
23. Hunger, M. & Horvath, T. Adsorption of methanol on Brønsted acid sites in zeolite H-ZSM-5 investigated by multinuclear solid-state NMR spectroscopy. *J. Am. Chem. Soc.* **118**, 12302–12308 (1996).
24. Sklenak, S. *et al.* Aluminium siting in the ZSM-5 framework by combination of high resolution 27Al NMR and DFT/MM calculations. *Phys. Chem. Chem. Phys.* **11**, 1237–1247 (2009).
25. Zhang, W. *et al.* Methane dehydro-aromatization over Mo/HZSM-5 in the absence of oxygen: a multinuclear solid-state NMR Study of the interaction between supported Mo species and HZSM-5 zeolite with different crystal sizes. *J. Catal.* **188**, 393–402 (1999).
26. Sastre, G. & Gale, J. D. ZeoTsites: a code for topological and crystallographic tetrahedral sites analysis in zeolites and zeotypes. *Microporous Mesoporous Mater.* **43**, 27–40 (2001).
27. Lemishko, T., Valencia, S., Rey, F., Jiménez-Ruiz, M. & Sastre, G. Inelastic neutron scattering study on the location of Brønsted acid sites in high silica LTA zeolite. *J. Phys. Chem. C* **120**, 24904–24909 (2016).
28. Lemishko, T. *et al.* Inelastic neutron scattering study of the aluminum and Brønsted site location in aluminosilicate LTA zeolites. *J. Phys. Chem. C* **122**, 11450–11454 (2018).
29. Parry, E. P. An infrared study of pyridine adsorbed on acidic solids. Characterization of surface acidity. *J. Catal.* **2**, 371–379 (1963).
30. Post, J. G. & van Hooff, J. H. C. Acidity and activity of H-ZSM-5 measured with NH_3-TPD and n-hexane cracking. *Zeolites* **4**, 9–14 (1984).
31. Hawkins, A. P. *et al.* Low-temperature studies of propene oligomerization in ZSM-5 by inelastic neutron scattering spectroscopy. *RSC Adv.* **9**, 18785–18790 (2019).
32. Hawkins, A. P. *et al.* Onset of propene oligomerization reactivity in ZSM-5 studied by inelastic neutron scattering spectroscopy. *ACS Omega* **5**, 7762–7770 (2020).

33. Yarulina, I. *et al.* Structure-performance descriptors and the role of Lewis acidity in the methanol-to-propylene process. *Nat. Chem.* **10**, 804–812 (2018).
34. Yoshioka, M., Yokoi, T. & Tatsumi, T. Development of the CON-type aluminosilicate zeolite and its catalytic application for the MTO reaction. *ACS Catal.* **5**, 4268–4275 (2015).
35. Zhao, X. *et al.* Investigation of methanol conversion over high-Si beta zeolites and the reaction mechanism of their high propene selectivity. *Catal. Sci. Technol.* **7**, 5882–5892 (2017).
36. Li, C. *et al.* Synthesis of reaction-adapted zeolites as methanol-to-olefins catalysts with mimics of reaction intermediates as organic structure-directing agents. *Nat. Catal.* **1**, 547–554 (2018).
37. Yang, M. *et al.* High propylene selectivity in methanol conversion over a small-pore SAPO molecular sieve with ultra-small cage. *ACS Catal.* **10**, 3741–3749 (2020).
38. Xu, Z. *et al.* Enhanced reaction lifetime of a bifunctional catalyst for methanol to olefins by combining formaldehyde decomposition on CeO_2. *Catal. Commun.* **141**, 106014 (2020).
39. Ferri, P. *et al.* Chemical and structural parameter connecting cavity architecture, confined hydrocarbon pool species, and MTO product selectivity in small-pore cage-based zeolites. *ACS Catal.* **9**, 11542–11551 (2019).
40. Xu, H. *et al.* Organosilane surfactant-assisted synthesis of mesoporous SSZ-39 zeolite with enhanced catalytic performance in the methanol-to-olefins reaction. *Front. Chem. Sci. Eng.* **14**, 267–274 (2020).
41. Wang, S. *et al.* Tuning the siting of aluminum in ZSM-11 zeolite and regulating its catalytic performance in the conversion of methanol to olefins. *J. Catal.* **377**, 81–97 (2019).
42. Teketel, S., Svelle, S., Lillerud, K.-P. & Olsbye, U. Shape-selective conversion of methanol to hydrocarbons over 10-ring unidirectional-channel acidic H-ZSM-22. *ChemCatChem* **1**, 78–81 (2009).
43. Kang, J. H., Alshafei, F. H., Zones, S. I. & Davis, M. E. Cage-defining ring: a molecular sieve structural indicator for light olefin product distribution from the methanol-to-olefins reaction. *ACS Catal.* **9**, 6012–6019 (2019).
44. Zhang, L., Wang, H., Liu, G., Gao, K. & Wu, J. Methanol-to-olefin conversion over H-MCM-22 catalyst. *J. Mol. Catal. Chem.* **411**, 311–316 (2016).
45. Cheng, Y., Miao, C., Hua, W., Yue, Y. & Gao, Z. Cr/ZSM-5 for ethane dehydrogenation: enhanced catalytic activity through surface silanol. *Appl. Catal. Gen.* **532**, 111–119 (2017).
46. Fyfe, C. A., Gobbi, G. C., Klinowski, J., Thomas, J. M. & Ramdas, S. Resolving crystallographically distinct tetrahedral sites in silicalite and ZSM-5 by solid-state NMR. *Nature* **296**, 530–533 (1982).
47. Engelhardt, G., Lohse, U., Lippmaa, E., Tarmak, M. & Mägi, M. ^{29}Si NMR-Untersuchungen zur verteilung der silicium-und aluminiumatome im alumosilicatgitter von zeolithen mit Faujasit-struktur. *Z. Für Anorg. Allg. Chem.* **482**, 49–64 (1981).
