# Supplementary material for: Control of zeolite microenvironment for propene synthesis from methanol
Source: Nat Commun. 2021 Feb 5;12:822. doi: 10.1038/s41467-021-21062-1 (PMC7865006; doi:10.1038/s41467-021-21062-1)
Supplement: Supplementary file 2 — Peer Review File [file 41467_2021_21062_MOESM2_ESM.pdf]

Reviewer #1 (Remarks to the Author):

The manuscript, "Control of zeolite microenvironment for propene synthesis from methanol" by Longfei Lin, et al. reports a new hetero-atomic zeolite catalyst for propene synthesis from methanol. The authors demonstrate high propene selectivity, high propene/ethene ratios, and high stability of the catalyst and elucidate the mechanism of catalytic reaction for propene synthesis over this new catalyst. I would like to recommend this manuscript for publication in Nature Communications after revisions. Comments and questions to authors should be addressed;

- Authors claimed that the catalyst shows homogeneous distribution of metal site. However, the EDX measurement seems done with aggregated zeolite particles so that it is not easy to check at single particle level. In addition, it is common ZSM-5 shows inhomogeneous Al distribution (Al poor core and Al rich shell).
- Authors listed used HZSM-5 and TaAlS-1 in Table S2 and compared them with pristine catalysts. Some data exhibit about 20% difference between pristine and used catalysts. I wonder if authors can claim they are similar. What is detection level of measurements? In addition, do the other catalysts show similar behaviors?
- In Table 1, there are 10 catalysts listed. However, mostly HZSM-5, TaAlS-1, and TaS-1 are displayed in figures of the manuscript. I wonder if there is any reason not to show experimental results on the others.
- Figure 1 is not clear to see lifetime and propene selectivity of each catalysts. It would be better if all catalysts have dashed line to bottom plane.
- The axis label (Conversion/Selectivity) and the legend (Conversion@TaAlS-1/HZSM-5) made me confused. They have to be clear.

Reviewer #2 (Remarks to the Author):

This manuscript reports the synthesis and characterization of new hetero-atomic TaAlS-1 zeolites by integrating Ta(V)and Al(III) sites into the MFI-zeolite frameworks, and its consequence in tuning the acidity and catalytic performance in the MTO reactions. With optimization of the microenvironments and acidity, TaAlS-1 exhibited a high propene selectivity, propene/ethene ratio and stability, comarable to the state-of-the-art zeolite catalysts. Based on the SXPD,INS and DFT studies,etc., the reaction mechanism for the first C-C bond formation in propene was also discussed. These novel results are important for understanding the structural requirements for the formation of propene from methanol and its reaction mechanism, and also for rational design of more efficient zeolite catalysts. Therefore, I think that this manuscript can be considered for publication in Nature Communication after considering the following comments.

1. As shown in Table 1, the MTO reaction is very sensitve to the Al content in the TaAlS-1 zeolites, and for example, the slight decrease in the Al/Si ratio from 0.027/1 to 0.02/1 led to the significant decrease in the propene selectivity from 51% to 18% (Entries 7 and 8). On the TaAlS-1(0.013/0.013/1)sample, the propene selectivity was as low as 1.9%. In order to provide fundamental understanding on such significant change, the corresponding characterization results for these samples, such as acidity, are requested to include and discuss in detail in the manuscript.

2. In this manuscript, the reaction mechanism, especially the TMO as a key intermediate for the formation of the first C-C bond, was discussed, but the conclusions or explanations were not convincing, based only on the the results of SXPD,INS and DFT,etc., because it is known clearly that the observation of the intermediates does not mean that they are reactive for forming the target products. Therefore, more relevent information and experimental evidence are necessary to confirm the reaction of the TMO species towards the formation of the olefin products.

3. About the discussion on the stability of the TaAlS-1 and HZSM-5 samples, as shown in Figure 2f, the reactions are requested to be carried out within kinetially-controlled regime, rather than at full conversions, in order to avoid the effect of the mass transfer and obtain the information about their intrinsic stability. The structures and acididity of the samples after the stability tests are requested to be characterized.

Responses to reviewers' reports NCOMMS-20-37792-T: *Control of zeolite microenvironment for propene synthesis from methanol*

We thank the reviewers for their constructive comments and our responses are given in ***bold italic*** for clarity.

Reviewer #1: The manuscript, "Control of zeolite microenvironment for propene synthesis from methanol" by Longfei Lin, et al. reports a new hetero-atomic zeolite catalyst for propene synthesis from methanol. The authors demonstrate high propene selectivity, high propene/ethene ratios, and high stability of the catalyst and elucidate the mechanism of catalytic reaction for propene synthesis over this new catalyst. I would like to recommend this manuscript for publication in Nature Communications after revisions. Comments and questions to authors should be addressed;

(1) Authors claimed that the catalyst shows homogeneous distribution of metal site. However, the EDX measurement seems done with aggregated zeolite particles so that it is not easy to check at single particle level. In addition, it is common ZSM-5 shows inhomogeneous Al distribution (Al poor core and Al rich shell).

Revised. This is an excellent point. We intended to state the absence of aggregation of metal sites, such as Ta-O-Ta and Al-O-Al clusters, in TaAlS-1. We apologise for the confusion caused and have revised the statement to 'The atomic ratio of Ta/Al/Si was determined by energy dispersive X-ray (EDX) analysis, which confirmed the existence of metal sites in the zeolite'. The distribution of Ta(V) and Al-O(H)-Si sites have been studied by ^{29}Si NMR, INS and DFT calculations (Supplementary Note Distribution of Ta/Al/H sites) and further confirmed by additional UV-vis spectrum and ^{27}Al NMR studies (Fig. 2d and Supplementary Fig. 4).

(2) Authors listed used HZSM-5 and TaAlS-1 in Table S2 and compared them with pristine catalysts. Some data exhibit about 20% difference between pristine and used catalysts. I wonder if authors can claim they are similar. What is detection level of measurements? In addition, do the other catalysts show similar behaviors?

Revised. The detection level of measurements is approximately 5-10%, which are contributed from sample loading, activation process, vacuum conditions etc. The data in Table S2 were determined from N_2 sorption isotherms and t-plots, which have been doubled checked and remeasured to confirm the presence of difference (Fig. R1). A close examination revealed minor changes between the pristine and used catalysts (Table R1). In addition, we have measured a TaAlS-1 catalyst used for 7 h and again minor difference was observed. In terms other catalysts, we have also measured the used TaS-1(0.013/1), where again minor difference was observed. We agree with the reviewer that the use of "similar" may not be critically accurate and this has been replaced with "minor difference" in the revised manuscript.

Table R1. Specific surface areas and pore volume of TaAlS-1, HZSM-5 and TaS-1 zeolites, data determined from N_2 sorption isotherms at 77 K and t-plots.

Samples	***A_{BET} ($m^2\ g^{-1}$)[a]***	***A_{mic} ($m^2\ g^{-1}$)[b]***	***A_{ext} ($m^2\ g^{-1}$)[c]***	***V_{mic} ($cm^3\ g^{-1}$)[d]***	***V_{total} ($cm^3\ g^{-1}$)[e]***
TaAlS-1(0.013/0.027/1)	***429***	***304***	***125***	***0.15***	***0.36***
TaAlS-1(0.013/0.027/1) used	***423***	***318***	***105***	***0.15***	***0.33***
TaAlS-1(0.013/0.027/1) remeasured	***424***	***320***	***104***	***0.15***	***0.32***
TaAlS-1(0.013/0.027/1) used 16 h	***431***	***328***	***103***	***0.16***	***0.32***

HZSM-5 (0.027/1)	***413***	***314***	***99***	***0.19***	***0.33***
HZSM-5 (0.027/1) used	***434***	***339***	***95***	***0.17***	***0.28***
HZSM-5 (0.027/1) remeasured	***433***	***335***	***99***	***0.17***	***0.29***
TaS-1(0.013/1)	***402***	***341***	***61***	***0.17***	***0.25***
TaS-1(0.013/1) used	***400***	***340***	***60***	***0.18***	***0.26***

[a]BET specific surface area; [b]Micropore surface area; [c]External surface area; [d]Micropore volume; [e]Total pore volume.

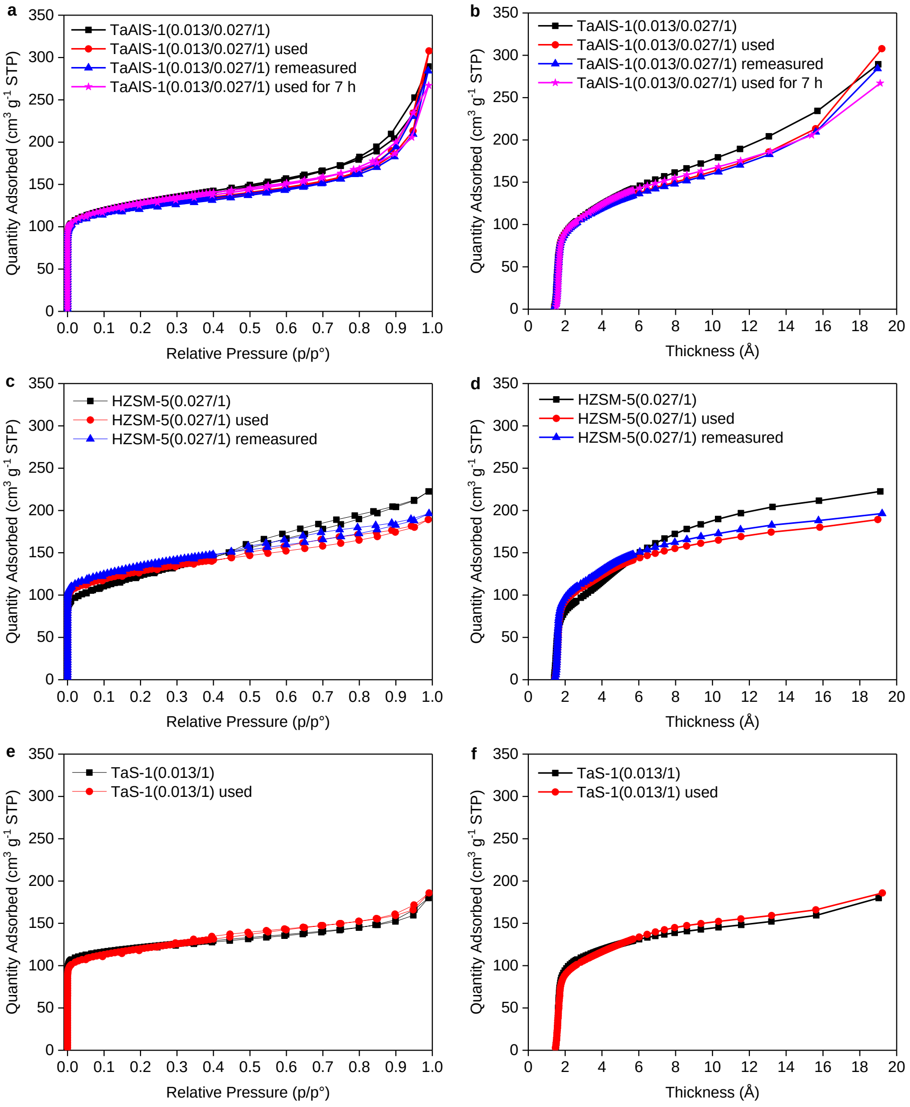

Fig. R1. N_2 sorption isotherms at 77 K (a, c, e) and t-plot (b, d, f) of fresh and used TaAlS-1 (a, b), HZSM-5 (c, d) and TaS-1 (e, f) zeolites.

(3) In Table 1, there are 10 catalysts listed. However, mostly HZSM-5, TaAlS-1, and TaS-1 are displayed in figures of the manuscript. I wonder if there is any reason not to show experimental results on the others.

Due to the limited beamtime available at National Facilities, we have focused on the deep investigation of three representative catalysts, namely HZSM-5 (containing Al), TaAlS-1 (containing both Al and Ta) and TaS-1 (containing Ta) in this work. Notable difference has been observed between these 3 samples, consistent with their catalytic performance, thus providing insights into the structure-activity relationship.

(4) Figure 1 is not clear to see lifetime and propene selectivity of each catalysts. It would be better if all catalysts have dashed line to bottom plane.
Revised. The projections to the bottom plan have been added.

(5) The axis label (Conversion/Selectivity) and the legend (Conversion@TaAlS-1/HZSM-5) made me confused. They have to be clear.
Revised. The axis label and legend in Fig. 2f have been modified to improve the clarity.

Reviewer #2: This manuscript reports the synthesis and characterization of new hetero-atomic TaAlS-1 zeolites by integrating Ta(V)and Al(III) sites into the MFI-zeolite frameworks, and its consequence in tuning the acidity and catalytic performance in the MTO reactions. With optimization of the microenvironments and acidity, TaAlS-1 exhibited a high propene selectivity, propene/ethene ratio and stability, comparable to the state-of-the-art zeolite catalysts. Based on the SXPD, INS and DFT studies, etc., the reaction mechanism for the first C-C bond formation in propene was also discussed. These novel results are important for understanding the structural requirements for the formation of propene from methanol and its reaction mechanism, and also for rational design of more efficient zeolite catalysts. Therefore, I think that this manuscript can be considered for publication in Nature Communication after considering the following comments.
(1) As shown in Table 1, the MTO reaction is very sensitive to the Al content in the TaAlS-1 zeolites, and for example, the slight decrease in the Al/Si ratio from 0.027/1 to 0.02/1 led to the significant decrease in the propene selectivity from 51% to 18% (Entries 7 and 8). On the TaAlS-1(0.013/0.013/1)sample, the propene selectivity was as low as 1.9%. In order to provide fundamental understanding on such significant change, the corresponding characterization results for these samples, such as acidity, are requested to include and discuss in detail in the manuscript.
Revised. These samples have been characterised by XRD, N_2 sorption and NH_3-TPD. Analysis of XRD and N_2 isotherms at 77 K confirmed the MFI structure and similar textual properties for both samples (Supplementary Fig. 1 and Supplementary Table 2). Compared with TaAlS-1(0.013/0.027/1), the acidity of TaAlS-1(0.013/0.02/1) and TaAlS-1(0.013/0.013/1) decreased to 0.11 and 0.05 mmol g^{-1}, respectively (Supplementary Table 5), leading to the production of DME as the primary product in the MTO reaction (Table 1, Entry 8). However, the distribution of propene among hydrocarbons produced over TaAlS-1(0.013/0.02/1) is 50.4%, which is similar to that obtained over TaAlS-1(0.013/0.027/1). This result suggests that over TaAlS-1(0.013/0.02/1), the conversion of DME to olefins is significantly hindered while the transformation of ethene to higher olefins is affected to a less extent, further indicating that the formation of the first carbon-carbon is the limiting step. Further reduction of Al(III) sites restricts the formation of first carbon-carbon bonds and hence the following reactions, leading to the low selectivity of propene (Table 1, Entry 9). Thus, a suitable amount of Al(III) sites is essential for the production of propene. These new results and discussions have been added in the revised manuscript.

(2) In this manuscript, the reaction mechanism, especially the TMO as a key intermediate for the formation of the first C-C bond, was discussed, but the conclusions or explanations were not convincing，based only on the results of SXPD,INS and DFT, etc., because it is known clearly that the observation of the intermediates does not mean that they are reactive for forming the target products. Therefore, more relevent information and experimental evidence are necessary to confirm the reaction of the TMO species towards the formation of the olefin products.
Revised. This is an excellent point that deserves further investigations. Additional experiments based upon

temperature-programmed mass spectroscopy (TP-MS) have been conducted to study the reactivity of TMO ($C_3H_9O^+$) for the formation of olefins within the zeolites (Supplementary Note Temperature-programmed mass spectroscopy, Supplementary Figs. 50,51). Firstly, TMO was dosed to TaAlS-1 to afford TMO@TaAlS-1, and part of the sample was then loaded with CH_3OH or CD_3OD. The systems were heated to 400 ^oC at 10 ^oC/min under a helium flow and the efflux monitored by online MS. Heating TMO@TaAlS-1+CD_3OD at 292 ^oC yielded a series of MS signals of m/z at 42, 45, 46 and 48 (Supplementary Fig. 51a-b). The signal of m/z 45 corresponds to $C_3H_3D_3$, which was produced via the reaction between adsorbed TMO and CD_3OD species (Supplementary Fig. 50). It is worth noting that the signal of m/z 45 is significantly decreased or indeed absent in the reaction of TMO@TaAlS-1 or TaAlS-1+CD_3OD at 292 °C (Supplementary Fig. 51c,d), thus confirming that the reaction between adsorbed TMO and CD_3OD species plays a key role in the formation of C-C bonds in propene. C_3D_6 (m/z 48) was produced from CD_3OD over TaAlS-1 (Supplementary Fig. 51b,d). The signal of m/z 46 could be originated from fragments of C_3D_6 or $C_3H_2D_4$ (Supplementary Fig. 50). Moreover, the formation of CH_3OCH_3 or CD_3OCD_3 peak at around 270 °C (Supplementary Figs. 51b,d,e). The formation of propene in the system of TaAlS-1+CD_3OD (Supplementary Fig. 51d) peaks at 350 ^oC, whereas it peaks at 292 ^oC in both systems of TMO@TaAlS-1+CD_3OD (Supplementary Fig. 51b) and TMO@TaAlS-1+CH_3OH (Supplementary Fig. 51e). Thus, this result demonstrates that the presence of TMO in the pore can promote the formation of olefins. These new results and analysis have been added to the revised manuscript.

(3) About the discussion on the stability of the TaAlS-1 and HZSM-5 samples, as shown in Figure 2f, the reactions are requested to be carried out within kinetically-controlled regime, rather than at full conversions, in order to avoid the effect of the mass transfer and obtain the information about their intrinsic stability. The structures and acidity of the samples after the stability tests are requested to be characterized.

Revised. We agree with the reviewer and the stability within kinetically-controlled regime has been conducted under the reaction conditions: W/F of 2.83 h g_{cat} g_{MeOH}^{-1}, particle size of 250-420 μm and conversion of 94%, and the external and internal mass transform for methanol-TaAlS-1(0.013/0.027/1) studied (Supplementary Figs. 9-11). The conversion on TaAlS-1(0.013/0.027/1) decreased rapidly at these conditions. The used catalyst was calcined under an air flow at 600 ^oC to remove the coke and characterised by N_2 adsorption, XRD and NH_3-TPD. The textural properties, framework structure and acidity remain largely intact (Supplementary Table 2, Supplementary Fig. 12), indicating that the deactivation was caused by rapid coke formation. Lercher et al. identified two pathways of coke formation (J. Am. Chem. Soc. 2016, 138, 15994–16003). In the absence of methanol, coke formation occurs on Brønsted acid sties via hydrogen transfer between olefins and naphthenes, while in the present of methanol, the hydrogen transfer occurs firstly on Lewis acid sites, followed by further reactions on Brønsted acid sites to form coke. The latter process is much faster. Thus, the TaAlS-1 zeolite with high ratio of Lewis/Bronsted acid sites exhibits fast deactivation in the present of methanol. It is worth noting that almost all stability studies on MTO reactions in the literature were conducted at full conversion (Supplementary Table1), and the corresponding tests at full conversions are used to enable a direct comparison with the literature examples. The new tests within the kinetically-controlled regime and additional discussions have been added to the revised manuscript.

Reviewer #2 (Remarks to the Author):

I appreciate all of author's efforts to improve this manuscript. This revised manuscript becomes clearer than the original. Additional experimental results also support this revised version well. I would like to recommend this manuscript for publication in Nature Communications. One comment is in the following;

- Authors need to check typos, e.g. additional '(' in line 95.

Reviewer #4 (Remarks to the Author):

Authors reports a new strategy to control the nature of active sites by incorporating tantalum and Aluminium centres into a framework and they clearly show that. The relevance of the work is also beyond doubt. Authors also took into account comments of referee, therefore I can recomend to publish this manuscript. I have only a couple of minor comments:
- At the fig.1 different point have a different colors. It is not clear for me what deos it mean, nothing in figure capture and in the text.
- Fig.3 show a visualization of crystal structures. it is almost impossible to recognize anything in such very small drawings and I would recomend eighther to redraw fig. 3 or move it in more detail view in Supplement.
-Fig.4: please move the legend a little bit on left side, marks in the legens are nixed with marks at the picture
- The Supplement is almost 100 pages and I find it too big, especially in the part on INS. A lot of raw figures are presented (correction to empty can etc.) These pictures show only the process of data refinement but have no any additional scientific information.

Responses to reviewers' reports NCOMMS-20-37792A: *Control of zeolite microenvironment for propene synthesis from methanol*

We thank the reviewers for their comments and our responses are given in ***bold italic*** for clarity.

Reviewer #2: I appreciate all of author's efforts to improve this manuscript. This revised manuscript becomes clearer than the original. Additional experimental results also support this revised version well. I would like to recommend this manuscript for publication in Nature Communications. One comment is in the following;

- Authors need to check typos, e.g. additional '(' in line 95.

Revised. This has been removed.

Reviewer #4: Authors reports a new strategy to control the nature of active sites by incorporating tantalum and Aluminium centres into a framework and they clearly show that. The relevance of the work is also beyond doubt. Authors also took into account comments of referee, therefore I can recomend to publish this manuscript. I have only a couple of minor comments:

- At the fig.1 different point have a different colors. It is not clear for me what deos it mean, nothing in figure capture and in the text.

Revised. The colour scheme has been modified, and each spot has a different colour to identify the corresponding catalyst.

- Fig.3 show a visualization of crystal structures. it is almost impossible to recognize anything in such very small drawings and I would recomend eighther to redraw fig. 3 or move it in more detail view in Supplement.

Revised. Enlarged and detailed views have been added in the Supplementary Materials (Supplementary Fig. 17).

-Fig.4: please move the legend a little bit on left side, marks in the legends are nixed with marks at the picture

Revised.

- The Supplement is almost 100 pages and I find it too big, especially in the part on INS. A lot of raw figures are presented (correction to empty can etc.) These pictures show only the process of data refinement but have no any additional scientific information.

The raw figures in the Supplementary Materials are given to provide full information of data processing to readers as required by Nat. Commun..
